# Sequential Alterations in Catabolic and Anabolic Gene Expression Parallel Pathological Changes during Progression of Monoiodoacetate-Induced Arthritis

**DOI:** 10.1371/journal.pone.0024320

**Published:** 2011-09-13

**Authors:** Jin Nam, Priyangi Perera, Jie Liu, Bjoern Rath, James Deschner, Robert Gassner, Timothy A. Butterfield, Sudha Agarwal

**Affiliations:** 1 The Biomechanics and Tissue Engineering Laboratory, College of Dentistry, The Ohio State University, Columbus, Ohio, United States of America; 2 Department of Orthopedic Surgery, University of Aachen, Aachen, Germany; 3 Department of Periodontics, University of Köln, Köln, Germany; 4 Department of Oral and Maxillofacial Surgery, University of Innsbruck College of Medicine, Innsbruck, Austria; 5 Rehabilitation Sciences, College of Health Sciences, University of Kentucky, Lexington, Kentucky, United States of America; 6 Department of Orthopedics, The Ohio State University, Columbus, Ohio, United States of America; South Texas Veterans Health Care System, United States of America

## Abstract

Chronic inflammation is one of the major causes of cartilage destruction in osteoarthritis. Here, we systematically analyzed the changes in gene expression associated with the progression of cartilage destruction in monoiodoacetate-induced arthritis (MIA) of the rat knee. Sprague Dawley female rats were given intra-articular injection of monoiodoacetate in the knee. The progression of MIA was monitored macroscopically, microscopically and by micro-computed tomography. Grade 1 damage was observed by day 5 post-monoiodoacetate injection, progressively increasing to Grade 2 by day 9, and to Grade 3–3.5 by day 21. Affymetrix GeneChip was utilized to analyze the transcriptome-wide changes in gene expression, and the expression of salient genes was confirmed by real-time-PCR. Functional networks generated by Ingenuity Pathways Analysis (IPA) from the microarray data correlated the macroscopic/histologic findings with molecular interactions of genes/gene products. Temporal changes in gene expression during the progression of MIA were categorized into five major gene clusters. IPA revealed that Grade 1 damage was associated with upregulation of acute/innate inflammatory responsive genes (*Cluster I*) and suppression of genes associated with musculoskeletal development and function (*Cluster IV*). Grade 2 damage was associated with upregulation of chronic inflammatory and immune trafficking genes (*Cluster II*) and downregulation of genes associated with musculoskeletal disorders (*Cluster IV*). The Grade 3 to 3.5 cartilage damage was associated with chronic inflammatory and immune adaptation genes (*Cluster III*). These findings suggest that temporal regulation of discrete gene clusters involving inflammatory mediators, receptors, and proteases may control the progression of cartilage destruction. In this process, IL-1β, TNF-α, IL-15, IL-12, chemokines, and NF-κB act as central nodes of the inflammatory networks, regulating catabolic processes. Simultaneously, upregulation of asporin, and downregulation of TGF-β complex, SOX-9, IGF and CTGF may be central to suppress matrix synthesis and chondrocytic anabolic activities, collectively contributing to the progression of cartilage destruction in MIA.

## Introduction

Osteoarthritis (OA) is a debilitating joint disease, causing severe pain and physical disabilities to millions of people worldwide [1], [2], [3]. The etiopathology of OA is multifactorial. Chronic inflammation, degeneration of the extracellular matrix and abnormal remodeling of the underlying bone, all take part in cartilage destruction [4], [5], [6], [7]. Cartilage matrix is mainly composed of collagens and proteoglycans synthesized by chondrocytes residing in the matrix. Collagens, mainly Type II, type IX and type XI, provide the tensile strength, whereas proteoglycans rich in water act as shock absorbents during cartilage loading.

At the onset of the disease, an etiologic agent or an insult/trauma of the joint causes focal edema and minor surface erosion in the cartilage. Progression of cartilage destruction is marked by further fibrillation accompanied by loss of matrix and chondrocytes in the superficial layers of the cartilage. Proliferating chondrocytes become apparent as cell clusters in the middle zone of the cartilage. Further progression of fibrillation and cartilage erosion leads to increased loss of cartilage matrix resulting in bone denudation, the hallmark of osteoarthritic lesions. The rate of disease progression and amount of joint damage is not predictable, and varies among patients. However, the progression of joint damage appears to follow a similar pattern and therefore can be categorized into various grades according to the extent of damage in the cartilage and bone [8], [9].

Chondrocytes take part in cartilage damage by synthesizing catabolic cytokines and enzymes that breakdown the matrix as well as impairing their ability to repair the matrix. Studies focusing on the molecular events in human OA inception and progression by genomic or proteomic profiling of intra-articular lesions have revealed distinct gene profiles in OA specimens as compared to visually unaffected cartilage [10], [11], [12], [13], [14], [15]. Similarly, gene association studies in large populations have identified a number of genes that might confer susceptibility to OA [16], [17], [18], [19]. However, the knowledge of the discrete molecular events that support the time-dependent progression of OA remains incomplete.

In this study, we aimed to conduct a systematic longitudinal examination of molecules and pathways associated with the progression of cartilage damage. A widely used model of monoiodoacetate-induced arthritis (MIA) of the rat knee was utilized [20], [21]. The progression of MIA was analyzed by macroscopic, microscopic and micro-tomographic (µCT) analyses and categorized into various stages of cartilage damage using the grading system of Pritzker *et al.* [9]. A transcriptome-wide analysis was conducted on the cartilage of temporally well-defined stages of MIA and compared to those of sham control cartilage. Ingenuity Pathways Analysis (IPA) was employed to obtain key insights into molecular relationships and networks/mechanisms during the progression of cartilage destruction. This analysis linked the microarray data to relevant, manually curated information from periodically updated knowledge databases in order to interpret the global impact of differentially regulated molecules during MIA progression. We believe that this study is the first to systematically elucidate the longitudinal time-dependent gene regulation and molecular networks/mechanisms throughout the course of MIA progression and cartilage destruction.

## Results

### Macroscopic and microscopic changes in cartilage and subchondral bone during the progression of MIA

**Figure 1:**
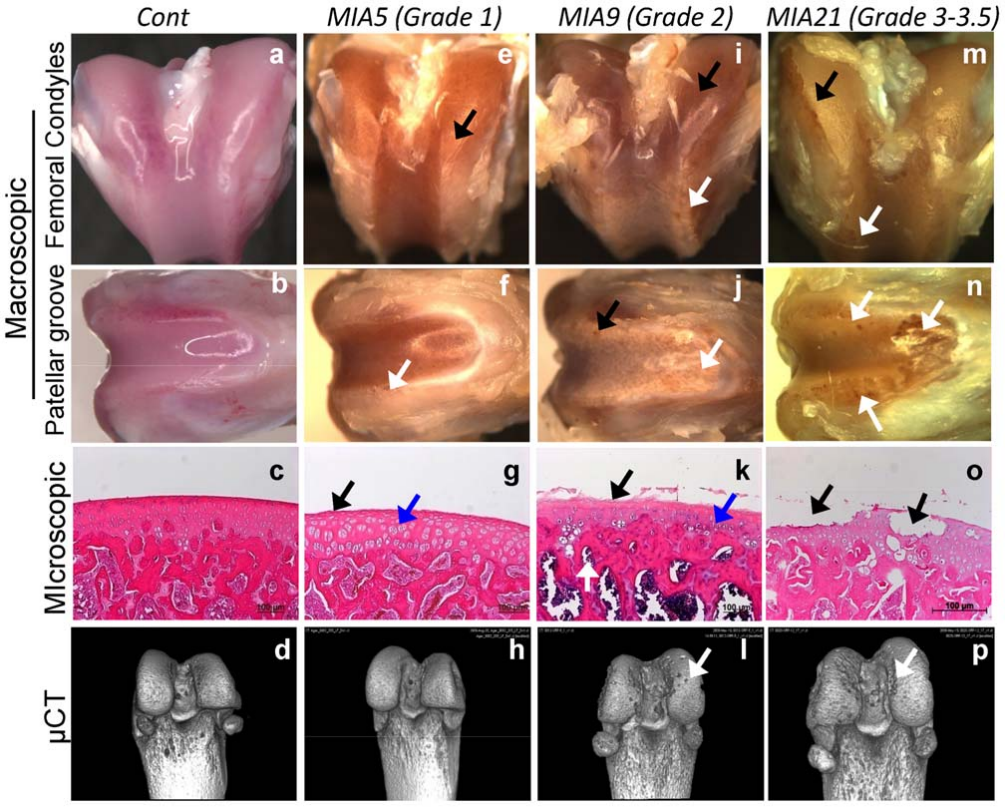
Progression of MIA at the distal femoral ends by macroscopic, microscopic, and µCT analyses. Right knees of rats were given an intra-articular injection of MIA on day 0, and distal ends of right femurs examined on post-injection days 5 (Grade 1 damage, *MIA5*), 9 (Grade 2 damage, *MIA9*) and 21 (Grade 3–3.5 damage, *MIA21*) and compared to saline-injected sham control (*Cont*). Macroscopic view of condyles, patellar grooves of cartilage, histology, and subchondral bone imaging by µCT of: (a, b) *Cont* femur showing smooth surface, (c) normal histology and no bone lesions on the femoral condyles and patellar grove and (d) lack of lesions in the subchondral bone (Movie S1); (e, f) *MIA5* cartilage showing superficial abrasions on the condyles (black arrows) and patellar groove (white arrows), (g) superficial fibrillation (black arrow), chondrocyte clustering and disorientation (blue arrow), and (h) no bone lesions in µCT images (Movie S2); (i, j) *MIA9* cartilage exhibiting lesions at the apexes of condyles (black arrow) and ridges of the patellar groove (white arrow), (k) thinning of cartilage, matrix and cell loss above the tidal layer with large disarrayed chondrocytes (black arrow), and some multinucleated chondrocytes (blue arrow), subchondral bone marrow/fibrous tissue extension in the cartilage typical of Grade 2 damage (white arrow), and (l) scattered subchondral bone lesions on the femoral condyles and patellar groove in µCT images (Movie S3); (m, n) *MIA21* cartilage exhibiting increased lesions and damage on the condyles (black arrows) and patellar groove and ridges (white arrow), (o) delamination of surface, full depth cartilage lesions and denuded cartilage layer at some places (black arrow), and (p) increased subchondral bone lesions on the femoral condyles and patellar groove in µCT images (Movie S4). Each figure shows representative right femur from separate rats from each group (n = 10). Arrows indicate cartilage damages. The distal ends of femurs showing 360° µCT projection can be found in Movie files S1 to S4.

The progression of MIA was monitored by overall macroscopic and microscopic changes at the distal ends of femurs (Figure 1). The articular surface of *Cont* femurs exhibited normal cartilage morphology, histology and bone imaging by µCT, typical of Grade 0/healthy cartilage (Figure 1 a–d, Movie S1). The progression of MIA followed the similar pathologies as described by Guzman *et al.* [22]. Typically, femurs from MIA afflicted knees exhibited greater extent of cartilage damage around the patellar groove than on femoral condyles and intercondylar fossa (Figure 1 e, f, i, j, m, n). The examination of time-dependent progression of knee cartilage damage showed that, on day 5 post MIA induction (*MIA5*), femurs showed cartilage damage typical of Grade 1, i.e., superficial fibrillation, chondrocyte proliferation, clustering and disorientation, and some loss of tidal ridge demarcation (Figure 1e–g) [9], [22]. Bone damage was not apparent microscopically or by µCT imaging at both patellar and condylar surfaces (Figure 1e–h, Movie S2).

Analysis of *MIA9* cartilage revealed marked lesions at the apexes of condyles and ridges of the patellar groove (Figure 1i–k). The loss of the tidal layer and deeper lesions in some areas were observed. Chondrocytes appeared larger, some with multiple nuclei and disarrayed. Subchondral bone marrow extensions towards cartilage and deposition of fibrous tissue in the lesions typical of Grade 2 cartilage degeneration were apparent. The µCT images revealed scattered subchondral bone lesions on the femoral condyles and patellar groove (Figure 1l, Movie S3).

On day 21 post-monoiodoacetate injection (*MIA21*), increased cartilage and bone damage in the patellar groove and ridges, full-depth lesions and pits on the femoral condyles were observed (Figure 1m–o). Histology revealed fissuring with matrix loss, fibrocartilage formation within the denuded cartilage and abnormal subchondral bone marrow intrusion typical of Grade 3 to 3.5 damage. Micro-CT imaging showed pitted areas of bone loss on the femoral condyles and patellar groove (Figure 1p, Movie S4).

### Transcriptome-wide regulation of gene expression during the progression of MIA

**Figure 2:**
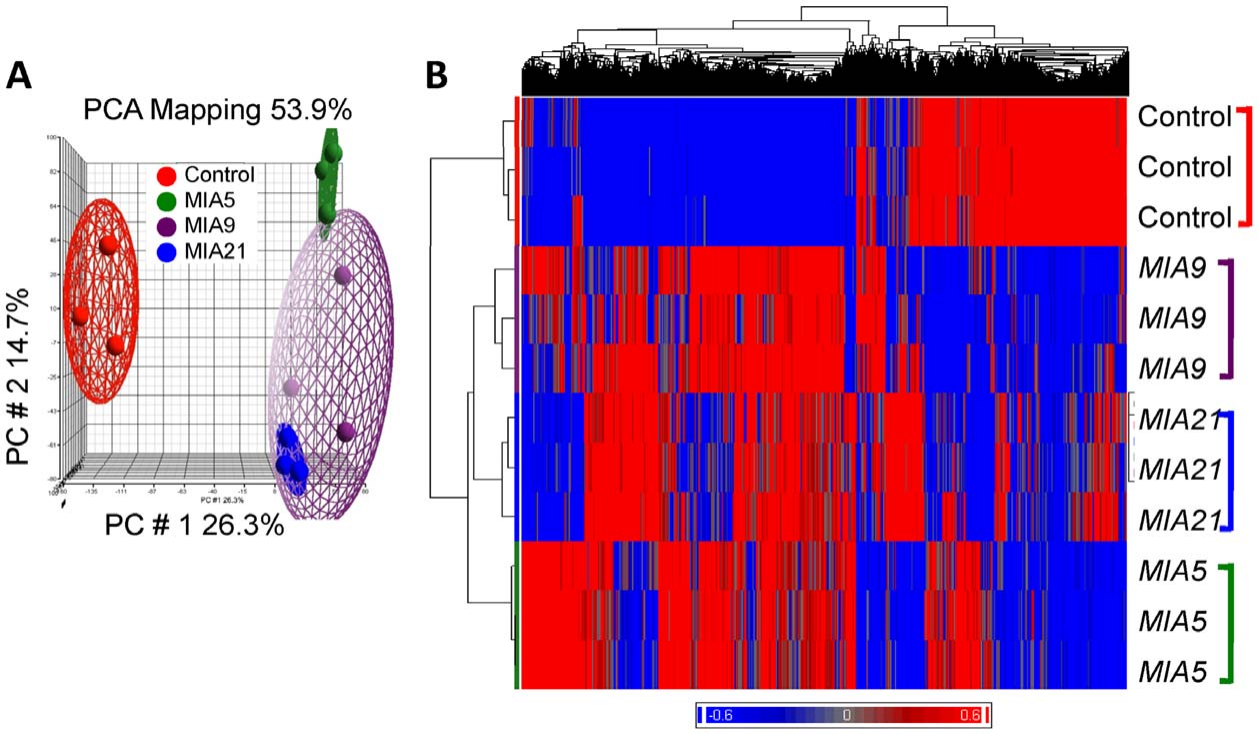
Transcriptome-wide microarray analysis of cartilage from *Cont*, *MIA5*, *MIA9*, or *MIA21* afflicted joints. (A) PCA analysis showing reproducible overall gene expression in the articular cartilage from the right knee joint of 3 separate rats from *Cont*, *MIA5*, *MIA9*, or *MIA21*. (B) Overall gene expression profiles of articular cartilage from 3 separate rats in each experimental group as compared to *Cont*. Hierarchical clustering representing the transcripts that were significantly (*p*<0.05) and differentially up- or downregulated at one or more time points by more than two-fold change. Note the maximal changes in overall gene expression occurred in MIA5, followed by MIA 21 and MIA9 as compared to gene expression in cont cartilage.

We next determined the changes in transcriptome-wide gene expression profiles during the progression of MIA in the distal end of femoral cartilages in *Cont*, *MIA5*, *MIA9* and *MIA21* rats exhibiting Grade 0, Grade 1, Grade 2 and 3–3.5 cartilage damage, respectively. Principal components analysis (PCA) revealed relatively uniform distribution of overall gene expression among the samples in each group (n = 3) except in *MIA9* group, where the overall gene expression was distributed between *MIA5* and *MIA21* (Figure 2A). Significant differences in gene expression over the course of MIA progression were observed, as evidenced by the average F ratio (signal to noise ratio) of 18.8.

Of the 27,342 transcripts detectable by Affymetrix GeneChips array, 2,034 (7.44%) transcripts were significantly (*p*<0.05) and differentially up- or downregulated at one or more time points by more than two-fold change. In the hierarchical clustering analysis of the differentially regulated genes (*p*<0.05, over ±2-fold change), distinct sets of genes were regulated at each stage of MIA progression (Figure 2B). The most interesting information derived from the hierarchical clustering was that: (i) as compared to *Cont*, the maximal changes in gene expression occurred in *MIA5*, judging by its farthest distance from *Cont* (Figure 2B), followed by *MIA21* and *MIA9*; and (ii) distinct individual sets of genes were temporally either upregulated or suppressed during the progression of MIA.

### Cluster analysis of major functional genes during the progression of MIA

**Figure 3:**
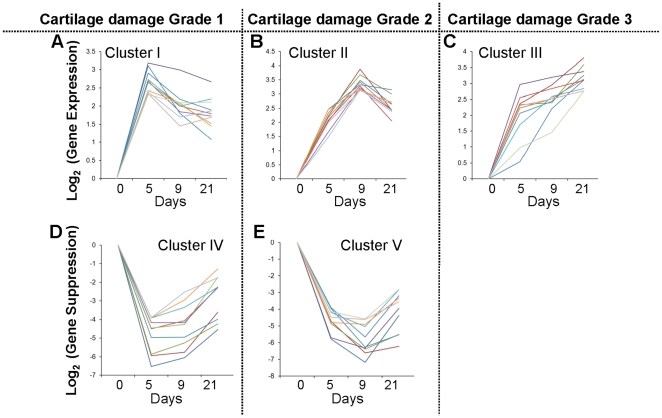
Partition clustering of significantly regulated genes. Partition clustering analysis of the genes that showed two fold or greater changes in their expression at one or more time points (p<0.05). The graphs represent 10 most regulated genes in each cluster. Identification of five gene clusters that exhibited maximal upregulation on day 5 (Grade 1 damage) followed by their downregulation (Cluster I); upregulation on day 9 (Grade 2 damage) followed by their downregulation (Cluster II); upregulation in a sustained manner showing maximal expression on day 21 (Grade 3–3.5 damage, Cluster III); downregulation of genes on day 5 followed by their upregulation (Cluster IV); and downregulation of genes on day 9 followed by their upregulation (Cluster V). Detailed description of these genes is given in Tables 1, 2, 3, 4, 5, and 6, and in Tables S1, S2, S3, S4, and S5.

**Figure 4:**
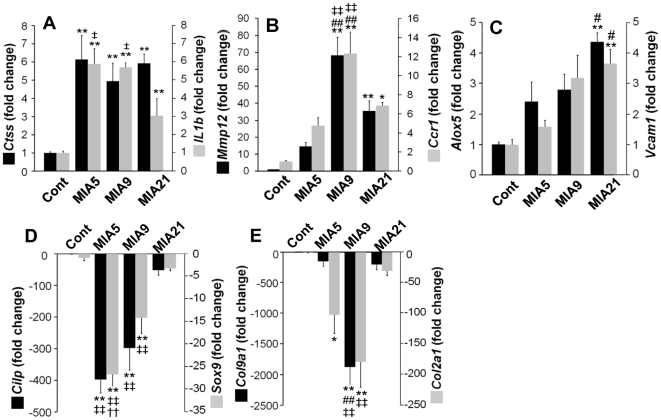
Confirmation of salient gene expression by rt-PCR. Quantitative rt-PCR analyses of specific transcripts of genes from articular cartilage obtained from *Cont*, *MIA5*, *MIA9*, or *MIA21*. Two genes from *Cluster I* (A; *Ctss* and *Il1b*), *Custer II* (B; *Mmp12* and *Ccr1*), *Cluster III* (C; *Alox5* and *Vcam1*), *Cluster IV* (D; *Cilp* and *Sox9*), and *Cluster V* (E; *Col9a1* and *Col2a1*) were analyzed to verify the data obtained by microarray analysis (n = 5, * *p*<0.05 as compared to *Cont*, ** *p*<0.01 as compared to *Cont*, # *p*<0.05 as compared to *MIA5*, ## *p*<0.01 as compared to *MIA5*, † *p*<0.05 as compared to *MIA9*, †† *p*<0.01 as compared to *MIA9*, ‡ *p*<0.05 as compared to *MIA21*, ‡‡ *p*<0.01 as compared to *MIA21*).

Among the 2,034 transcripts that were significantly up- or downregulated during the progression of MIA, 1,971 were unique genes annotated by Ensembl. These genes were then analyzed by Davies-Bouldin index [23] to render optimal number of clusters for partition clustering and were assigned to one of the five trends of temporal gene regulation (Figure 3). The graphs represent 10 most regulated genes in each cluster, and were groups of genes that exhibited: peak-upregulation at day 5 after MIA induction, followed by decrease in gene expression (*Cluster I *); peak-upregulation at day 9 after MIA induction (*Cluster II *); gradual increase in gene expression that peaked at day 21 after MIA injection (*Cluster III *); peak-downregulation at day 5 after MIA injection, followed by relative increase in gene expression (*Cluster IV *); and peak-downregulation at day 9 after MIA induction (*Cluster V *). Validation of at least two genes in each cluster by rt-PCR exhibited similar trends in the differences in gene expression as in microarray analysis (Figure 4). However, rt-PCR technique being more sensitive contributed to greater fold changes in gene expression as compared to the microarray analysis.

**Figure 5:**
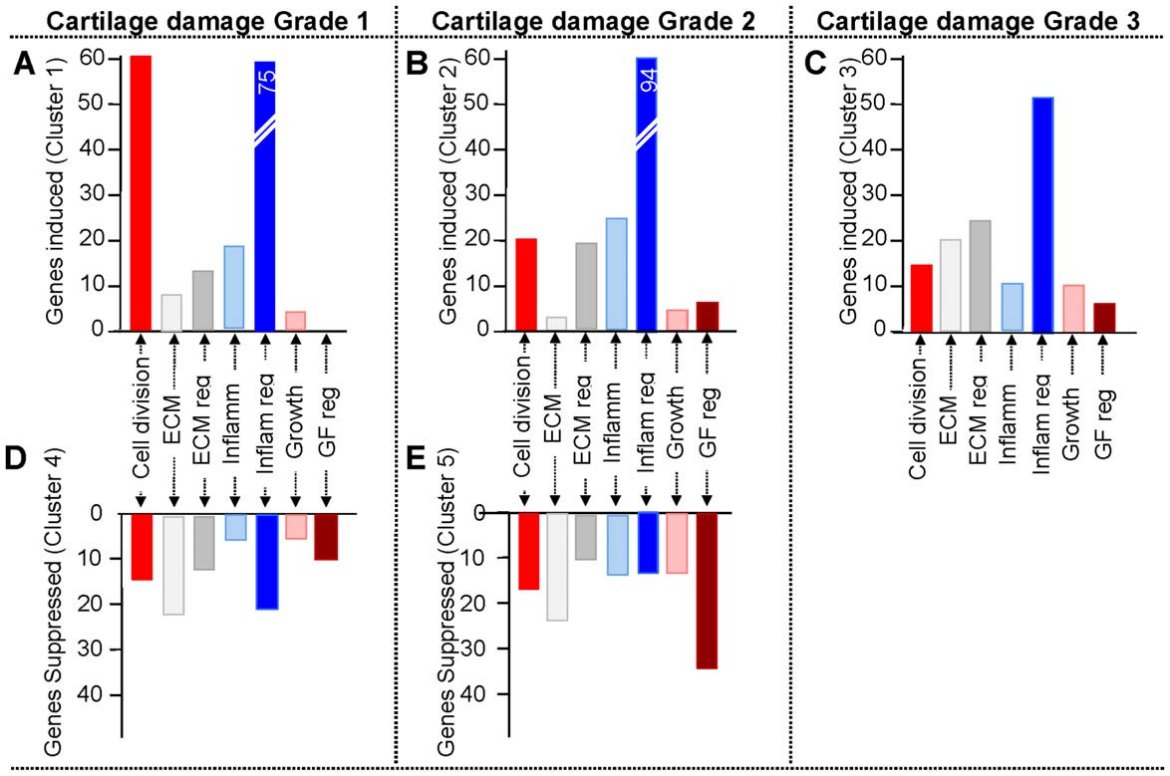
Distribution of genes in each cluster according to their functions. Relative distribution of genes in each cluster subdivided according to their functions. Cell division, genes involved in cell division, proliferation, apoptosis; Growth factors, genes for growth factors and their receptors; GF reg, growth factor regulatory molecules and transcription factors; Inflammation, cytokines, chemokines and their receptors; Inflam reg, inflammatory mediators, signaling molecules, transcription factors, and regulators; ECM, extracellular matrix proteins; ECM reg, Proteases, regulators of ECM synthesis and breakdown; Others genes involved in cell metabolism, transporters and ion channels and genes of unknown function (Tables S1, S2, S3, S4, and S5).

Among the five distinct biologically functional gene clusters, IPA identified three clusters mainly associated with inflammation and immunological disorders (*Clusters I*, *II* and *III *), and the remaining two clusters associated with musculoskeletal function and disorders (*Clusters IV* and *V *) (Figure 3, Table 1). To delineate the overall functional relevance, the genes were further categorized into 7 functional sets: (i) Inflammation (cytokines, chemokines, and their receptors); (ii) Inflammation regulators (mediators, transcription factors, and signaling molecules that regulate inflammation); (iii) Cell division/proliferation; (iv) ECM (molecules of the matrix); (v) ECM regulators (molecules that regulate matrix synthesis and degradation); (vi) Growth factors (growth factors and their receptors); (vii) Growth factor regulators (signaling molecules and transcription factors that regulate growth factors) (Figure 5, Tables 2, 3, 4, 5 and 6). Genes including molecules involved in cell metabolism, transporters and ion channels, and those with unknown functions were not included in the present analysis. The genes in these Tables reflect: genes with known function, the degree of gene regulation, and are in proportion to the group of genes regulated in a particular cluster shown in Figure 5.

**Figure 6:**
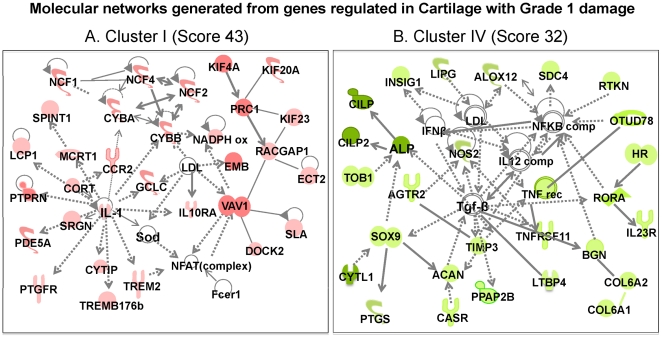
Molecular networks generated from the genes in each cluster by Ingenuity Pathways Analysis. The molecular networks generated from genes in: (A) Cartilage with Grade 1 damage (*Cluster I*) – Immunological disease network, showing upregulation of genes associated with acute/innate immune response; (B) Cartilage with Grade 1 damage (*Clusters IV*) - Skeletal & muscular development and function network, showing downregulation of transcription factors and growth factors associated with matrix synthesis. The symbols under individual mediators are defined in Figure 8B. Red, green, and white colors represent upregulation, downregulation and no regulation as compared to *cont* cartilage, respectively. The shading of each color represents fold change in gene expression; dark, higher changes and light lower changes.

**Table 1:** IPA analysis showing top biological functions of each gene cluster at various stages of cartilage destruction in MIA.

Cartilage damage	*Cluster*	Name	p-value	Genes (#) associated
Grade 1 (MIA5)	**I**	**Inflammation**	**9.12E-09 – 1.80E-03**	**116**
		**Immunological Disease**	**2.55E-09 – 1.80E-03**	**103**
	IV	**Genetic Disorder**	**1.37E-06 – 2.08E-02**	**163**
		**Skeletal and Muscular Development and Function**	**2.10E-07 – 1.73E-02**	**95**
Grade 2–2.5 (MIA9)	II	**Genetic Disorder**	**3.21E-10 – 6.79E-04**	**116**
		**Inflammatory Response**	**4.81E-12 – 1.15E-03**	**96**
	V	**Genetic Disorder**	**1.87E-12 – 2.88E-02**	**235**
		**Skeletal and Muscular Disorders**	**1.31E-10 – 2.88E-02**	**134**
Grade 3–3.5 (MIA21)	III	**Skeletal and Muscular Disorders**	**1.47E-08 – 1.59E-03**	**131**
		**Inflammatory Disease**	**6.71E-08 – 1.81E-03**	**124**

**Table 2:** Induction of salient genes in cartilage with Grade 1 damage (*Cluster I*).

Cluster I (421 annotated genes, 267 genes in IPA database)			Fold change		
Gene	Description and Function	Group	OA5	OA9	OA21
*Diap3*	Diaphanus homolog 3; cytokinesis	CD	4.60	3.36	2.25
*Anln*	Anilin; cytokenesis	CD	4.42	3.37	1.76
*Emb*	Embigin; protein tyrosine phosphatase receptor type N	CD	4.09	4.00	2.00
*Kif4*	Kinesin Family member 4; microtubule-based motor proteins involved in cell division	CD	3.72	2.08	1.16
*Kif23*	Kinesin Family member 23; microtubule-based motor proteins involved in cell division	CD	3.56	2.09	1.30
*Prc1*	Protein regulator of cytokinesis 1; cytokinesis	CD	3.34	2.43	1.67
*Dusp6*	dual specificity phosphatase; mitogenic signal transduction via Erk and Map kinases	CD	3.28	3.41	2.88
Vav1	Vav Guanine nucleotide exchange factor; cell division and differentiation	CD	3.08	2.61	1.69
Ccnb1	Cyclin B1; cell cycle regulation	CD	2.95	2.10	−1.13
Ccne1	Cyclin E1; cell cycle regulation	CD	2.63	1.50	1.02
*Il1rl1*	IL-1 receptor-like 1	Inf	8.60	3.50	2.11
*Tlr7*	Toll-like receptor 7; LPS receptor	Inf	5.06	2.74	3.23
*Irf8*	Interferon regulatory factor 8; regulate growth and differentiation	Inf	2.49	1.04	−1.02
*Ccr2*	Chemokine (C-C motif) receptor; cell migration	Inf	2.30	2.06	1.02
*Il1b*	Interleukin 1 beta; inflammation	Inf	2.22	2.0	1.54
*Il33*	IL-33; neutrophil migration	Inf	2.00	−1.02	1.15
*C3ar1*	complement 3a receptor 1; inflammation regulation	Inf2	4.92	3.18	3.72
*Hla-dmb*	Major histocompat complex, class II DM-b	Inf2	4.65	2.93	2.24
*Ptger4*	Prostaglandin E receptor 4; level and stability of cyclooxygenase-2	Inf2	4.55	2.81	2.43
*Fcgr1a*	Fc fragment of IgG receptor 1a (CD64); immune response	Inf2	4.26	3.03	3.00
*Itgb2*	Integrin beta 2; cell adhesion	Inf2	4.10	4.18	3.43
*Itgal*	Integrin alpha L; cell adhesion	Inf2	3.84	3.41	2.29
*Vcan*	Versican; connects cells to ECM, cell motility/division	ECM	9.09	7.95	6.35
*Fbln2*	Fibulin 2; ECM protein cell differentiation	ECM	6.55	4.12	2.87
*Spon1*	Spondin 1; cell adhesion protein	ECM	2.92	2.26	2.56
*Aspn*	Asporin; negative regulator of TGF-β	ECM2	9.33	5.77	8.38
*Hpse*	Heparanase; cleaves polymeric heparan sulfate molecules	ECM2	6.42	3.60	3.30
*Ctss*	Cathepsin S; activates serine proteases	ECM2	3.91	3.21	3.68
*Arsb*	Arylsulfatase b; hydrolyzes sulfate groups on chondroitin sulfate and dermatan sulfate	ECM2	3.75	3.70	2.62
*Ctsc*	Cathepsin C; cystein proteases/elastase	ECM2	3.72	3.66	3.29
*Plau*	Plasminogen activator, urokinase; serine protease, promotes fibrinolysis	ECM2	2.87	2.79	1.62
*Fgf7*	Fibroblast growth factor 7; growth of keratinocytes	GF	2.89	−1.01	1.14
*Csfrb*	Colony stimulating factor receptor b; hematopiesis, immunological defense, bone metabolism	GF	2.76	2.14	1.54
*Sfrp2*	Secreted Frizzled related protein 2; Wnt signaling	GF2	4.04	−1.06	3.05
*Sfrp1*	Secreted Frizzled related protein 1; Wnt signaling	GF2	3.59	3.07	1.94

**CD**, genes involved in cell division, proliferation, apoptosis; **Inf**, cytokines, chemokines and their receptors; **Inf2**, inflammatory mediators and their receptors, signaling molecules, transcription factors, and regulators; **ECM**, extracellular matrix proteins; **ECM2**, Proteases, regulators of ECM synthesis and breakdown; **GF**, genes for growth factors and their receptors; **GF2**, growth factor signaling molecules, transcription factors. A full list of these genes is given in Table S1.

**Table 3:** Suppression of salient genes in cartilage with Grade 1 damage (*Cluster IV*).

Cluster IV (312 annotated genes, 203 genes in IPA database)			Fold change		
Gene	Description and Function	Group	OA 5	OA 9	OA 21
*Scrg1*	stimulator of chondrogenesis 1; suppresses cell growth but induces chondrogenic differentiation	CD	−61.6	−53.8	−12.2
*Cidea*	cell death-inducing DFFA-like effector a; activates apotptosis	CD	−2.66	−1.76	−2.01
*Cytl1*	cytokine-like 1; likely involved in proteoglycan synthesis	Inf	−53.6	−47.5	−49.8
*Tnfrsf11b*	TNFr superfamily, member 11b (OPG); inhibits osteoclastogenesis	Inf	−9.03	−8.75	−4.40
*Il23r*	interleukin 23 receptor; JAK-STAT signaling	Inf	−2.56	−2.12	−1.46
*Sod3*	superoxide dismutase 3, extracellular; antioxident enzymes	Inf2	−7.41	−3.89	−2.42
*Alox12*	arachidonate 12-lipoxygenase; proliferative, antiapoptotic and proangiogenic	Inf2	−2.53	−2.27	−2.77
*Ptgds*	PGD2 synthase;, smooth muscle contraction/relaxation, inhibition of platelet aggregation	Inf2	−2.09	−2.07	−2.00
*Cilp*	cartilage intermediate layer protein; cartilage matrix protein	ECM	−92.3	−66.2	−23.2
*Cilp2*	cartilage intermediate layer protein 2; cartilage matrix protein	ECM	−21.9	−19.2	−3.31
*Fbln7*	fibulin 7; takes part in cell binding	ECM	−13.3	−9.92	−3.71
*Fmod*	Fibromodulin; ECM assembly	ECM	−13.1	−11.9	−1.75
*Hapln3*	Hyaluronan-proteoglycan link protein 3; proteoglycan link protein 3	ECM	−6.50	−6.13	−6.03
*Col27a1*	collagen, type XXVII, alpha 1; involved in calcification of cartilage	ECM	−4.57	−3.24	−3.30
*Acan*	aggrecan; major cartilage matrix protein	ECM	−4.12	−3.30	−2.05
*Cspg4*	chondroitin sulfate proteoglycan 4; cartilage matrix protein	ECM	−3.07	−2.73	−1.71
*Spon2*	spondin 2; extracellular matrix protein	ECM	−2.89	2.23	1.73
*Col16a1*	collagen, type XVI, alpha 1; cell attachment	ECM	−2.69	−1.53	−1.29
*Eln*	Elastin; part of ECM	ECM	−2.59	1.05	1.51
*Sdc4*	syndecan 4; proteoglycan cell interactions	ECM	−2.27	−1.01	−1.36
*Bgn*	biglycan; cartilage matrix protein	ECM	−2.12	−1.69	1.07
*Col14a1*	collagen, type XIV, alpha 1; fibrillar collagen	ECM	−2.07	−1.19	3.92
*Pi15*	peptidase inhibitor 15; serine protease inhibitor	ECM2	−18.0	−17.9	−4.74
*Chst3*	chondroitin 6 sulfotransferase 3; chondroitin sulfate biosynthesis	ECM2	−10.8	−7.17	−4.65
*Serpina3*	serpin peptidase inhibitor, clade A mem 3; anti-trypsin activity	ECM2	−10.8	−1.85	−1.62
*Chst11*	Chondroitin-4 sulfotransferase 11; chondroitin sulfation	ECM2	−4.71	−4.25	−4.88
*Timp3*	TIMP metallopeptidase inhibitor 3; inactivates MMPs	ECM2	−2.87	1.14	1.31
*Sulf2*	sulfatase 2; sulfation of proteoglycans	ECM2	−2.44	1.06	1.45
*Hs6st2*	heparan sulfate 6-O-sulfotransferase 2; heparan sulfate synthesis	ECM2	−2.07	−1.91	−2.02
*Gdf10*	growth differentiation factor 10; Growth and differentiation	GF	−22.6	−16.6	−4.89
*Igf2*	insulin-like growth factor 2; mitogenic	GF	−7.05	−6.48	−4.87
*Bmp6*	bone morphogenetic protein 6; cartilage and bone formation	GF	−5.96	−5.40	−3.56
*Fgfrl1*	fibroblast growth factor receptor-like 1; cell proliferation	GF	−5.06	−4.63	−3.44
*Spock1*	sparc/osteonectin; inhibits cell-cycle and influences ECM synthesis	GF	−3.01	−2.76	−3.02
*Vegfa*	vascular endothelial growth factor A; angiogenesis	GF	−2.57	−1.13	−1.09
*Crim1*	cys rich transmembrane BMP regulator 1; BMP regulator 1	GF2	−12.7	−10.8	−8.59
*Sox9*	SRY-box 9; transcription factor for chondrogenesis	GF2	−10.2	−9.70	−5.59
*Htra4*	HtrA serine peptidase 4; suppresses IGF and TGF-β signaling	GF2	−10.0	−4.49	2.18
*Igfbp7*	IGF binding protein 7; stimulates PGI2 secretion, and cell adhesion	GF2	−3.19	−2.15	−1.10
*Fzd8*	frizzled homolog 8 (Drosophila); Wnt signaling	GF2	−3.15	−2.15	1.10
*Ltbp4*	latent TGFβ binding protein 4; TGF-β regulation	GF2	−2.86	−1.68	1.22

Please see Table 2 for group description. A full list of these genes is given in Table S2.

**Table 4:** Induction of salient genes in cartilage with Grade 2 damage (*Cluster II*).

Cluster II (430 annotated genes, 305 genes in IPA database)			Fold change		
Gene	Description and Function	Group	OA 5	OA 9	OA 21
*Dapk1*	death-associated protein kinase 1; programmed cell death	CD	2.95	4.06	3.13
*Ccng1*	cyclin G1; cell cycle	CD	1.84	2.23	1.67
*Ccl9*	chemokine (C-C motif) ligand 9; bone resorption	inf	4.65	14.7	5.41
*Ccr1*	chemokine (C-C motif) receptor 1; immune cell recruitment	inf	4.53	10.7	5.43
*Tnfsf11*	TNF superfamily, memb 11 (RANKL); bone resorption	inf	1.98	9.52	6.04
*Ccl7*	chemokine (C-C motif) ligand 7; immune cell recruitment	inf	4.08	6.88	2.30
*Lif*	leukemia inhibitory factor; acute phase protein synthesis	inf	3.06	5.87	2.84
*Ccl2*	chemokine (C-C motif) ligand 2; chemoattraction migration of cells	inf	3.09	4.89	3.21
*Il18*	interleukin 18 (IFNg-inducing factor); macrophage activation	inf	3.63	4.23	3.69
*Tnfrsf11a*	TNF receptor mem 11a; NFκB activator, osteoclastogenesis	inf	3.01	3.73	2.71
*Ccr5*	chemokine (C-C motif) receptor 5; migration of immune cells	inf	3.27	3.59	2.60
*Tnfrsf1b*	TNF rec fam mem1B; ligand for OPG/RANKL, osteoclastogenesis	inf	2.81	3.30	2.37
*Il7*	Interleukin-7; amplification of immune response	inf	1.9	2.35	2.00
*Ifngr2*	Interfero receptor 2; amplification of immune response	inf	1.8	2.25	1.71
*Tfpi2*	tissue factor pathway inhibitor 2; inhibits blood coagulation	inf2	5.68	8.02	3.27
*Pik3cb*	phosphoinositide-3-kinase, b-polypep; immune cell activation	inf2	4.36	7.04	5.29
*Dusp4*	dual specificity phosphatase 4; negative regulator of cell prolif.	inf2	3.43	6.99	3.27
*Itgam*	integrin, alpha M (C3 receptor 3); C3 receptor 3	inf2	5.47	5.92	3.82
*Itgax*	integrin αX (C 3 receptor 4 subunit); C3 receptor4	inf2	3.45	5.68	3.22
*Ptpre*	Protein tyrosine phosphatase, receptor E; cell growth and different.	inf2	2.39	5.59	2.80
*Ptpn22*	Protein tyr phosphate; T cell regulation	Inf2	2.51	4.07	2.91
*Cx3cr1*	chemokine (C-X3-C motif) recept ‘1; immune cell regulation	inf2	2.85	3.84	2.92
*Tank*	TRAF family member,;NFκB activator	inf2	2.50	3.79	3.03
*Cxcl16*	chemokine (C-X-C motif) ligand 16; immune response	inf2	3.15	3.69	3.01
*Ripk3*	receptor-interact ser-thr kinase 3; NFκB signaling	inf2	3.33	3.58	2.51
*Itga2*	Integrin α2; receptor for collagens, fibronectin and cadherin	inf2	1.74	3.19	2.41
*Nfkbie*	NFκB polypeptide ε; negative regulator of NFκB signaling	inf2	1.83	2.64	1.87
*Map2k3*	Map Kinase kinase kinase; MAP kinase signaling	Inf2	2.00	2.64	2.00
*Nfkb2*	NFκB polypeptid 2 (p49/p100); NFκB signaling	inf2	1.32	2.40	1.60
*Col5a3*	collagen, type V, alpha 3; fibrillar collagen	ECM	4.42	8.40	5.25
*Sdc1*	syndecan 1; heparan sulfate proteoglycan	ECM	3.54	5.79	3.19
*Mmp12*	matrix metallopeptidase 12; elastase	ECM2	13.6	33.4	18.2
*Mmp19*	matrix metallopeptidase 19; degrades aggrecan and COMP	ECM2	5.03	12.9	8.00
*Adamts4*	ADAMTS 4; degrades proteoglycans	ECM2	2.37	6.21	4.31
*Timp1*	TIMP metallopeptidase inhibitor 1; known to inhibit MMPs	ECM2	3.58	4.53	3.04
*Adamts12*	ADAMTS 12; degrades COMP and aggrecan	ECM2	3.14	4.24	3.64
*Hyal1*	hyaluronoglucosaminidase 1; cleaves hyaluronoglucosamines	ECM2	2.24	3.66	2.37
*Arsb*	arylsulfatase B; degrades glycosaminoglycan	ECM2	2.82	3.57	2.44
*Adamts7*	ADAM metallopeptidase; degrades COMP	ECM2	2.46	3.22	2.86
*Mmp9*	matrix metallopeptidase 9; cleaves Collagen IV and V, fibronectin	ECM2	1.42	3.16	2.50
*Adam8*	ADAM metallopeptidase domain 8; may cleave extracellular matrix	ECM2	1.95	2.45	1.35
*Hyal3*	hyaluronoglucosaminidase 3; hyaluronidase	ECM2	1.38	2.23	1.55
*Serpine2*	serpin peptidase inhibitor, clade E2; inhibits thrombin, trypsin and urokinase	ECM2	1.52	2.01	1.55
*Pdgfb*	PDGFβ polypeptide; chondrogenesis	GF	2.15	4.11	2.92
*Csf1r*	CSF 1 receptor; for CSF and IL-34, macrophage differentiation	GF	2.94	4.09	2.94
*Tgfbr1*	TGF β receptor 1; activates SMAD signaling for bone formation	GF	2.45	3.48	2.56
*Sfrp4*	secreted frizzled-related protein 4; Wnt signaling	GF2	5.50	8.96	6.26
*Wnt5a*	Wnt family, member 5A; Wnt signaling	GF2	3.65	8.15	6.47
*Inhba*	inhibin, beta A; TGF-β signaling	GF2	4.43	7.00	4.56
*Igfbp4*	IGF binding protein 4; IGF regulation	GF2	4.15	5.34	4.23
*Wnt7b*	Wnt family, member 7B; Wnt signaling	GF2	1.76	2.77	1.38
*Igfbp3*	IGF binding protein 3; IGF regulation	GF2	2.33	2.44	2.16

Please see Table 2 for group description. A full list of these genes is given in Table S3.

**Table 5:** Suppression of salient genes in cartilage with Grade 2 damage (*Cluster V*).

Cluster V (417 annotated genes, 274 genes in IPA database)			Fold change		
Gene	Description and Function	Group	OA 5	OA 9	OA 21
*Cdkn1c*	CDK inhibitor 1C (p57); negative regulator of cell proliferation	CD	−4.94	−5.79	−3.44
*Pdcd4*	programmed cell death 4; inhibits proliferation	CD	−2.19	−2.90	−1.74
*Il7*	interleukin 7; B and T cell development	Inf	−4.40	−5.85	−4.53
*Il16*	IL 16; chemoattarctant for immune cells	inf	−3.78	−4.52	−1.68
*Il17b*	interleukin 17B; Induces TNF-a and IL-1b from monocytic cells	inf	−2.84	−3.32	−2.93
*Nrk*	Nik related kinase; NF-kB sgnaling	inf2	−16.4	−18.7	−16.2
*Matn3*	matrilin 3; development and homeostasis of cartilage and bone	ECM	−26.3	−98.1	−74.6
*Col10a1*	collagen, type X, alpha 1; matrix in hypertrophic cartilage	ECM	−29.0	−83.3	−45.5
*Col9a1*	collagen, type IX, alpha 1; major cartilage matrix protein	ECM	−15.3	−77.0	−46.1
*Col2a1*	collagen, type II, alpha 1; major cartilage matrix protein	ECM	−14.9	−50.3	−9.06
*Chad*	Chondroadherin; chondro & osteoblasts integrin α2β1	ECM	−18.3	−32.6	−7.10
*Col9a2*	collagen, type IX, alpha 2; major cartilage matrix protein	ECM	−13.5	−27.6	−22.4
*Scin*	scinderin; cellular protein involved in exocytosis	ECM	−22.2	−24.4	−11.7
*Hapln1*	hyaluronan and proteoglycan link prot 1; binds to aggregates of proteoglycan monomers with hyaluronic acid	ECM	−11.0	−19.0	−7.54
*Col9a3*	collagen, type IX, alpha 3; major cartilage matrix protein	ECM	−9.38	−18.9	−12.6
*Col11a2*	collagen, type XI, alpha 2; major cartilage matrix protein	ECM	−6.29	−16.9	−9.94
*Vit*	vitrin; promotes matrix assembly and cell adhesiveness	ECM	−13.4	−15.1	−10.9
*Prg4*	proteoglycan 4; major cartilage matrix protein	ECM	−7.61	−7.65	−3.52
*Col11a1*	collagen, type XI, alpha 1; major cartilage matrix protein	ECM	−3.77	−5.71	−2.98
*Mgp*	matrix Gla protein; associated with cartilage and bone matrix	ECM	−4.72	−5.63	−3.28
*Matn1*	matrilin 1; major cartilage matrix protein	ECM	−3.86	−4.00	−3.97
*Fbln5*	fibulin 5; promote adhesion of endothelial cells	ECM	−2.54	−3.08	−1.66
*Col24a1*	collagen, type XXIV, alpha 1; regulate Col type I	ECM	−1.97	−2.51	−1.51
*Col5a3*	collagen, type V, alpha 3; fibrillar collagen	ECM	−1.82	−2.34	−1.79
*Hs3st1*	heparan sulfate 3-O-sulfotransferase 1;heparin sulfate synthesis	ECM 2	−6.09	−6.25	−7.69
*Adamts3*	ADAMTS 3; cleavage of propeptide of type II collagen	ECM 2	−3.32	−3.91	−4.69
*Adamts6*	ADAMTS 6; likely a metallopeptidase	ECM 2	−2.15	−3.05	−2.36
*Alpl*	alkaline phosphatase; matrix mineralization	ECM 2	−2.80	−2.83	−1.26
*Chsy3*	chondroitin sulfate synthase 3; chondroitin synthesis	ECM 2	−2.58	−2.61	−2.06
*Has2*	hyaluronan synthase 2; hyaluran synthesis	ECM 2	−1.66	−2.29	−3.28
*Mmp16*	matrix metallopeptidase 16; membrane type MMP-3	ECM 2	−1.38	−2.20	−1.13
*Omd*	osteomodulin; likely role in mineralization	GF	−11.5	−13.0	−3.35
*Fgfr3*	fibroblast growth factor receptor 3; cell growth in wound healing	GF	−5.67	−7.94	−5.77
*Fgfr2*	fibroblast growth factor receptor 2; cell growth in wound healing	GF	−2.77	−5.61	−3.06
*Ctgf*	connective tissue growth factor; chondrocyte prolifer and differentia	GF	−2.79	−5.24	−1.68
*Bmp5*	bone morphogenetic protein 5; cartilage and bone formation	GF	−2.50	−4.75	−2.60
*Ghr*	growth hormone receptor; bone growth	GF	−3.19	−4.24	−2.37
*Tgfbr3*	transforming growth factor, b receptor III; Smad activation	GF	−2.32	−3.55	−1.74
*Bmp3*	BMP 3; antagonizes BMPs in bone formation	GF	−1.12	−3.34	−1.36
*Igfbp5*	insulin-like growth factor binding prot 5; promote growth by IGF	GF	−2.29	−3.04	−1.33
*Vdr*	vitamin D3 recp; Ca++ homeostasis	GF	−1.21	−2.72	−2.78
*Tgfb3*	TGFb3; chondrocyte div and differentiation	GF	−1.75	−2.65	−1.25
*Bmpr1a*	BMP receptor, type IA; Smad transcriptional activation	GF	−2.01	−2.46	−1.53
*Egf*	epidermal growth factor (b-urogastrone); mitogenic involved in cell	GF	−2.11	−2.37	−2.04
*Frzb*	frizzled-related protein; cartilage and bone development	GF2	−47.4	−52.7	−49.6
*Ptch1*	patched homolog 1; receptor for Ihh	GF2	−9.85	−16.1	−7.60
*Wif1*	WNT inhibitory factor 1; inhibits Wnt proteins	GF2	−13.0	−15.2	−6.43
*Sfrp5*	secreted frizzled-related protein 5; Wnts regulation, growth	GF2	−9.76	−11.8	−11.4
*Fzd9*	frizzled homolog 9; receptor for Wnts regulates βcatenin pathway	GF2	−8.61	−8.90	−6.65
*Sox6*	SRY (sex determining region Y)-box 6; role in skeleton formation	GF2	−3.91	−5.87	−3.02
*Ihh*	Indian hedgehog; endochondral ossification bone growth ossificatic	GF2	−4.63	−5.24	−4.05
*Wisp3*	WNT1 inducible signal protein 3; Wnt regulation growth & different	GF2	−3.69	−3.91	−3.73
*Dlx5*	distal-less homeobox 5; chondrogenesis and osteoblastogenesis	GF2	−2.52	−3.73	−2.46

Please see Table 2 for group description. A full list of these genes is given in Table S4.

**Table 6:** Induction of salient genes in cartilage with Grade 3–3.5 damage (*Cluster III*).

Cluster III (391 annotated genes, 271 genes in IPA database)			Fold change		
Gene	Description and Function	Group	OA 5	OA 9	OA 21
*Ccnd1*	cyclin D1; cell cycle control	CD	2.28	2.22	2.72
*Cdkn1a*	cyclin-dependent kinase inhibitor 1A; inhibits cell division	CD	1.10	1.95	2.56
*Pawr*	PRKC, apoptosis, WT1, regulator; proapoptotic	CD	1.71	1.98	2.44
*Klf10*	Kruppel-like factor 10; transcriptional repressor of cell growth	CD	1.36	2.60	2.42
*Cxcl13*	chemokine (C-X-C motif) ligand 13; chemotactic for B-lymphocytes	Inf	1.64	2.10	2.98
*Il10rb*	interleukin 10 receptor, beta; anti-inflamamtory	Inf	2.14	2.40	2.81
*Il15*	interleukin 15; proliferation of T-lymphocytes	Inf	1.26	1.48	2.01
*Cdh13*	cadherin 13, H-cadherin (heart); cell-cell adhesion glycoprotein	Inf2	1.46	4.63	8.59
*Lilbr4*	leukocyte IgG-like receptor, subfam B, mem4; controls inflammation	Inf2	7.33	6.00	7.00
*Lbp*	LPS binding protein; binds LPS to present it to TLR4 and CD14	Inf2	1.66	3.65	4.45
*F3*	coagulation factor III; initiates coagulation cascade with Factor VII	Inf2	2.05	2.56	3.14
*Lpl*	lipoprotein lipase; cleaves triglycerides	Inf2	1.36	1.58	2.91
*Alox5*	arachidonate 5-lipoxygenase; catalyzes leukotriene synthesis	Inf2	1.82	1.61	2.86
*Tlr4*	toll-like receptor 4; LPS receptor	Inf2	2.14	1.83	2.85
*Vcam1*	vascular cell adhesion molecule 1; immune response	Inf2	2.69	1.82	2.61
*Ptges*	prostaglandin E synthase; prostaglandin synthesis, inflammatory responses, pain perception	Inf2	1.40	2.34	2.60
*Pld2*	phospholipase D2; cleaves phosphatidyl choline	Inf2	1.72	2.32	2.49
*Socs3*	suppressor of cytokine signaling 3; negative regulator of inflammatory response	Inf2	1.69	2.09	2.47
*Nfkbia*	NF-κB inhibitor, alpha; inhibitor of NF-kB- IkBa	Inf2	1.42	2.45	2.17
*Tnn*	tenascin N; cartilage and bone formation	ECM	15.5	18.8	20.9
*Postn*	periostin; osteoblast specific factor; cell adhesion, mineralization	ECM	5.88	5.05	7.23
*Lum*	lumican; collagen fibril organization	ECM	4.09	5.03	5.90
*Col18a1*	collagen type XVIII a1; a potent antiangiogenic	ECM	2.71	3.92	5.66
*Col4a1*	collagen type IV a1; inhibits endothelial proliferation/angiogenesis	ECM	1.80	3.03	4.33
*Col3a1*	collagen type III a1; soft tissue associated with Collagen type 1	ECM	2.02	3.19	3.88
*Col12a1*	collagen type XII, a1; fibrillar collagen	ECM	2.10	3.11	3.42
*Col4a2*	collagen type IV a2; inhibits endothelial proliferation/angiogenesis	ECM	1.39	2.12	3.13
*Col6a3*	collagen, type VI, alpha 3; linkage of matrix/cell	ECM	1.30	2.42	2.71
*Col5a1*	collagen, type V, alpha 1; fibrillar collagen	ECM	1.10	1.73	2.12
*Adam23*	ADAM metallopeptidase domain 23; nonproteolytic metalloprotease, cell-cell adhesion	ECM2	3.97	3.56	5.50
*Serpine1*	serpin peptidase inhibitor, clade E1; inhibits plasminogen activator	ECM2	3.27	3.88	4.81
*Timp2*	TIMP metallopeptidase inhibitor 2; inhibitor of several MMPs	ECM2	1.38	2.19	3.07
*Mmp14*	matrix metallopeptidase 14; activates progelatinase	ECM2	1.95	3.29	3.01
*Mmp2*	MMP 2; ECM breakdown in normal physiologic processes	ECM2	1.01	1.99	2.84
*Mmp11*	matrix metallopeptidase 11; matrix remodeling, vascular invasion	ECM2	1.11	1.47	2.39
*Adamts2*	ADAMTS 2; cleaves tissue propeptides of collagen type I and II	ECM2	1.28	1.62	2.37
*Ctsd*	cathepsin D; intracellular proteinase inhibitor	ECM2	1.61	2.3	2.25
*Tgfb2*	TGF beta 2; cell division and growth differentiation	GF	1.26	2.67	2.63
*Pdgfrb*	PDGF receptor, β polypeptide; angiogenesis, cell proliferation and differentiation	GF	1.18	1.77	2.46
*Osmr*	oncostatin M receptor; increases cartilage degradation	GF	1.85	2.41	2.45
*Pdgfc*	PDGF C; wound healing, proliferation and remodeling	GF	1.04	2.22	2.15
*Ogn*	osteoglycin; Induces bone formation with TGF-beta-1 or TGF-beta-2	GF	1.22	1.19	2.06
*Egfr*	epidermal growth factor receptor; cell growth/differentiation	GF	1.29	1.17	2.05
*Wisp2*	WNT1 inducible signaling protein 2; bone turnover	GF2	2.65	5.71	6.08

Please see Table 2 for group description. A full list of these genes is given in Table S5.

### Cartilage with Grade 1 damage (MIA5) exhibits gene expression associated with innate immunity and cell proliferation

The cartilage with Grade 1 damage showed upregulation of genes in *Cluster I*, and downregulation in *Cluster IV*. According to IPA, the genes in *Cluster I* were functionally associated with inflammation (116 genes; *p*-value 9.12E-09 – 1.80E-03) and immunological diseases (103 genes; *p*-value 2.55E-09 – 1.80E-03) (Table 1). The inflammation associated cytokine, chemokines and their receptors significantly upregulated were *Il1b*, *IL1rl1*, *Tlr7*, *Ccr2*, and *Il-33*. The major inflammation regulatory upregulated genes were, *C3ar1*, *Itgb2*, -*a2*, -*a4*, *Ptger4*, various IgG Fc receptors (*Fcrls*, *Fcgr1a*, *Fcgr2a*, *Fcgr2b*), molecules of the major histocompatibility complex (*Hla-dmb*, *H2-Ea*, *cd74*, *Hla-dma*, *Rt-1ba*) and transcription factors *Irf5*, *Irf8* (Table 2, Table S1) [24].

Interestingly, the genes associated with cell cycle/division/differentiation such as *Diap3*, *Anln*, *Prc1*, *Emb*, *Kif4*, *Kif23*, *Dusp6*, *Vav1*, *Ccnb1*, *Ccna2*, *Ccnb2*, *Ccne1*, *Ccnf*, and *Cdk6* were also highly upregulated (Table 2, Figure 5A, Table S1). The expression of these genes paralleled the chondrocyte proliferation characteristically observed as disoriented clusters of chondrocyte distributed in the cartilage (Figure 1g).

Despite the presence of cytokines like IL-1β and IL-33, genes for several ECM proteins involved in cell-matrix attachment were significantly upregulated in Grade 1 cartilage damage. These genes included *Vcan*, *Fbln2*, and *Spon1*. Additionally, proteinases with broad specificity involved in protein/matrix breakdown were upregulated such as *Hpse*, *Ctsc*, *Ctss*, *Arsb*, and *Plau* (Table 2).

Strikingly, asporin, a suppressor of TGF-β/receptor interactions was more than 9 fold upregulated in *Cluster I* [25]. Additionally, genes for growth factors involved in cell division or immune response such as, *Fgf7*, *Csfrb*, the regulators of Wnt signaling *Sfrp1* and *Sfrp2*, were dynamically upregulated in cartilage with Grade 1 damage.

### Cartilage with Grade 1 damage (MIA5) exhibits suppression of genes associated with matrix synthesis (*Cluster IV*)

In parallel to marked upregulation of genes in cartilage with Grade 1 damage (*MIA5*, *Cluster I *), several genes were significantly downregulated and were assigned to *Cluster IV*. These genes were associated with genetic disorders (163 genes, *p*-value 1.37E-06 – 2.08E-02) and musculoskeletal development and function (95 genes, *p*-value 2.10E-07 – 1.73E-02), and consisted of relatively higher proportion of the genes for extracellular matrix and their regulators (Figures 3D & 5D, Table 3, Table S2). Interestingly, along with genes that induce cell division (*Cluster I *), genes associated with suppression of cell growth and apoptosis were downregulated such as *Scrg1* and *Cidea* in this cluster. Among cytokines, *Cytl1* [26], *IL23r*, and the inhibitor of osteoclastogenesis *Tnfrsf11b* (osteoprotegerin), were major molecules suppressed, along with several proinflammatory mediators *Sod3*, *Alox12*, and *Ptgds*.

More importantly, a significant number of genes responsible for proteoglycan synthesis and assembly were dramatically suppressed. These genes included *Cilp* (−92 fold) and *Cilp2* (−22 fold), *Fbln7*, *Fmod*, *Hapln3*, *Sdc4*, *Flnb*, *Chst3*, *Chst11*, *Acan*, *Cspg4*, *Bgn*, *Spon2*, *Slf2*, *Hs6st2*, and *Eln*. Surprisingly, at Grade 1 cartilage damage, only collagens suppressed were *Col27a1* and *Col16a1* involved in calcification of cartilage and cell attachment, respectively. In parallel, ECM regulatory genes revealed a significant suppression of peptidase inhibitors and anabolic enzymes such as *Pi15*, *Serpina3a*, and *Timp3*, likely accelerating cartilage damage.

The scrutiny of global gene expression in cartilage with Grade 1 damage, also showed that several growth factors required for cartilage growth/homeostasis were dramatically downregulated, such as *Gdf10*, *Ig f2*, *Ig fbp7*, *Bmp6*, *Fg frl1*, *Spock1*, and *Veg fa*. Among growth factor regulatory proteins the most suppressed genes were *Crim1*, *Sox9*, *Ltbp4*, and *htra*, which may cumulatively retard cartilage repair.

### Major genes upregulated in cartilage with Grade 2 damage were associated with chronic inflammation

The Grade 2 cartilage damage showed upregulation of genes in *Cluster II*, belonging to family of genes prevalent in genetic disorders (116 genes, *p*- value 3.21E-10 – 6.79E-04) and inflammatory response and immune trafficking (96 genes, *p*-value 4.81E-12 – 1.15E-03) (Table 1).

As compared to Grade 1, significantly fewer genes associated with cell cycle/division were upregulated in Grade 2 cartilage damage (Figures 3B & 5B, Table 4, Table S3). In fact, a number of genes involved in the inhibition of cell division, *Dapk1* and *Ccng1*, were upregulated. The majority of genes significantly upregulated in Grade 2 (*Cluster II*) were associated with chronic inflammation such as chemokines and their receptor *Ccl2*, *Ccl7*, *Ccl9*, *Ccr1*, *Ccr5*, *Cx3cr1*, and *Cxcl16* as well as cytokines involved in amplification of immune response *Lif*, *Il7*, *Il18*, and *Ifngr2*. More notably, cytokines that induce bone resorption such as *Tnfsf11 (RANKL)*, *Tnfrsf11a*, *Tnfrsf1B* were significantly upregulated explaining the initiation of bone damage observed in µ-CT images (Figure 1l). In parallel, genes involved in the regulation of inflammation were upregulated such as those associated with clotting cascade, *Tfpi2*, adherence, *Itgam*, *Itgax*, *Itga2*, and NF-κB signaling cascades *Tank*, *Ripk2*, *NFkB2*, *NFkbie*, *Map2k3*, and enzymes necessary for the regulation of inflammation *Pik3cb*, *Dusp4*, *Ptpre*, and *Ptpn22*.

Interestingly, the expression of genes for two matrix proteins, *Col5a3* and *Sdc1* was significantly upregulated. Besides these, the expression of genes associated with cartilage matrix degradation was prevalent in the cartilage with Grade 2 damage (*MIA9*). These genes were matrix metallopeptidase (MMP)-9, *Mmp12*, *Mmp19*, *Adamts4*, *Adamts7*, *Adamts12*, *Hyal1*, *Hyal3*, *Arsb*, and *Adam8*. Simultaneously, genes for inhibitors of proteases such as *Timp1* and *Serpine2* were also upregulated.

The major growth factors/receptors upregulated in cartilage with Grade 2 damage were *Pdgfb*, *Csfr1*, and *Igfbp3*, *Igfbp4*, *TGFβr1* and *inhba*. Additionally, several mediators of Wnt and Notch signaling involved in bone formation were upregulated including, *Sfrp4*, *Wnt5a*, and *Wnt7b*. (Table 4, Table S3) [24].

### Major genes suppressed in the cartilage with Grade 2 damage were ECM and growth factor associated genes

During the progression of cartilage damage, we also observed that a significant number of genes were downregulated in the cartilage with Grade 2 damage (*MIA9*, *Cluster V*). Genes in *Cluster V*, in parallel to *Cluster II*, were mainly matrix associated and demonstrated maximal suppression on day 9, and associated with genetic disorders (235 genes, *p*- value 1.87E-12 – 2.88E-02) and skeletal and muscular disorders (134 genes, *p*-value 1.31E-10 – 2.88E-02) (Figures 3E &5E, Table 1).

There were several proinflammatory genes suppressed including IL-7, IL-16 and IL-17b involved in amplification of immune response, and *Nrk* in NF-κB signaling cascade (Table 5). Nevertheless, the most dramatically suppressed gene was matrilin 3, a major component of ECM, involved in the formation of filamentous networks [27]. The expression of several collagens integral to cartilage matrix such as collagens type -IXα1, -IIα1, -IXα2, -IXα3, -XIα1, -XXIVα1, and -Vα3 were significantly downregulated. The expression of other cartilage matrix components involved in cell-matrix and matrix-matrix adhesion were suppressed such as *Chad*, *Scin*, *Hapln1*, *Vit*, *Mgp*, and *Fbln5* (Table 5, Table S4) [24]. Additionally, gene expression of several molecules involved in collagen, chondroitin and hyaluran synthesis were suppressed (*Adamts3*, *Adamts6*, *Chsy3*, *Has2*) as well as those involved in mineralization (*Alpl *).

The growth factors and their receptors and regulatory genes suppressed in the cartilage with Grade 2 damage (*MIA9*) were *Omd*, *Ctg f*, *Bmp5*, *Bmp3*, *Tg fb3*, *Eg f*, *Ig fbp5*, *Fg fr2*, *Fg fr3*, *Ghr*, *Tg fbr3*, *Bmpr1a*, and *Vdr*. Strikingly, frizzeled related protein (*Frzb*) was 52.7 fold suppressed along with other Wnt signaling molecules (*Wif1*, *Sfpr5*, *Fzd9*, and *Wisp3*), and those involved in cartilage/bone development (*Ptch1*, *Ihh*, *Sox6*, and *Dlx5*) (Table 5, Table S4) [24].

### Major genes upregulated in cartilage with Grade 3–3.5 damage were associated with immune adaptation and matrix modeling/remodeling

According to IPA, the specimens exhibiting Grade 3–3.5 cartilage damage (*MIA21*, *Cluster III*), were associated with those generally found in skeletal and muscular disorders (131 genes, *p*-value 1.47E-08 – 1.59E-03) and inflammatory diseases (124 genes, *p*-value 6.71E-08 – 1.81E-03) and showed sustained and successive upregulation in cartilage from Grade 1 to Grade 3–3.5 damage (Figures 3C & 5C, Table 1). Interestingly, at this stage genes that regulate apoptosis or inhibit cell division were upregulated, such as *Cdkn1a*, *Ccnd1*, *Pawr*, *Bcl2l11*, and *Klf10* (Table 6, Table S5).

Among the inflammatory genes, those involved in the regulation of T and B cell functions *Cxcl13*, *IL15*, and suppression of inflammation such as *Il10rb*, *Lilbr4*, *Nfkbia*, *Socs3*, and *Pld2* were simultaneously upregulated. Additionally, genes involved in LPS responses and acute inflammation such as *Tlr4*, *Lbp*, *F3*, *Alox5*, *Lpl*, and *Ptges* were upregulated.

In cartilage with Grade 3–3.5 damage, many ECM proteins involved in bone repair/remodeling were upregulated such as *Tnn*, *Postn*, and *Lum*, and collagens frequently associated with soft tissue and wound repair (collagens type XVIIIα1, -IVα1, -IVα2, -IIIα1, -XIIα1, -Vα1, -VIα3), exhibited increased expression. Additionally, *Adam23*, *Serpine1*, *Timp2*, *Mmp14*, *Mmp11*, *Mmp2*, *Ctsd*, and *Adamts2* involved in ECM regulation during wound repair were also significantly upregulated. The growth factors and their regulators upregulated were *Tgfb2*, *Ogn*, *Pdgfrb*, and *Egfr* that are all involved in cell growth and differentiation. Additionally, signaling molecules associated with Wnt signaling were upregulated such as *Wisp2*, *Notch3*, and *Acvrl*.

Interestingly, we also observed that several of the ECM associated proteins that were suppressed in Grade 2 cartilage damage, were relatively upregulated in cartilage with Grade 3–3.5 damage. For example, *Col2a1*, 50.3-fold downregulated in cartilage with Grade 2 damage, was only 9.06-fold suppressed in Grade 3–3.5 damage. Similar upregulation of ECM associated genes in Grade 3–3.5 damage relative to lesser damaged cartilage included *Matn3*, *Col10a1*, *Col9a1*, *Col9a3*, *Col11a2*, *Col11a3*, *Col5a3*, *Scin*, *Sdc1*, *Hapln1*, *Vit*, *Prg4*, *Matn1*, and *Fbln5*. The expression of a number of growth factors and signaling molecules markedly suppressed in Grade 2 damage were also relatively upregulated in cartilage with Grade 3–3.5 damage. These growth factors were associated with both cartilage and bone damage such as *Omd*, *Fgfr2*, *Fgfr3*, *Ctgf*, *Bmp5*, *Bmp3*, *Igfbp5*, *Egf*, *Frzb*, *Ptch1*. *Wif1*, *Sfrp5*, *Sox6*, *Wisp3*, and *Dlx*.

### Major molecular networks involved in cartilage damage during the progression of MIA

**Figure 7:**
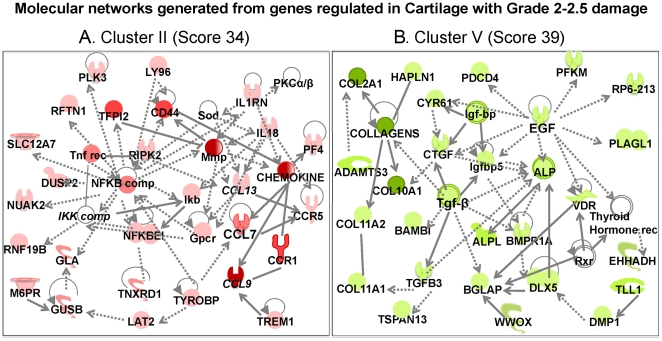
Molecular networks generated from the genes in each cluster by Ingenuity Pathways Analysis. The molecular networks generated from genes in: (A) Cartilage with Grade 2 damage (*Cluster II*) – Inflammatory response/Immune cell trafficking network, showing upregulation of genes associated with chronic inflammation and immune cell trafficking; (B) Cartilage with Grade 2 damage (*Clusters V*) – Skeletal and muscular disease network showing suppression of genes for growth factors and major matrix proteins. The symbols under individual mediators are defined in Figure 8B. Red, green, and white colors represent upregulation, downregulation and no regulation as compared to *cont* cartilage, respectively. The shading of each color represents fold change in gene expression; dark, higher changes and light lower changes.

**Figure 8:**
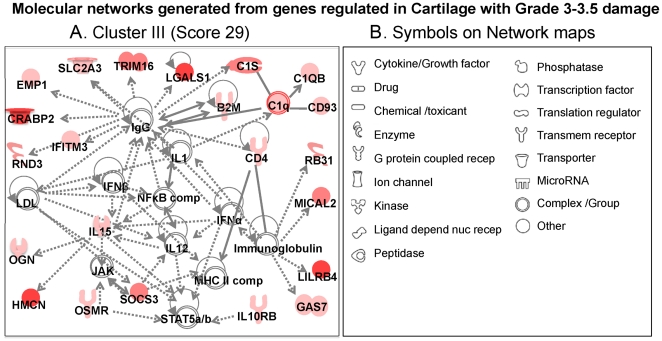
Molecular networks generated from the genes in each cluster by Ingenuity Pathways Analysis. The molecular networks generated from genes in: (A) Cartilage with Grade 3–3.5 damage (*Cluster III*) - Inflammatory disease network showing upregulation of many genes involved in immune suppression and adaptation. Each cluster is based on the genes that were significantly up or downregulated (*p*<0.05, over ±2-fold change) in articular cartilage from *Cont*, *MIA5*, *MIA9*, and *MIA21* specimens. The symbols under individual mediators are defined in (B). Red, green, and white colors represent upregulation, downregulation and no regulation as compared to cont cartilage, respectively. The shading of each color represents fold change in gene expression; dark, higher changes and light lower changes.

We next subjected genes in individual clusters to IPA to generate major functional molecular networks (Figures 6, 7 and 8). The significance and specificity of IPA-generated networks were based on the score of each network. The high score numbers signified that gene networks are extremely specific to each cluster. For example, a score of 43 of the molecular network in *Cluster I* (Figure 6A) indicates that there is only a 1 in 10^43^ chance of getting a network containing the same member of Network Eligible molecules, when same numbers of molecules are randomly picked from the IPA knowledge base.

The molecular network maximally upregulated in the specimens with Grade 1 cartilage damage (*MIA5*) were (i) acute inflammation and (ii) cell cycle/cell division-related genes in *Cluster I*. Genes that typically regulate innate immunity directly or via activation of other mediators formed this network. For example, IL-1β, which auto-regulates its own expression, may also upregulate expression of *Ccr2*, *Trem2* (stimulates production of cytokines and chemokines in macrophages), *IL10ra* (receptor of IL-10), *Ptgfr*, *Cyba* and *Cybb* (phagocytic oxidases that generate superoxide), and *NCF1* and -*2* (oxidases that produce superoxides) (Figure 6A). Strikingly, the genes associated with cell cycle including *Vav1*, *Emb*, *Prc1*, *Kif4A*, *Kif23*, *Kif20A*, and *Dock2* were also prevalent in this network despite the presence of inflammation (Figure S1).

Interestingly, in parallel to upregulation of genes associated with innate immunity and cell cycle in *Cluster I*, other pathways were simultaneously suppressed as observed in the major molecular network for the *Cluster IV* (score 32, Figure 6B). For example, asporin, an inhibitor of TGF-β [25] and a member of *Cluster I*, was considerably upregulated at this stage of cartilage damage, and may be responsible for preventing activation of TGF-β complex, consequently downregulating matrix proteins and growth factors such as *Sox9*, alkaline phosphatase, aggrecan, *Cilp*, *Cilp2*, and other proteoglycans/collegens, directly or via activating intermediary molecules in *Cluster IV* (Figure 6B).

The IPA of genes upregulated in cartilage with Grade 2 damage, revealed a molecular network (score 34) involved in chronic inflammation, immune cell trafficking and perpetuation of inflammatory response (*Cluster II*, Figure 7A). This network appeared to be activated by TNF receptor and may invoke the activities of the NF-κB signaling cascade, RIPK2, a potent activator of NF-κB and inducer of apoptosis and chemokines. The activation of NF-κB complex in turn may play a central role in upregulating the expression of MMPs that cleave matrix proteins, chemokines that attract immune cells, and *Cd44* that mediates cell adhesion/migration via hyaluronate/matrix attachment. Similarly, based on the existing role of chemokines, their upregulation may further augment activity/gene expression of chemokines and their receptors, such as *Ccl7*, *Ccl9*, *Ccl13*, *Ccr1*, *Ccr5* and *Pf4* (*Cxcl4*) that are important for amplification of immune response and recruitment of immune cells to the site of inflammation.

Simultaneous with persistent inflammation in the cartilage with Grade 2 damage, the suppression of genes involving matrix synthesis in *Cluster V* was observed (score 39, Figure 7B). IPA network analysis suggested that the major foci of the molecular network suppressed were TGF-β complex, *Ig fbp*, *Ctg f* and *Eg f*. Suppression of these genes may have downregulated matrix proteins such as collagens (-type II alpha-1, -type X alpha1, -type XI alpha-1 and -2), and molecules involved in matrix synthesis such as *Adamts3* and *Hapln1* (stabilizes cartilage matrix). More importantly, a significant suppression of TGF-β complex in this network may have also downregulated many genes associated with bone formation such as *Bglap*, *Dlx5*, *Alpl*, and *Bmpr1*. The downregulation of these genes during chronic inflammation may result in the failure of matrix repair, thus accelerating the damage.

In the major molecular network in *Cluster III* (score 29, Figure 8A), related to pathologies observed in Grade 3–3.5 cartilage damage, many of the genes were associated with immune suppression and adaptation such as *Socs3*, *Osmr*, *Gas7* and *Il10rb* [28]. Interestingly, at this stage, except for IL-15, the upregulation of other inflammation-associated genes such as NF-κB complex, IL-1 complex, IFN alpha and IFN beta complex, MHC complex, and IL-12, was not evident. However, several genes that are associated with B cell, T cell and macrophage proliferation, differentiation, and migration, such as complement cascade (innate immunity and macrophage activation), IL-15 (stimulates T-lymphocyte proliferation), and interferon-induced transmembrane protein 3 (Ifitm3, mediates cellular immunity) were upregulated.

## Discussion

To the best of our knowledge, this study documents the first evidence of temporally controlled global gene regulation and identifies the major determining molecular networks that likely control the progression of cartilage damage in a well-established rat model of MIA. We examined changes in the gene expression profiles by transcriptome-wide microarray analysis in relation to the progression of MIA determined by macroscopic, microscopic, and µCT imaging to assess bone involvement [22], [29], [30], [31]. This model of experimental OA was considered useful due to its similarities to the pathogenesis of OA, reproducibility, reasonable duration of the test period, and ability to induce cartilage damage without confounding effects of surgical wounding on the joint tissues [21], [22], [29]. In this experimental model, the first 3 weeks of MIA progression showed major changes in the cartilage destruction and Grade 6 damage is achieved over a period of 8 weeks (56 days) [22]. After 3 weeks of MIA progression, the cartilage loss is slowly replaced by fibrocartilage and bone. Therefore, we have focused on the initial period of 3 weeks (21 days) where the cartilage damage advanced to Grade 3–3.5. Although the progression of MIA in this model was much faster, it exhibited a sequential progression of cartilage damage observed over a longer period of time in other models of OA. Furthermore, as described earlier, less than 2% cell death was observed due to the monoiodoacetate-induced injury on day 1 after monoiodoacetate injection [32]. Nevertheless, rodent models cannot depict arthritis exactly to humans, as the joint mechanics differ in small quadrupeds [33].

The foremost findings from the transcriptome-wide gene expression profiles are that the MIA afflicted cartilage showed stage specific reproducible changes in gene expression, as demonstrated by the hierarchical and partition clustering analyses. Strikingly, MIA progression involves up- or downregulation of approximately 7.44% of the transcripts by more than two-fold, at one or more time points (*p*<0.05). Furthermore, discrete sets of genes at each stage of cartilage damage appear to maximally regulate set of genes associated with inflammation and ECM degradation.

The overall gene expression profiles and the IPA derived from these profiles suggest that Grade 1 cartilage damage is likely associated with upregulation of genes required for: (i) acute inflammation/innate immunity such as superoxides, complement components, integrins, IL-1/IL-1r, chemokines and their receptors, and monocyte activating factors; (ii) chondrocyte-matrix interactions, i.e., versican, fibulin and microfibril; (iii) cleavage of matrix and cell associated proteins such as broad specificity proteases cathepsins and heparanase [34]; and (iv) cell proliferation such as cell cycle/proliferation and mitogenic growth factors (FGF7 and CSF receptor b). The presence of proliferating cells may support increased cell division in the cartilage with Grade 1 damage (Figure 1g). In parallel, suppression of genes essential for: (i) proteoglycan synthesis/assembly, i.e., *Cilp* and *Cilp2*, *Acan*, *Bgn*, *Eln*, *Sdc2* and cell-matrix adhesion such as Col XVI 1α, and ColXVII 1α; and (ii) inhibitors of peptidases (*Timp3*, *Serpina3*, *Pi15*), may further support increased proteolytic breakdown of cartilage matrix. More importantly, upregulation of asporin that mediates downregulation of TGF-β activity, and consequently suppression of *Sox9* may be responsible for the dramatic suppression of the above proteoglycan-associated genes. Interestingly, genes such as asporin, IL-1β, IL-1 receptor-like 1, cathepsin S, PGE receptor (EP4) and integrins are also upregulated in OA in humans and experimental animals, suggesting their possible role in the early stages of the disease progression [35], [36], [37], [38], [39], [40], [41], [42].

The most dramatic matrix breakdown and deficit in ECM synthesis might occur in cartilage with Grade 2 damage. Overall, the gene regulation in cartilage with Grade 2 damage was focused around: (i) Chronic inflammation (chemokines and their receptors) and the NF-κB signaling cascade, which not only regulates inflammation and apoptosis, but also matrix degradation [43]; and (ii) Matrix breakdown, via increased expression of metalloproteases (MMP-9, -12, and -19, ADAMTS4, ADAMTS 7, ADAMTS 12, *Hyal1*, *Hyal3*) primarily responsible for cleaving collagens and proteoglycans. In parallel suppression of genes associated with: (i) ECM such as matrilin-3 and -1, major collagens (type IIα1, -Xα1, -IXα1, -IXα2, -IXα3, -XIα1, -XIα2), *Chad*, *Prg4*; (ii) matrix synthesis/assembly (*Adamts3*, *Adamtsl3*, *Hs3stb1*, *Hs3st1*, *Chsy3*, *Has2*, *Arsg*, *Alpl*); (iii) growth factors and their receptors (*Omd*, *Fg fr2*, *Fg fr3*, *Ctg f*, *Bmp5*, *Ghr*, *Bmp3*, *Bmpr1a*, *Eg f *); and (iv) signaling molecules (*Frzb*, *Lect1*, *Sfrp5*, *Fzd9*, *Sox6*, *Wisp3*, *Dlx5*, *Mad3*, *Smad9*) may further suppress cartilage and bone formation in these advanced lesions. Our findings further confirm earlier studies where significant upregulation of MMPs, ADAMTS4, arachidonate-5-lipoxygenase, and IL-18 was observed in both human and experimental OA [10], [12], [13], [15], [39], [44], [45], [46], [47], [48], [49], [50].

Interestingly, cartilage with Grade 3–3.5 damage showed successive upregulation of genes from Grade 1 to Grade 3–3.5, and those genes were involved in: (i) inflammation and immune adaptation via relative downregulation of proinflammatory molecules IL-1, TNF, NF-kB signaling; (ii) suppression of inflammation (*Socs3*, *Il10rb*, *Lilbr4*, *C1s*, *Nfkbia*); (iii) LPS/pathogen/antigen recognition/clearance (*Lbp*, *Tlr4*, *Cd14*) [51], [52], [53]; (iv) ECM for bone formation (*Postn*, *Ogn*, *Tnn*) likely associated with repair/formation of osteophyte; and (v) anti-angiogenic collagens (Col XVIIIα1, -IVα1 -IVα2) and collagens associated with soft tissue and collagen type 1 (Col XII1α1, -IIIα1, -Vα1), suggesting that these genes may be important in the replacing damaged cartilage with bone and fibrous tissue in OA (Figure 1o). In fact, collective upregulation of many of these genes (*Tnn*, *Postn*, *Vegf*, *Vcam1*) has also been reported in later stages of experimental and human OA [10], [54], [55], [56], [57], [58]. However, it should be noted that these attempts to remodel seemed to occur under the influences of damage-associated molecular patterns (DAMPs) since sustained overexpression of DAMP-related ligand (*Vcan*), mediators (*Cd14* and *Ly96*), and signaling receptors (*Tlr4* and *Tlr7*) was observed [59].

Interestingly, the expression of many genes for ECM proteins (*Col2a1*, *Col10a1*, *Col9a1*, *Col9a2*, *Col9a3*, *Col27a1*, *Matn3*, *Chad*, *Prelp*, *Hapln1*, *Fbln5*, *Fbln7*, *Prg4*, *Acan*, *Sdc1*, *Cilp*, *Cilp2*, *Ntn1*, *Spon2*, *Eln*, *Bgn*, *Sdc4*) was relatively increased in the cartilage with Grade 3–3.5 damage as compared to cartilage with less damage (Grade 1 and 2). At the same time, gene expression of peptidases involved in matrix breakdown (*Pi15*, *Chst3*, *Chst3*, *Mmp12*, *Mmp19*, *Adamts4*, *Adamts12*, *Mmp9*, *Hyal1*, *Hyal3*, *Arsb*, *Gusb*) was relatively downregulated in this stage as compared to earlier stages. In this respect, earlier studies have shown that cartilage from later stages of human OA show increased synthesis of certain matrix proteins in comparison to cartilage from early OA [10], [11], [60]. The basis for this increased expression of matrix associated genes in late stage cartilage degradation (Grade 3–3.5) is as yet not clear. However, it is likely that lesser suppression of TGF-β1, *Ctgf*, and *Sox9* as well as antiinflammatory molecules, may support matrix induction. Additionally, upregulation of gene expression of bone formation related molecules (*Tgfb2*, *Pdgfc*, *Pdgfrb*, *Ogn*, *Egfr*, *Ctgf*, *Igfbp*,*Bmp5*, *Bmpr1a*, *Fgfr2*, *Fgfr3*, and molecules of Wnt signaling cascade) in Grade 3–3.5 cartilage damage may also play a role in the upregulation of matrix genes. Many of these molecules are also upregulated in cartilage from human OA [11], [13], [39], [41], [56], [61], [62], [63].

**Figure 9:**
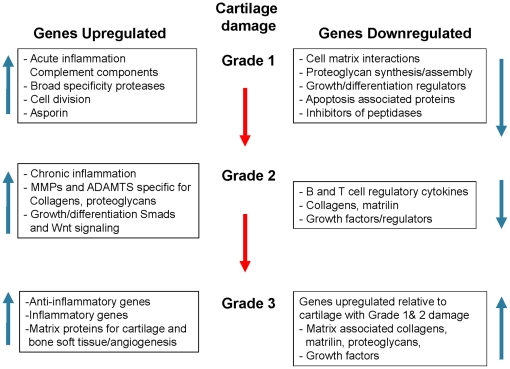
Schematic presentation of collective catabolic and anabolic gene regulation during the progression of MIA. Grade 1 damage in the cartilage was associated with induction of genes required for acute inflammation and innate immunity, broad specificity proteases, and cell cycle/division and suppression of genes for proteoglycan synthesis. Grade 2 damage in the cartilage was associated with induction of gene for NF-κB signaling cascade, inflammatory mediators/cytokines, metallopeptidases, and immune trafficking, and suppression of growth factors and collagens. Grade 3–3.5 damage in the cartilage exhibited upregulation of anti-inflammatory genes, and simultaneous reduction in the suppression of matrix-associated proteins and growth factors as compared to cartilage with Grade 1 or Grade 2 damage. Collective and sequential up and down regulation of these gens may be important in the cartilage damage during the progression of MIA.

In summary, the present study provides evidences that the progression of cartilage damage is driven by complex but precise regulation of gene clusters that are induced or suppressed during a specific stage of cartilage damage (Figure 9). Cartilage with close to Grade 1 damage exhibited upregulation of genes associated with acute inflammation and innate immunity, broad specificity proteases, and cell cycle/division and suppression of genes for proteoglycan synthesis. Gene expression in cartilage with Grade 2 damage was associated with dynamic upregulation of genes driven by NF-κB such as inflammatory mediators/cytokines, metallopeptidases, and immune trafficking. Chronic inflammation was paralleled by suppression of growth factors and collagens. Cartilage with Grade 3–3.5 damage exhibited an adaptive response evidenced by upregulation of anti-inflammatory genes. Simultaneously, there is a significant reduction in the suppression of matrix-associated proteins and growth factors as compared to cartilage with Grade 1 or Grade 2 damage. Collectively, the precise modulation of sequential up and down regulation of these genes may support the cartilage damage observed during the progression of MIA. Further elucidation of the key molecules that regulate the expression of catabolic as well as anabolic genes is critical in understanding the mechanisms of cartilage damage in experimental and human OA.

## Materials and Methods

### Monoiodoacetate-induced arthritis

The work was performed under the protocol number 2009A0138 approved by the Institutional Animal Care and Use Committee, The Ohio State University. Female Sprague-Dawley rats, 12–14 weeks old (Harlan Labs, IN) were randomly assigned to 4 groups (15 rats/group). The right knees of rats were given intra-articular injection of 50 µl saline in sham controls (*Cont*, n = 15), or monoiodoacetate (2 mg/50 µl saline) in experimental animals to induce MIA (n = 45). Following administration of monoiodoacetate, the cartilage exhibited Grade 1, Grade 2, or Grade 3–3.5 on days 5, 9, and 21, respectively. Therefore, progression of cartilage damage and changes in gene expression profiles were carried out on day 5 (*MIA5*; n = 15), day 9 (*MIA9*; n = 15), or day 21 (*MIA21*; n = 15) post-monoiodoacetate injection. Among them, 5 femurs from each group were snap-frozen in liquid nitrogen for microarray and real time-Polymerase Chain Reaction (rt-PCR) analyses (n = 5), and the remaining 10 femurs were immediately examined macroscopically using a stereomicroscope and then fixed in 10% buffered formalin for microscopic examination of the cartilage and bone, or µCT imaging to assess the overall subchondral bone loss.

### Macroscopic and microscopic examination

Gross morphologies of femurs were recorded photographically under a stereomicroscope. The microscopic examination was performed in paraffin embedded and Hematoxylin-Eosin (H&E) stained femurs. The cartilage damage was graded according to Pritzker *et al.* [9].

### MicroCT analysis

To assess the involvement of subchondral bone in MIA, the femurs were scanned at approximately 19.4 µm resolution on an Inveon microCT from Siemens Preclinical (Knoxville, TN). The scans were run as 220 degree half scans with a theta of 0.5 degrees, with 500 ms exposure, and 700 projections/360 degrees. The source for the acquisition was run at 80 kV and 500 mA with 0.5 mm of Al filtration for beam hardening. Analysis of images was conducted on an Inveon Research Workstation.

### RNA extraction and microarray analysis

The cartilage from the distal end of individual femur (10–15 mg/femur) was examined under a stereomicroscope (Zeiss, Germany). Superficial articular cartilage on the patellar and condylar surfaces of the distal ends of femur was chipped off in a frozen state, avoiding the areas immediately around lesions. The cartilage chips from each knee were collected separately, and pulverized into 1 µm fragments in a Mikrodismembrator S (Sartorious, France) at 2500 rpm for 30 seconds [32]. RNA was extracted with Trizol reagent (Invitrogen, CA), and each sample of RNA was analyzed in a 2100 Bioanalyzer (Agilent, CA) to ensure optimal quality of RNA [64].

A total of 300 ng of RNA was used for cDNA synthesis and labeling using Whole Transcript (WT) cDNA Synthesis and Amplification Kit, and WT Terminal Labeling Kit (Affymetrix, CA). The labeled samples were hybridized on Affymetrix GeneChip Rat Gene 1.0 ST Array and scanned at the Microarray Shared Resource Facility at the OSU Comprehensive Cancer Center.

The intensity scans from three biologically independent arrays per treatment were subjected to gene expression analysis using Partek Genomic Suite version 6.4 (Partek Inc., MO). The significance of differences among the conditions was calculated by the analysis of variance (ANOVA) and only significantly regulated transcripts (*p*<0.05) were considered for further analyses. Variations among the samples in each condition were examined by principal components analysis (PCA), and subjected to both hierarchical and partition clustering by Partek Genomic Suite. All data is MIAME compliant and the raw data has been deposited in a MIAME compliant database GEO (accession number GSE28958).

### Functional gene network analysis

The gene expression data derived from microarray analysis was used to generate functional and molecular networks through the use of IPA (Ingenuity Systems, CA). A fold-change cutoff of 2.0 was set to identify and assign the molecules to the Ingenuity's Knowledge Base. In these analyses, gene expression changes were considered in the context of physical, transcriptional or enzymatic interactions of the gene/gene products, and then grouped according to interacting gene networks at a particular point. The score assigned to any given gene network took into account the total number of molecules in the data set, the size of the network and the number of assigned network eligible genes/molecules in the data set at a given time point. The significance value and network score were based on the hypergeometric distribution and calculated with the right-tailed Fisher's exact test. The network score was the negative log of the *p* value.

### Validation of salient genes differentially expressed in cluster analysis

Expression of selected genes from clustering analysis was confirmed by rt-PCR as previously described [65]. Briefly, extracted RNA was subjected to first strand cDNA synthesis using the Superscript III Reverse Transcriptase Kit (Invitrogen, CA). Gene expression was assessed by amplifying the cDNA with custom-designed primers in the iCycler iQ Real-Time PCR System (Bio-Rad, CA). The primers used were: *Rps18*-sense 5′-GCGGCGGAAAATAGCCTTCG-3′, anti-sense 5′-GGCCAGTGGTCTTGGTGTGCTG-3′; *Ctss*-sense 5′-AAATCGAGCTGCCACGTGTT-3′, anti-sense 5′-TGGCCACTGCTTCTTTCAGAG-3′; *Il-1β*-sense 5′-TATGTCTTGCCCGTGGAGCTT-3′, anti-sense 5′-TAGCAGGTCGTCATCATCCCA-3′; *Mmp12*-sense 5′-CCAGGAAATGCAGCAGTTCTTT-3′; anti-sense 5′-GCTGTACATCAGGCACTCCACAT-3′; *Ccr1*-sense 5′-TCAGTGTGAGCAGAGCAAGCA-3′, anti-sense 5′-CCGCTCACCCACAAAGACATA-3′; *Alox5*-sense 5′-TTCTCCGCACACATCTGGTGT-3′, anti-sense 5′-GGCAATGGTGAACCTCACATG-3′; *Vcam1*-sense 5′-GCCGGTCATGGTCAAGTGTTT-3′, anti-sense 5′-CATGAGACGGTCACCCTTGAA-3′; *Cilp*-sense 5′-TGTGAAGTCCAAGGTCACCCA-3′, anti-sense 5′-GTAGAAGGAGTTGGTGGCATTCTG-3′; *Sox9*-sense 5′-ATCTGAAGAAGGAGAGCGAG-3′; anti-sense 5′-CAAGCTCTGGAGACTGCTGA-3′; *Col9a1*-sense 5′-TGATGGCTTTGCTGTGCTG-3′, anti-sense 5′-TGACTGGCAGTTCATGGCA-3′; *Col2a1*-sense 5′-ATGAGGGCCGAGGGCAACAG-3′, anti-sense 5′-GATGTCCATGGGTGCAATGTCAA-3′.

### Statistical Analysis

All time dependent analyses were performed on 15 animals per group. Microarray analyses were performed on cartilage extracted from three separate animals. The significance among the conditions in the microarray data was tested by Partek Genomic suite by ANOVA to render significantly regulated genes (*p*<0.05) during the progression of MIA at each time point. ANOVA with Tukey's HSD post hoc test by SPSS v 17 was used to determine the significance levels of rt-PCR data that include two additional independent samples per group to microarray-examined specimens (n = 5). *p*<0.05 was regarded as significant.

## Supporting Information

Figure S1 **Cell division associated molecular network in Cluster I by IPA.** The molecular network in Cluster I showing expression of significant number of genes associated with cell division in the cartilage with Grade 1 damage.
(TIF)

Table S1 **Changes in the expression of genes in** ***Cluster I*** **.** CD, genes involved in cell division, proliferation, apoptosis; ECM, extracellular matrix proteins; ECM2, Proteases, regulators of ECM synthesis and breakdown; GF, genes for growth factors and their receptors; GF2, growth factor signaling molecules, transcription factors; Inf, cytokines, chemokines and their receptors; Inf2, inflammatory mediators and their receptors, signaling molecules, transcription factors, and regulators; Meta, genes for metabolism; Others, genes with unknown functions; Transporter, genes involved in transportation of metabolites and ions.
(DOC)

Table S2 **Changes in the expression of genes in Cluster IV.** Please see Table S1 for group description.
(DOC)

Table S3 **Changes in the expression of genes in Cluster II.** Please see Table S1 for group description.
(DOC)

Table S4 **Changes in the expression of genes in Cluster V.** Please see Table S1 for group description.
(DOC)

Table S5 **Changes in the expression of genes in Cluster III.** Please see Table S1 for group description.
(DOC)

Movie S1 **360° µCT projection of the knee of** ***Cont*** **.**
(MPG)

Movie S2 **360° µCT projection of the knee of** ***MIA5*** **.**
(MPG)

Movie S3 **360° µCT projection of the knee of** ***MIA9*** **.**
(MPG)

Movie S4 **360° µCT projection of the knee of** ***MIA21*** **.**
(MPG)
