# Supplementary figures and images for: Sequential Alterations in Catabolic and Anabolic Gene Expression Parallel Pathological Changes during Progression of Monoiodoacetate-Induced Arthritis
Source: PLoS One. 2011 Sep 13;6(9):e24320. doi: 10.1371/journal.pone.0024320 (PMC3172226; doi:10.1371/journal.pone.0024320)

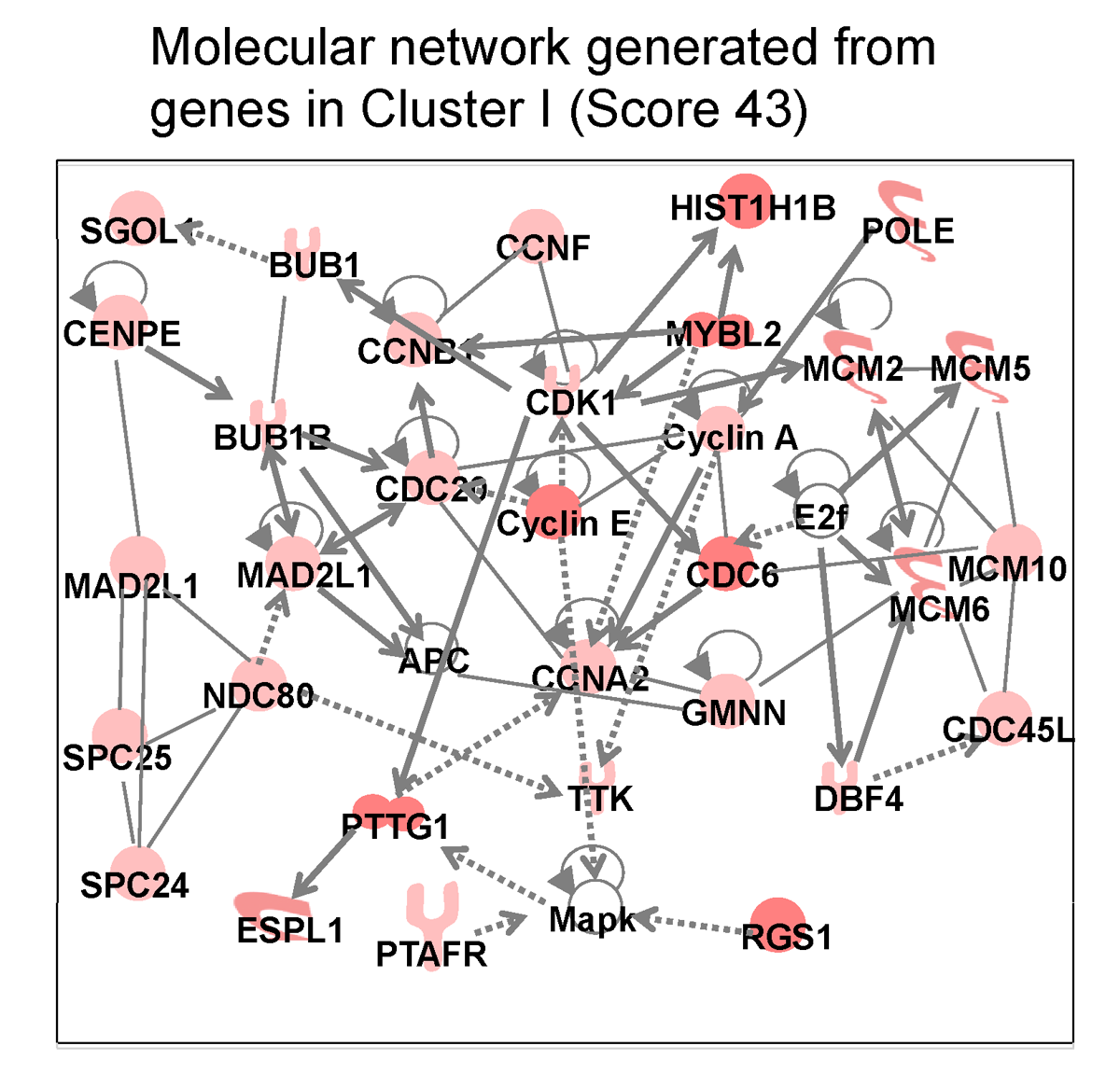

Supplement: Figure S1 — Cell division associated molecular network in Cluster I by IPA. The molecular network in Cluster I showing expression of significant number of genes associated with cell division in the cartilage with Grade 1 damage. (TIF) [file pone.0024320.s001.tif]
