# Supplementary material for: Sequential Alterations in Catabolic and Anabolic Gene Expression Parallel Pathological Changes during Progression of Monoiodoacetate-Induced Arthritis
Source: PLoS One. 2011 Sep 13;6(9):e24320. doi: 10.1371/journal.pone.0024320 (PMC3172226; doi:10.1371/journal.pone.0024320)
Supplement: Table S1 — Changes in the expression of genes in Cluster I . CD, genes involved in cell division, proliferation, apoptosis; ECM, extracellular matrix proteins; ECM2, Proteases, regulators of ECM synthesis and breakdown; GF, genes for growth factors and their receptors; GF2, growth factor signaling molecules, transcription factors; Inf, cytokines, chemokines and their receptors; Inf2, inflammatory mediators and their receptors, signaling molecules, transcription factors, and regulators; Meta, genes for metabolism; Others, genes with unknown functions; Transporter, genes involved in transportation of metabolites and ions. (DOC) [file pone.0024320.s002.doc]

**Table S1**. Changes in the expression of genes in *Cluster I*. **CD**, genes involved in cell division, proliferation, apoptosis; **ECM**, extracellular matrix proteins; **ECM2**, Proteases, regulators of ECM synthesis and breakdown; **GF**, genes for growth factors and their receptors; **GF2**, growth factor signaling molecules, transcription factors; **Inf**, cytokines, chemokines and their receptors; **Inf2**, inflammatory mediators and their receptors, signaling molecules, transcription factors, and regulators; **Meta**, genes for metabolism; **Others**, genes with unknown functions; **Transporter**, genes involved in transportation of metabolites and ions.

| Gene | Group | Description | MIA5 | MIA9 | MIA21 |
| --- | --- | --- | --- | --- | --- |
| Aspm | CD | asp (abnormal spindle) homolog, microcephaly associated (Drosophila) | 2.63 | 1.72 | -1.00 |
| Aurka | CD | aurora kinase A | 2.10 | 1.62 | 1.01 |
| Bub1 | CD | budding uninhibited by benzimidazoles 1 homolog (yeast) | 2.82 | 1.74 | 1.11 |
| Bub1b | CD | budding uninhibited by benzimidazoles 1 homolog beta (yeast) | 2.54 | 1.68 | -1.24 |
| Card6 | CD | caspase recruitment domain family, member 6 | 2.43 | 1.96 | 2.00 |
| Casp1 | CD | caspase 1, apoptosis-related cysteine peptidase (interleukin 1, beta, convertase) | 2.09 | 1.53 | 1.14 |
| Ccdc82 | CD | coiled-coil domain containing 82 | 2.06 | 1.06 | 1.13 |
| Ccdc99 | CD | coiled-coil domain containing 99 | 2.25 | 1.56 | 1.23 |
| Ccna2 | CD | cyclin A2 | 2.05 | 1.50 | 1.03 |
| Ccnb1 | CD | cyclin B1 | 2.95 | 2.11 | -1.14 |
| Ccnb2 | CD | cyclin B2 | 2.44 | 2.03 | -1.21 |
| Ccne1 | CD | cyclin E1 | 2.63 | 1.51 | 1.02 |
| Ccnf | CD | cyclin F | 2.54 | 1.71 | -1.15 |
| Cd53 | CD | CD53 molecule | 2.80 | 2.81 | 2.13 |
| Cdc2 | CD | cell division cycle 2, G1 to S and G2 to M | 2.39 | 1.76 | 1.06 |
| Cdc20 | CD | cell division cycle 20 homolog (S. cerevisiae) | 2.95 | 1.92 | 1.10 |
| Cdc45l | CD | CDC45 cell division cycle 45-like (S. cerevisiae) | 2.25 | 1.14 | -1.32 |
| Cdc6 | CD | cell division cycle 6 homolog (S. cerevisiae) | 3.14 | 1.36 | -1.31 |
| Cdca2 | CD | cell division cycle associated 2 | 2.30 | 1.70 | 1.02 |
| Cdca7 | CD | cell division cycle associated 7 | 1.54 | -1.26 | -2.05 |
| Cdk6 | CD | cyclin-dependent kinase 6 | 2.42 | 2.48 | 2.31 |
| Cdkn3 | CD | cyclin-dependent kinase inhibitor 3 | 2.33 | 1.54 | 1.06 |
| Cenpe | CD | centromere protein E, 312kDa | 2.74 | 1.92 | 1.21 |
| Cenpf | CD | centromere protein F, 350/400ka (mitosin) | 3.49 | 1.92 | 1.30 |
| Cenpi | CD | centromere protein I | 2.56 | 1.93 | 1.17 |
| Clspn | CD | claspin homolog (Xenopus laevis) | 2.17 | 1.22 | -1.04 |
| Dbf4 | CD | DBF4 homolog (S. cerevisiae) | 2.23 | 1.64 | 1.24 |
| Dlgap5 | CD | discs, large (Drosophila) homolog-associated protein 5 | 2.35 | 1.09 | -1.05 |
| Dock2 | CD | dedicator of cytokinesis 2 | 2.31 | 1.49 | -1.10 |
| Dtl | CD | denticleless homolog (Drosophila) | 2.39 | 1.36 | -1.10 |
| Dusp6 | CD | dual specificity phosphatase 6 | 3.28 | 3.41 | 2.89 |
| Ebf1 | CD | early B-cell factor 1 | 2.08 | 1.20 | 1.59 |
| Ect2 | CD | epithelial cell transforming sequence 2 oncogene | 3.49 | 2.23 | 1.29 |
| Emb | CD | embigin homolog (mouse) | 4.09 | 4.00 | 2.00 |
| Fancd2 | CD | Fanconi anemia, complementation group D2 | 2.77 | 1.65 | -1.03 |
| Gmnn | CD | geminin, DNA replication inhibitor | 2.50 | 1.83 | -1.12 |
| Kif11 | CD | kinesin family member 11 | 2.65 | 1.81 | 1.04 |
| Kif15 | CD | kinesin family member 15 | 2.07 | 1.21 | -1.14 |
| Kif20b | CD | kinesin family member 20B | 2.14 | 1.35 | -1.08 |
| Kif22 | CD | kinesin family member 22 | 2.81 | 1.92 | 1.33 |
| Kif23 | CD | kinesin family member 23 | 3.56 | 2.10 | 1.31 |
| Kif2c | CD | kinesin family member 2C | 2.46 | 1.76 | 1.00 |
| Kif4 | CD | kinesin family member 4A | 3.72 | 2.08 | 1.16 |
| Kntc1 | CD | kinetochore associated 1 | 3.12 | 1.72 | 1.12 |
| Map1b | CD | microtubule-associated protein 1B | 2.96 | 1.99 | 2.31 |
| Mlf1ip | CD | MLF1 interacting protein | 2.39 | 1.65 | 1.11 |
| Mybl2 | CD | v-myb myeloblastosis viral oncogene homolog (avian)-like 2 | 2.87 | 2.03 | -1.19 |
| Ncapd3 | CD | non-SMC condensin II complex, subunit D3 | 2.17 | 1.40 | 1.14 |
| Ndc80 | CD | NDC80 homolog, kinetochore complex component (S. cerevisiae) | 2.52 | 1.50 | -1.02 |
| Nlrc4 | CD | NLR family, CARD domain containing 4 | 2.58 | 1.35 | 1.04 |
| Plk4 | CD | polo-like kinase 4 (Drosophila) | 3.00 | 1.81 | 1.06 |
| Prc1 | CD | protein regulator of cytokinesis 1 | 3.34 | 2.43 | 1.67 |
| Ptprc | CD | protein tyrosine phosphatase, receptor type, C | 3.40 | 2.68 | 2.32 |
| Ptprn | CD | protein tyrosine phosphatase, receptor type, N | 4.42 | 3.37 | 1.76 |
| Sgol2 | CD | shugoshin-like 2 (S. pombe) | 2.76 | 1.54 | -1.04 |
| Spc24 | CD | SPC24, NDC80 kinetochore complex component, homolog (S. cerevisiae) | 3.48 | 2.34 | 1.27 |
| Spc25 | CD | SPC25, NDC80 kinetochore complex component, homolog (S. cerevisiae) | 2.04 | 1.60 | -1.05 |
| Tpx2 | CD | TPX2, microtubule-associated, homolog (Xenopus laevis) | 3.04 | 1.81 | 1.02 |
| Vav1 | CD | vav 1 guanine nucleotide exchange factor | 3.08 | 2.62 | 1.69 |
| Zwilch | CD | Zwilch, kinetochore associated, homolog (Drosophila) | 2.04 | 1.24 | -1.01 |
| Emilin2 | ECM | elastin microfibril interfacer 2 | 2.70 | 2.36 | 2.07 |
| Fbln2 | ECM | fibulin 2 | 6.56 | 4.12 | 2.87 |
| Fbn1 | ECM | fibrillin 1 | 2.65 | 2.02 | 1.84 |
| Flnc | ECM | filamin C, gamma | 3.34 | 3.03 | 2.39 |
| Mfap5 | ECM | microfibrillar associated protein 5 | 6.61 | 4.02 | 3.48 |
| Spon1 | ECM | spondin 1, extracellular matrix protein | 2.93 | 2.26 | 2.56 |
| Srgn | ECM | serglycin | 1.82 | -1.21 | -2.63 |
| Vcan | ECM | versican | 9.09 | 7.96 | 6.36 |
| Adamts18 | ECM2 | ADAM metallopeptidase with thrombospondin type 1 motif, 18 | 1.42 | -2.64 | -2.44 |
| Arsb | ECM2 | arylsulfatase B | 3.75 | 3.71 | 2.62 |
| Ctsc | ECM2 | cathepsin C | 3.72 | 3.66 | 3.29 |
| Ctsg | ECM2 | cathepsin G | 1.31 | -1.61 | -2.58 |
| Ctss | ECM2 | cathepsin S | 3.92 | 3.21 | 3.69 |
| Dpp4 | ECM2 | dipeptidyl-peptidase 4 | 1.17 | -1.83 | -2.68 |
| Ela2 | ECM2 | elastase, neutrophil expressed | 1.31 | -2.29 | -3.96 |
| Hpse | ECM2 | heparanase | 6.42 | 3.60 | 3.31 |
| Phex | ECM2 | phosphate regulating endopeptidase homolog, X-linked | 1.02 | -4.75 | -3.41 |
| Prss29 | ECM2 | protease, serine, 29 | 2.21 | 1.16 | 1.04 |
| Prtn3 | ECM2 | proteinase 3 | 1.20 | -1.60 | -2.12 |
| Spint1 | ECM2 | serine peptidase inhibitor, Kunitz type 1 | 2.21 | 1.50 | 1.05 |
| Aspn | GF | asporin | 9.33 | 5.77 | 8.38 |
| Csf2rb | GF | colony stimulating factor 2 receptor, beta, low-affinity (granulocyte-macrophage) | 2.76 | 2.14 | 1.55 |
| Csf3r | GF | colony stimulating factor 3 receptor (granulocyte) | 2.34 | 1.51 | -1.29 |
| Fgf7 | GF | fibroblast growth factor 7 (keratinocyte growth factor) | 2.90 | -1.01 | 1.14 |
| Igfbp3 | GF | insulin-like growth factor binding protein 3 | 2.33 | 2.44 | 2.16 |
| Dkk1 | GF2 | dickkopf homolog 1 (Xenopus laevis) | 1.25 | -4.00 | -2.74 |
| Fzd1 | GF2 | frizzled homolog 1 (Drosophila) | 2.36 | 1.77 | 2.12 |
| Pcdh19 | GF2 | protocadherin 19 | 2.72 | 1.79 | 1.89 |
| Ptpn6 | GF2 | protein tyrosine phosphatase, non-receptor type 6 | 2.35 | 2.25 | 1.38 |
| Sfrp1 | GF2 | secreted frizzled-related protein 1 | 3.59 | 3.07 | 1.95 |
| Sfrp2 | GF2 | secreted frizzled-related protein 2 | 4.05 | -1.07 | 3.06 |
| Ccr2 | Inf | chemokine (C-C motif) receptor 2 | 2.30 | 2.07 | 1.02 |
| Clec4a1 | Inf | C-type lectin domain family 4, member a1 | 7.51 | 4.58 | 3.72 |
| Clec4a3 | Inf | C-type lectin domain family 4, member a3 | 4.97 | 4.26 | 4.32 |
| Clec7a | Inf | C-type lectin domain family 7, member A | 1.93 | 2.03 | 1.56 |
| Clecsf6 | Inf | C-type (calcium dependent, carbohydrate recognition domain) lectin, superfamily member 6 | 3.40 | 3.56 | 2.72 |
| Cmtm6 | Inf | CKLF-like MARVEL transmembrane domain containing 6 | 2.00 | 1.98 | 1.88 |
| Cxcl12 | Inf | chemokine (C-X-C motif) ligand 12 (stromal cell-derived factor 1) | 1.14 | -2.11 | -1.74 |
| Ifngr1 | Inf | interferon gamma receptor 1 | 2.24 | 1.89 | 1.80 |
| Il10ra | Inf | interleukin 10 receptor, alpha | 2.52 | 1.82 | 1.72 |
| Il1b | Inf | interleukin 1, beta | 2.22 | 2.00 | 1.54 |
| Il1rl1 | Inf | interleukin 1 receptor-like 1 | 8.61 | 3.50 | 2.12 |
| Il21r | Inf | interleukin 21 receptor | 2.26 | 1.94 | 1.41 |
| Il33 | Inf | interleukin 33 | 2.01 | -1.02 | 1.15 |
| Il8rb | Inf | interleukin 8 receptor, beta | 2.68 | 1.82 | 1.49 |
| Ly49si2 | Inf | immunoreceptor Ly49si2 | 3.80 | 1.46 | 1.55 |
| Msr1 | Inf | macrophage scavenger receptor 1 | 4.76 | 4.09 | 4.31 |
| Ptafr | Inf | platelet-activating factor receptor | 2.05 | 1.68 | 1.34 |
| Tlr7 | Inf | toll-like receptor 7 | 5.07 | 2.75 | 3.23 |
| Alox5ap | Inf2 | arachidonate 5-lipoxygenase-activating protein | 2.49 | 2.06 | 2.19 |
| Angpt1 | Inf2 | angiopoietin 1 | 1.01 | -1.96 | -2.06 |
| Arhgap30 | Inf2 | Rho GTPase activating protein 30 | 2.49 | 2.22 | 1.36 |
| Arhgap4 | Inf2 | Rho GTPase activating protein 4 | 2.04 | 1.62 | 1.04 |
| Btk | Inf2 | Bruton agammaglobulinemia tyrosine kinase | 2.14 | 2.16 | 1.45 |
| C3ar1 | Inf2 | complement component 3a receptor 1 | 4.92 | 3.18 | 3.73 |
| C6 | Inf2 | complement component 6 | 1.34 | -2.38 | 1.38 |
| Cd180 | Inf2 | CD180 molecule | 2.52 | 2.07 | 1.85 |
| Cd244 | Inf2 | CD244 molecule, natural killer cell receptor 2B4 | 2.01 | 1.51 | 1.23 |
| Cd248 | Inf2 | CD248 molecule, endosialin | 3.06 | 2.85 | 2.57 |
| Cd300a | Inf2 | CD300a | 2.22 | 1.43 | 1.33 |
| Cd300le | Inf2 | CD300 molecule-like family member E | 5.25 | 3.26 | 3.65 |
| Cd74 | Inf2 | CD74 molecule, major histocompatibility complex, class II invariant chain | 3.03 | 1.58 | 1.34 |
| Cd86 | Inf2 | CD86 molecule | 2.09 | 1.67 | 1.63 |
| Ceacam1 | Inf2 | carcinoembryonic antigen-related cell adhesion molecule 1 (biliary glycoprotein) | 2.06 | -1.20 | -1.57 |
| Clec5a | Inf2 | C-type lectin domain family 5, member A | 2.60 | 2.64 | 2.26 |
| Dcir2 | Inf2 | dendritic cell inhibitory receptor 2 | 3.44 | 3.38 | 1.67 |
| Defa | Inf2 | defensin, alpha 5, Paneth cell-specific | 1.51 | -2.02 | -6.61 |
| Defa11 | Inf2 | defensin alpha 11 | 1.99 | -1.15 | -2.08 |
| Erbb3 | Inf2 | v-erb-b2 erythroblastic leukemia viral oncogene homolog 3 (avian) | 2.61 | 2.50 | 1.94 |
| Fanca | Inf2 | Fanconi anemia, complementation group A | 2.45 | 1.46 | -1.03 |
| Fcgr1a | Inf2 | Fc fragment of IgG, high affinity Ia, receptor (CD64) | 4.26 | 3.04 | 3.01 |
| Fcgr2a | Inf2 | Fc fragment of IgG, low affinity IIa, receptor (CD32) | 3.09 | 3.34 | 2.92 |
| Fcgr2b | Inf2 | Fc fragment of IgG, low affinity IIb, receptor (CD32) | 2.63 | 1.86 | 1.32 |
| Fcrls | Inf2 | Fc receptor-like 2 | 8.04 | 1.82 | 3.55 |
| Figf | Inf2 | c-fos induced growth factor (vascular endothelial growth factor D) | 2.44 | 1.30 | 1.84 |
| Fkbp5 | Inf2 | FK506 binding protein 5 | 2.42 | 1.35 | 1.96 |
| Flt3 | Inf2 | fms-related tyrosine kinase 3 | 2.19 | 1.14 | 1.13 |
| Gpr65 | Inf2 | G protein-coupled receptor 65 | 3.64 | 2.51 | 2.41 |
| H2-Ea | Inf2 | major histocompatibility complex, class II, DR alpha | 4.31 | 1.77 | 1.04 |
| Hla-dma | Inf2 | major histocompatibility complex, class II, DM alpha | 2.92 | 1.58 | 1.37 |
| Hla-dmb | Inf2 | major histocompatibility complex, class II, DM beta | 4.65 | 2.93 | 2.25 |
| Ifitm1 | Inf2 | interferon induced transmembrane protein 1 (9-27) | 2.37 | 1.72 | -1.01 |
| Igsf6 | Inf2 | immunoglobulin superfamily, member 6 | 2.67 | 1.88 | 1.77 |
| Irf5 | Inf2 | interferon regulatory factor 5 | 2.77 | 2.89 | 2.40 |
| Irf8 | Inf2 | interferon regulatory factor 8 | 2.50 | 1.05 | -1.16 |
| Itga4 | Inf2 | integrin, alpha 4 (antigen CD49D, alpha 4 subunit of VLA-4 receptor) | 2.03 | 1.61 | 1.37 |
| Itgal | Inf2 | integrin, alpha L (antigen CD11A (p180), lymphocyte function-associated antigen 1; alpha polypeptide) | 3.84 | 3.41 | 2.29 |
| Itgb2 | Inf2 | integrin, beta 2 (complement component 3 receptor 3 and 4 subunit) | 4.10 | 4.19 | 3.44 |
| Lef1 | Inf2 | lymphoid enhancer-binding factor 1 | 1.18 | -1.64 | -2.31 |
| Lilrb3 | Inf2 | leukocyte immunoglobulin-like receptor, subfamily B (with TM and ITIM domains), member 3 | 2.12 | 1.38 | 1.06 |
| Lilrb3l | Inf2 | leukocyte immunoglobulin-like receptor, subfamily B (with TM and ITIM domains), member 3-like | 2.61 | 1.32 | 1.37 |
| Lpar6 | Inf2 | lysophosphatidic acid receptor 6 | 3.87 | 2.79 | 2.53 |
| Lrp4 | Inf2 | low density lipoprotein receptor-related protein 4 | 1.21 | -2.10 | -1.32 |
| Ly49si1 | Inf2 | immunoreceptor Ly49si1 | 2.49 | 1.29 | 1.19 |
| Mcpt2 | Inf2 | mast cell protease 1 | 2.00 | 1.52 | -1.06 |
| Mgst2 | Inf2 | microsomal glutathione S-transferase 2 | 2.97 | 3.00 | 1.60 |
| Mmd | Inf2 | monocyte to macrophage differentiation-associated | 3.34 | 2.79 | 2.11 |
| Mpeg1 | Inf2 | macrophage expressed 1 | 3.36 | 2.38 | 2.14 |
| Ncf1 | Inf2 | neutrophil cytosolic factor 1 | 2.49 | 2.05 | 1.34 |
| Ncf2 | Inf2 | neutrophil cytosolic factor 2 | 2.74 | 2.77 | 1.77 |
| Ncf4 | Inf2 | neutrophil cytosolic factor 4, 40kDa | 2.34 | 2.05 | 1.67 |
| Neto2 | Inf2 | neuropilin (NRP) and tolloid (TLL)-like 2 | 2.26 | 1.88 | 1.53 |
| Nfam1 | Inf2 | NFAT activating protein with ITAM motif 1 | 2.26 | 2.10 | 1.78 |
| Nkg7 | Inf2 | natural killer cell group 7 sequence | 2.03 | -1.31 | -3.12 |
| Np4 | Inf2 | defensin, alpha 5, Paneth cell-specific | 1.69 | -1.25 | -3.44 |
| Pak3 | Inf2 | p21 protein (Cdc42/Rac)-activated kinase 3 | 2.04 | 1.56 | 1.57 |
| Pde1a | Inf2 | phosphodiesterase 1A, calmodulin-dependent | 3.11 | 1.63 | 2.53 |
| Pde5a | Inf2 | phosphodiesterase 5A, cGMP-specific | 2.37 | 1.73 | 1.80 |
| Plau | Inf2 | plasminogen activator, urokinase | 2.84 | 2.80 | 1.62 |
| Plcb2 | Inf2 | phospholipase C, beta 2 | 2.35 | 1.99 | 1.34 |
| Pld4 | Inf2 | phospholipase D family, member 4 | 2.82 | 2.20 | 1.56 |
| Plxnc1 | Inf2 | plexin C1 | 2.24 | 1.64 | -1.06 |
| Ptger2 | Inf2 | prostaglandin E receptor 2 (subtype EP2), 53kDa | 2.09 | 2.18 | 1.57 |
| Ptger4 | Inf2 | prostaglandin E receptor 4 (subtype EP4) | 4.56 | 2.81 | 2.44 |
| Ptgfr | Inf2 | prostaglandin F receptor (FP) | 2.91 | 2.04 | 2.37 |
| Pycard | Inf2 | PYD and CARD domain containing | 2.04 | 1.51 | 1.28 |
| Rnf128 | Inf2 | E3 ubiquitin-protein ligase RNF128 | 2.01 | 1.98 | 1.68 |
| RT1-Ba | Inf2 | major histocompatibility complex, class II, DQ alpha 1 | 3.14 | 1.18 | 1.50 |
| RT1-CE10 | Inf2 | major histocompatibility complex, class I, C | 2.44 | 1.92 | 1.97 |
| Samhd1 | Inf2 | SAM domain and HD domain 1 | 2.13 | 1.78 | 1.22 |
| Sh2d1b | Inf2 | SH2 domain containing 1B | 3.39 | 2.50 | 2.00 |
| Slamf9 | Inf2 | SLAM family member 9 | 5.39 | 4.26 | 2.73 |
| Slit2 | Inf2 | slit homolog 2 (Drosophila) | 2.54 | 1.72 | 1.70 |
| Trem2 | Inf2 | triggering receptor expressed on myeloid cells 2 | 2.56 | 1.78 | 2.01 |
| Ybx1 | Inf2 | Y box binding protein 1 | 2.02 | 1.92 | 1.31 |
| Acsl4 | Meta | acyl-CoA synthetase long-chain family member 4 | 2.32 | 2.17 | 1.78 |
| Actb | Meta | actin, beta | 2.01 | 1.59 | 1.78 |
| Actg2 | Meta | actin, gamma 2, smooth muscle, enteric | 2.03 | 1.74 | 1.84 |
| Akr1c14 | Meta | aldo-keto reductase family 1, member C14 | 1.57 | -2.02 | 1.23 |
| Anln | Meta | anillin, actin binding protein | 4.23 | 2.87 | 2.03 |
| Aoah | Meta | acyloxyacyl hydrolase (neutrophil) | 2.39 | 1.55 | 1.22 |
| Apobec1 | Meta | apolipoprotein B mRNA editing enzyme, catalytic polypeptide 1 | 3.39 | 2.32 | 2.85 |
| Arhgap25 | Meta | Rho GTPase activating protein 25 | 2.40 | 2.27 | 1.56 |
| Arl11 | Meta | ADP-ribosylation factor-like 11 | 3.62 | 3.04 | 2.59 |
| Asap1 | Meta | ArfGAP with SH3 domain, ankyrin repeat and PH domain 1 | 2.22 | 2.02 | 2.10 |
| Atp13a3 | Meta | ATPase type 13A3 | 2.06 | 1.71 | 1.32 |
| B3gnt5 | Meta | UDP-GlcNAc:betaGal beta-1,3-N-acetylglucosaminyltransferase 5 | 2.10 | 2.07 | 1.28 |
| B4galt6 | Meta | UDP-Gal:betaGlcNAc beta 1,4- galactosyltransferase, polypeptide 6 | 2.34 | 1.71 | 1.49 |
| Brip1 | Meta | BRCA1 interacting protein C-terminal helicase 1 | 2.40 | 1.40 | 1.09 |
| Bzw2 | Meta | basic leucine zipper and W2 domains 2 | 2.04 | 1.70 | 1.33 |
| Calcr | Meta | calcitonin receptor | 2.52 | 1.95 | 2.12 |
| Cdc42ep2 | Meta | CDC42 effector protein (Rho GTPase binding) 2 | 2.18 | 2.18 | 1.74 |
| Cenpk | Meta | centromere protein K | 2.15 | 1.25 | 1.04 |
| Cenpn | Meta | centromere protein N | 2.25 | 1.43 | 1.27 |
| Cenpt | Meta | centromere protein T | 2.00 | 1.31 | -1.08 |
| Cep76 | Meta | centrosomal protein 76kDa | 2.23 | 1.94 | 1.56 |
| Chd7 | Meta | chromodomain helicase DNA binding protein 7 | 2.11 | 2.17 | 2.08 |
| Ckap2 | Meta | cytoskeleton associated protein 2 | 2.32 | 1.66 | 1.44 |
| Ckap2l | Meta | cytoskeleton associated protein 2-like | 2.48 | 1.99 | 1.36 |
| Cntf | Meta | ciliary neurotrophic factor | 2.33 | 1.99 | 1.53 |
| Cort | Meta | cortistatin | 2.03 | 1.27 | -1.05 |
| Cotl1 | Meta | coactosin-like 1 (Dictyostelium) | 3.02 | 3.14 | 2.04 |
| Cpt1a | Meta | carnitine palmitoyltransferase 1A (liver) | 2.35 | 2.04 | 1.88 |
| Cyba | Meta | cytochrome b-245, alpha polypeptide | 2.16 | 1.71 | 1.57 |
| Cybb | Meta | cytochrome b-245, beta polypeptide | 3.16 | 2.65 | 3.00 |
| Cyth4 | Meta | cytohesin 4 | 3.98 | 3.38 | 3.03 |
| Cytip | Meta | cytohesin 1 interacting protein | 2.31 | 1.97 | 1.85 |
| Diaph3 | Meta | diaphanous homolog 3 (Drosophila) | 4.60 | 3.36 | 2.26 |
| Edem2 | Meta | ER degradation enhancer, mannosidase alpha-like 2 | 2.16 | 1.77 | 1.58 |
| Emr1 | Meta | egf-like module containing, mucin-like, hormone receptor-like 1 | 3.06 | 1.75 | 1.87 |
| Enpp5 | Meta | ectonucleotide pyrophosphatase/phosphodiesterase 5 (putative function) | 2.64 | 2.16 | 2.31 |
| Epb4.9 | Meta | erythrocyte membrane protein band 4.9 (dematin) | 2.29 | 1.08 | -1.50 |
| Espl1 | Meta | extra spindle pole bodies homolog 1 (S. cerevisiae) | 2.25 | 1.51 | -1.17 |
| Exo1 | Meta | exonuclease 1 | 2.50 | 1.46 | -1.09 |
| Fblim1 | Meta | filamin binding LIM protein 1 | 2.15 | 1.98 | 1.52 |
| Fes | Meta | feline sarcoma oncogene | 2.30 | 1.70 | 1.41 |
| Fgr | Meta | Gardner-Rasheed feline sarcoma viral (v-fgr) oncogene homolog | 2.37 | 2.27 | 1.30 |
| Fhdc1 | Meta | FH2 domain containing 1 | 2.04 | 1.23 | -1.07 |
| Fhl2 | Meta | four and a half LIM domains 2 | 3.10 | 2.82 | 2.40 |
| Fmo1 | Meta | flavin containing monooxygenase 1 | 2.30 | -1.28 | -1.14 |
| Fnbp1 | Meta | formin binding protein 1 | 2.53 | 2.46 | 2.47 |
| Galc | Meta | galactosylceramidase | 3.04 | 2.47 | 2.35 |
| Gclc | Meta | glutamate-cysteine ligase, catalytic subunit | 2.80 | 2.25 | 2.47 |
| Glipr2 | Meta | GLI pathogenesis-related 2 | 4.05 | 3.80 | 3.23 |
| Gna15 | Meta | guanine nucleotide binding protein (G protein), alpha 15 (Gq class) | 2.62 | 1.64 | 1.73 |
| Hck | Meta | hemopoietic cell kinase | 2.06 | 1.63 | 1.15 |
| Hebp1 | Meta | heme binding protein 1 | 2.11 | 1.87 | 1.66 |
| Hells | Meta | helicase, lymphoid-specific | 2.60 | 1.25 | -1.09 |
| Hgf | Meta | hepatocyte growth factor (hepapoietin A; scatter factor) | 2.11 | 1.37 | 1.60 |
| Hist1h1b | Meta | histone cluster 1, H1b | 2.08 | 1.05 | -1.35 |
| Hmbs | Meta | hydroxymethylbilane synthase | 2.06 | 1.20 | 1.00 |
| Hmmr | Meta | hyaluronan-mediated motility receptor (RHAMM) | 2.51 | 1.63 | 1.08 |
| Kif18a | Meta | kinesin family member 18A | 2.53 | 1.37 | -1.02 |
| Kitlg | Meta | KIT ligand | 2.30 | 1.63 | 1.83 |
| Kmo | Meta | kynurenine 3-monooxygenase (kynurenine 3-hydroxylase) | 2.41 | 1.37 | -1.09 |
| Lcp1 | Meta | lymphocyte cytosolic protein 1 (L-plastin) | 3.32 | 3.34 | 2.47 |
| Lims1 | Meta | LIM and senescent cell antigen-like domains 1 | 2.29 | 2.30 | 2.23 |
| Lrrc25 | Meta | leucine rich repeat containing 25 | 2.53 | 2.33 | 1.67 |
| Lyz2 | Meta | lysozyme 2 | 2.76 | 2.63 | 2.36 |
| Mad2l1 | Meta | MAD2 mitotic arrest deficient-like 1 (yeast) | 2.07 | 1.36 | 1.04 |
| Man2a1 | Meta | mannosidase, alpha, class 2A, member 1 | 2.64 | 2.38 | 2.28 |
| Mastl | Meta | microtubule associated serine/threonine kinase-like | 2.68 | 1.92 | 1.40 |
| Mcm10 | Meta | minichromosome maintenance complex component 10 | 2.13 | -1.03 | -1.41 |
| Mcm2 | Meta | minichromosome maintenance complex component 2 | 2.37 | 1.50 | 1.06 |
| Mcm3 | Meta | minichromosome maintenance complex component 3 | 2.31 | 1.34 | -1.13 |
| Mcm5 | Meta | minichromosome maintenance complex component 5 | 2.59 | 1.58 | -1.17 |
| Mcm6 | Meta | minichromosome maintenance complex component 6 | 2.88 | 1.56 | -1.09 |
| Me2 | Meta | malic enzyme 2, NAD(+)-dependent, mitochondrial | 2.39 | 2.03 | 1.92 |
| Mrc1 | Meta | mannose receptor, C type 1 | 4.41 | 2.46 | 3.94 |
| Myo1f | Meta | myosin IF | 2.56 | 1.72 | 1.13 |
| Nav3 | Meta | neuron navigator 3 | 4.38 | 3.59 | 4.08 |
| Ncapg2 | Meta | non-SMC condensin II complex, subunit G2 | 2.36 | 1.54 | 1.30 |
| Ncoa4 | Meta | nuclear receptor coactivator 4 | 2.03 | 2.06 | 1.67 |
| Nek2 | Meta | exonuclease 1 | 2.25 | 1.60 | -1.13 |
| Nes | Meta | nestin | 2.11 | 1.91 | 2.00 |
| Nhedc2 | Meta | Na+/H+ exchanger domain containing 2 | 2.53 | 2.67 | 2.40 |
| Nlrp3 | Meta | NLR family, pyrin domain containing 3 | 2.29 | 1.20 | 1.25 |
| Npl | Meta | N-acetylneuraminate pyruvate lyase (dihydrodipicolinate synthase) | 2.73 | 1.98 | 1.66 |
| Nt5dc2 | Meta | 5'-nucleotidase domain containing 2 | 2.20 | 1.95 | 1.47 |
| Nuf2 | Meta | NUF2, NDC80 kinetochore complex component, homolog (S. cerevisiae) | 2.11 | 1.38 | -1.11 |
| Nusap1 | Meta | nucleolar and spindle associated protein 1 | 2.35 | 1.43 | 1.06 |
| Oasl2 | Meta | 2'-5' oligoadenylate synthetase-like 2 | 2.39 | 1.71 | 1.42 |
| Olr1584 | Meta | olfactory receptor 1584 | 2.04 | 1.12 | -1.11 |
| P2ry13 | Meta | purinergic receptor P2Y, G-protein coupled, 13 | 3.28 | 1.87 | 1.82 |
| P2ry6 | Meta | pyrimidinergic receptor P2Y, G-protein coupled, 6 | 2.01 | 1.48 | 1.82 |
| Panx3 | Meta | pannexin 3 | 1.07 | -2.95 | -1.34 |
| Parp12 | Meta | poly (ADP-ribose) polymerase family, member 12 | 2.08 | 2.07 | 1.77 |
| Parp14 | Meta | poly (ADP-ribose) polymerase family, member 14 | 2.34 | 1.91 | 1.91 |
| Parvg | Meta | parvin, gamma | 2.08 | 1.61 | 1.13 |
| Pbk | Meta | PDZ binding kinase | 2.94 | 1.58 | 1.07 |
| Pigy | Meta | phosphatidylinositol glycan anchor biosynthesis, class Y | 2.34 | 2.03 | 1.77 |
| Pik3cd | Meta | phosphoinositide-3-kinase, catalytic, delta polypeptide | 2.07 | 1.44 | -1.11 |
| Pik3cg | Meta | phosphoinositide-3-kinase, catalytic, gamma polypeptide | 2.93 | 1.94 | 1.51 |
| Pkp4 | Meta | plakophilin 4 | 2.22 | 2.10 | 1.87 |
| Pole | Meta | polymerase (DNA directed), epsilon | 2.33 | 1.32 | -1.23 |
| Psat1 | Meta | phosphoserine aminotransferase 1 | 3.37 | 1.96 | 1.62 |
| Pttg1 | Meta | pituitary tumor-transforming 1 | 2.07 | 1.85 | -1.38 |
| Qprt | Meta | quinolinate phosphoribosyltransferase | 2.40 | 1.64 | 1.15 |
| Rab32 | Meta | RAB32, member RAS oncogene family | 2.31 | 2.01 | 2.07 |
| Rad51 | Meta | RAD51 homolog (RecA homolog, E. coli) (S. cerevisiae) | 2.70 | 1.36 | -1.21 |
| Rad54l | Meta | RAD54-like (S. cerevisiae) | 2.13 | 1.20 | -1.17 |
| Rag1 | Meta | recombination activating gene 1 | 1.06 | -1.69 | -2.68 |
| Rangap1 | Meta | Ran GTPase activating protein 1 | 2.07 | 1.87 | 1.33 |
| Rasgrp4 | Meta | RAS guanyl releasing protein 4 | 2.40 | 2.04 | 1.71 |
| Rfc4 | Meta | replication factor C (activator 1) 4, 37kDa | 2.36 | 1.09 | -1.01 |
| Rgs1 | Meta | regulator of G-protein signaling 1 | 3.90 | 2.08 | 3.09 |
| Rgs18 | Meta | regulator of G-protein signaling 18 | 3.95 | 1.44 | 1.93 |
| Rhag | Meta | Rh-associated glycoprotein | 3.45 | -1.05 | -1.92 |
| Rnase17 | Meta | ribonuclease 17 | 2.17 | 1.15 | -1.12 |
| Rnase2 | Meta | ribonuclease 2 | 1.56 | -1.23 | -2.17 |
| Rnasel | Meta | ribonuclease L (2',5'-oligoisoadenylate synthetase-dependent) | 2.08 | 1.50 | 1.14 |
| Rnf213 | Meta | ring finger protein 213 | 2.53 | 2.28 | 1.97 |
| Rrm2 | Meta | ribonucleotide reductase M2 | 3.84 | 2.56 | 1.09 |
| Rtn4ip1 | Meta | reticulon 4 interacting protein 1 | 2.06 | 1.86 | 1.29 |
| Sgcg | Meta | sarcoglycan, gamma (35kDa dystrophin-associated glycoprotein) | 3.98 | 2.42 | 3.34 |
| Sh3bgrl3 | Meta | SH3 domain binding glutamic acid-rich protein like 3 | 2.31 | 2.28 | 2.05 |
| Skap2 | Meta | src kinase associated phosphoprotein 2 | 2.08 | 2.11 | 1.96 |
| Slc30a1 | Meta | solute carrier family 30 (zinc transporter), member 1 | 2.42 | 2.38 | 1.84 |
| Sp100 | Meta | SP100 nuclear antigen | 2.36 | 1.91 | 1.94 |
| Spag5 | Meta | sperm associated antigen 5 | 2.55 | 1.62 | -1.17 |
| St8sia4 | Meta | ST8 alpha-N-acetyl-neuraminide alpha-2,8-sialyltransferase 4 | 2.64 | 2.20 | 1.66 |
| Stfa2l3 | Meta | stefin A2-like 3 | 9.77 | 1.43 | -1.33 |
| Synj1 | Meta | synaptojanin 1 | 2.01 | 2.06 | 1.82 |
| Sytl2 | Meta | synaptotagmin-like 2 | 2.24 | 1.86 | 1.92 |
| Sytl3 | Meta | synaptotagmin-like 3 | 1.21 | 2.12 | -1.05 |
| Tfec | Meta | transcription factor EC | 2.38 | 2.09 | 1.54 |
| Timeless | Meta | timeless homolog (Drosophila) | 2.25 | 1.44 | 1.03 |
| Top2a | Meta | topoisomerase (DNA) II alpha 170kDa | 2.57 | 1.42 | -1.22 |
| Trip13 | Meta | thyroid hormone receptor interactor 13 | 2.26 | 1.54 | 1.06 |
| Tuba1a | Meta | tubulin, alpha 1a | 2.73 | 2.65 | 2.63 |
| Ube2l6 | Meta | ubiquitin-conjugating enzyme E2L 6 | 2.11 | 1.84 | 1.56 |
| Ugt1a5 | Meta | UDP glucuronosyltransferase 1 family, polypeptide A5 | 2.45 | 2.40 | 1.86 |
| Uhrf1 | Meta | ubiquitin-like with PHD and ring finger domains 1 | 2.21 | 1.40 | -1.29 |
| Wdr62 | Meta | WD repeat domain 62 | 2.93 | 2.32 | 1.68 |
| Arhgap11a | Other | Rho GTPase activating protein 11A | 2.45 | 1.79 | 1.41 |
| Bach1 | Other | BTB and CNC homology 1, basic leucine zipper transcription factor 1 | 2.13 | 2.22 | 2.02 |
| Bard1 | Other | BRCA1 associated RING domain 1 | 2.26 | 1.40 | -1.21 |
| Baz1a | Other | bromodomain adjacent to zinc finger domain, 1A | 2.32 | 1.99 | 1.75 |
| Bin2 | Other | bridging integrator 2 | 2.12 | 1.68 | 1.30 |
| Casc5 | Other | cancer susceptibility candidate 5 | 2.69 | 1.51 | -1.05 |
| Cep55 | Other | centrosomal protein 55kDa | 2.67 | 1.77 | 1.29 |
| Cit | Other | citron (rho-interacting, serine/threonine kinase 21) | 1.08 | -1.47 | -2.18 |
| Ddx59 | Other | DEAD (Asp-Glu-Ala-Asp) box polypeptide 59 | 2.02 | 1.24 | 1.02 |
| Dem1 | Other | defects in morphology 1 homolog (S. cerevisiae) | 2.28 | 1.59 | 1.45 |
| Depdc1 | Other | DEP domain containing 1 | 2.31 | 1.68 | 1.11 |
| Depdc1b | Other | DEP domain containing 1B | 2.52 | 1.77 | -1.07 |
| Dmxl2 | Other | Dmx-like 2 | 2.87 | 2.16 | 1.70 |
| Dpy19l1 | Other | dpy-19-like 1 (C. elegans) | 2.19 | 1.88 | 2.06 |
| Dsn1 | Other | DSN1, MIND kinetochore complex component, homolog (S. cerevisiae) | 2.06 | 1.32 | 1.02 |
| Dtna | Other | dystrobrevin, alpha | 1.01 | -2.49 | -1.91 |
| Dtx4 | Other | deltex homolog 4 (Drosophila) | 2.54 | 1.81 | 2.20 |
| Epha3 | Other | EPH receptor A3 | 9.61 | 1.26 | 2.03 |
| Evi2a | Other | ecotropic viral integration site 2A | 2.10 | 1.99 | 1.77 |
| Fam105a | Other | family with sequence similarity 105, member A | 6.30 | 3.99 | 4.59 |
| Fbl | Other | fibrillarin | 2.01 | 1.71 | 1.06 |
| Fign | Other | fidgetin | 1.06 | -2.48 | -2.50 |
| Gen1 | Other | Gen homolog 1, endonuclease (Drosophila) | 2.78 | 1.53 | 1.07 |
| Heatr1 | Other | HEAT repeat containing 1 | 2.24 | 2.13 | 1.48 |
| Hjurp | Other | Holliday junction recognition protein | 2.43 | 1.50 | 1.00 |
| Htatip2 | Other | HIV-1 Tat interactive protein 2, 30kDa | 2.42 | 2.31 | 1.98 |
| Inpp5d | Other | inositol polyphosphate-5-phosphatase, 145kDa | 2.27 | 2.33 | 1.64 |
| Iqgap3 | Other | IQ motif containing GTPase activating protein 3 | 2.47 | 1.68 | 1.16 |
| Lgi2 | Other | leucine-rich repeat LGI family, member 2 | 2.87 | 1.36 | -1.00 |
| Lpxn | Other | leupaxin | 2.66 | 2.80 | 2.41 |
| Ly86 | Other | lymphocyte antigen 86 | 2.91 | 1.82 | 1.27 |
| Lyn | Other | v-yes-1 Yamaguchi sarcoma viral related oncogene homolog | 2.43 | 2.48 | 2.00 |
| Magoh | Other | mago-nashi homolog, proliferation-associated (Drosophila) | 2.44 | 2.11 | 1.31 |
| Melk | Other | maternal embryonic leucine zipper kinase | 2.13 | 1.61 | 1.07 |
| Mki67 | Other | antigen identified by monoclonal antibody Ki-67 | 2.35 | 1.45 | -1.31 |
| Ms4a11 | Other | membrane-spanning 4-domains, subfamily A, member 11 | 2.86 | 2.04 | 1.75 |
| Ms4a3 | Other | membrane-spanning 4-domains, subfamily A, member 3 (hematopoietic cell-specific) | 1.42 | -1.59 | -2.69 |
| Ms4a6b | Other | membrane-spanning 4-domains, subfamily A, member 6B | 2.22 | 1.35 | 1.41 |
| Ncapd2 | Other | non-SMC condensin I complex, subunit D2 | 2.19 | 1.33 | -1.35 |
| Nckap1l | Other | NCK-associated protein 1-like | 3.23 | 3.40 | 2.63 |
| Nipsnap3a | Other | nipsnap homolog 3A (C. elegans) | 2.25 | 2.32 | 1.66 |
| Ns5atp9 | Other | NS5A (hepatitis C virus) transactivated protein 9 | 2.17 | 1.36 | -1.94 |
| Penk1 | Other | proenkephalin | 1.19 | -2.63 | -2.23 |
| Plac8 | Other | placenta-specific 8 | 1.47 | -1.30 | -3.30 |
| Plk1 | Other | polo-like kinase 1 (Drosophila) | 2.78 | 2.11 | -1.12 |
| Pram1 | Other | PML-RARA regulated adaptor molecule 1 | 2.02 | 1.28 | -1.45 |
| Prr11 | Other | proline rich 11 | 2.26 | 1.85 | 1.12 |
| Rapgef5 | Other | Rap guanine nucleotide exchange factor (GEF) 5 | 2.55 | 1.41 | 1.69 |
| Samsn1 | Other | SAM domain, SH3 domain and nuclear localization signals 1 | 2.65 | 1.88 | 1.84 |
| Satb1 | Other | SATB homeobox 1 | 1.00 | -1.94 | -2.89 |
| Satb2 | Other | SATB homeobox 2 | 1.00 | -2.21 | -1.34 |
| Sdf2l1 | Other | stromal cell-derived factor 2-like 1 | 3.00 | 2.33 | 2.27 |
| Sgol1 | Other | shugoshin-like 1 (S. pombe) | 2.13 | 1.33 | 1.13 |
| Sla | Other | Src-like-adaptor | 2.63 | 1.57 | 1.05 |
| Slfn8 | Other | schlafen 8 | 2.93 | 2.38 | 2.28 |
| Spib | Other | Spi-B transcription factor (Spi-1/PU.1 related) | -1.10 | -1.90 | -3.69 |
| Stfa2 | Other | stefin A2-like 3 | 2.91 | 1.26 | -1.18 |
| Stil | Other | SCL/TAL1 interrupting locus | 2.64 | 1.59 | 1.07 |
| Stmn2 | Other | stathmin-like 2 | 2.07 | 1.24 | 1.63 |
| Tacc3 | Other | transforming, acidic coiled-coil containing protein 3 | 2.71 | 1.92 | 1.05 |
| Tmem176a | Other | transmembrane protein 176A | 2.77 | 1.95 | 2.06 |
| Tmem176b | Other | transmembrane protein 176B | 2.04 | 1.71 | 1.52 |
| Tmem49 | Other | transmembrane protein 49 | 2.22 | 1.97 | 2.06 |
| Tmsb10 | Other | thymosin beta 10 | 2.04 | 2.13 | 1.54 |
| Tmtc4 | Other | transmembrane and tetratricopeptide repeat containing 4 | 2.93 | 1.56 | 1.48 |
| Ttk | Other | TTK protein kinase | 3.38 | 1.86 | 1.11 |
| Unc93b1 | Other | unc-93 homolog B1 (C. elegans) | 2.73 | 1.88 | 1.80 |
| Whsc1 | Other | Wolf-Hirschhorn syndrome candidate 1 | 2.27 | 1.60 | 1.28 |
| Zdhhc13 | Other | zinc finger, DHHC-type containing 13 | 2.02 | 2.04 | 1.64 |
| Abca3 | Transporter | ATP-binding cassette, sub-family A (ABC1), member 3 | 2.04 | 1.90 | 1.88 |
| Abca8a | Transporter | ATP-binding cassette, sub-family A (ABC1), member 8a | 1.17 | -2.84 | -1.54 |
| Abcb10 | Transporter | ATP-binding cassette, sub-family B (MDR/TAP), member 10 | 2.01 | 1.31 | -1.06 |
| Apoe | Transporter | apolipoprotein E | 3.14 | 2.64 | 2.85 |
| Atp10d | Transporter | ATPase, class V, type 10D | 2.75 | 2.22 | 1.84 |
| Atp5l | Transporter | ATP synthase, H+ transporting, mitochondrial Fo complex, subunit G | 2.21 | 2.18 | 1.50 |
| Atp8b4 | Transporter | ATPase, class I, type 8B, member 4 | 2.37 | 1.57 | 1.24 |
| Cdh17 | Transporter | cadherin 17, LI cadherin (liver-intestine) | 3.06 | 2.85 | 1.81 |
| Heph | Transporter | hephaestin | 2.62 | 1.42 | 1.86 |
| Hmha1 | Transporter | histocompatibility (minor) HA-1 | 2.36 | 2.34 | 1.48 |
| Kcna3 | Transporter | potassium voltage-gated channel, shaker-related subfamily, member 3 | 2.09 | 1.81 | 1.33 |
| Kif20a | Transporter | kinesin family member 20A | 2.63 | 1.93 | 1.21 |
| Kpna2 | Transporter | karyopherin alpha 2 (RAG cohort 1, importin alpha 1) | 2.94 | 2.59 | 1.36 |
| Mfsd1 | Transporter | major facilitator superfamily domain containing 1 | 2.25 | 2.41 | 2.02 |
| Nrxn1 | Transporter | neurexin 1 | 2.93 | 1.42 | 1.38 |
| Nup210 | Transporter | nucleoporin 210kDa | 1.67 | -1.00 | -2.10 |
| Racgap1 | Transporter | Rac GTPase activating protein 1 | 2.48 | 1.81 | 1.06 |
| Shroom2 | Transporter | shroom family member 2 | 1.06 | -2.23 | -2.69 |
| Slc10a6 | Transporter | solute carrier family 10 (sodium/bile acid cotransporter family), member 6 | 2.16 | -1.10 | 1.58 |
| Slc11a1 | Transporter | solute carrier family 11 (proton-coupled divalent metal ion transporters), member 1 | 3.51 | 2.87 | 2.36 |
| Slc15a3 | Transporter | solute carrier family 15, member 3 | 4.14 | 3.23 | 2.44 |
| Slc16a10 | Transporter | solute carrier family 16, member 10 (aromatic amino acid transporter) | 1.22 | -1.26 | -2.80 |
| Slc25a37 | Transporter | solute carrier family 25, member 37 | 2.61 | 1.56 | -1.11 |
| Slc25a5 | Transporter | solute carrier family 25 (mitochondrial carrier; adenine nucleotide translocator), member 6 | 2.83 | 2.91 | 1.95 |
| Slc28a2 | Transporter | solute carrier family 28 (sodium-coupled nucleoside transporter), member 2 | 3.33 | 3.61 | 3.32 |
| Slc9a7 | Transporter | solute carrier family 9 (sodium/hydrogen exchanger), member 7 | 2.21 | 1.95 | 1.68 |
| Slco2a1 | Transporter | solute carrier organic anion transporter family, member 2A1 | 2.68 | 2.45 | 1.87 |
| Tap2 | Transporter | transporter 2, ATP-binding cassette, sub-family B (MDR/TAP) | 2.15 | 2.27 | 1.80 |
