# Supplementary material for: Sequential Alterations in Catabolic and Anabolic Gene Expression Parallel Pathological Changes during Progression of Monoiodoacetate-Induced Arthritis
Source: PLoS One. 2011 Sep 13;6(9):e24320. doi: 10.1371/journal.pone.0024320 (PMC3172226; doi:10.1371/journal.pone.0024320)
Supplement: Table S2 — Changes in the expression of genes in Cluster IV. Please see Table S1 for group description. (DOC) [file pone.0024320.s003.doc]

**Table S2**. Changes in the expression of genes in *Cluster IV*. Please see Table S1 for group description.

| Gene | Group | Description | MIA5 | MIA9 | MIA21 |
| --- | --- | --- | --- | --- | --- |
| Agtr2 | CD | angiotensin II receptor, type 2 | -2.14 | -1.64 | -2.19 |
| Bcl2 | CD | B-cell CLL/lymphoma 2 | -3.29 | -2.26 | -2.28 |
| Bcl6 | CD | B-cell CLL/lymphoma 6 | -4.03 | -3.08 | -2.62 |
| Bnip3 | CD | BCL2/adenovirus E1B 19kDa interacting protein 3 | -5.98 | -2.88 | -1.64 |
| Cidea | CD | cell death-inducing DFFA-like effector a | -2.66 | -1.77 | -2.02 |
| Foxa2 | CD | forkhead box A2 | -3.06 | -2.56 | -2.56 |
| Kif21a | CD | kinesin family member 21A | -7.33 | -6.72 | -4.28 |
| Phlda1 | CD | pleckstrin homology-like domain, family A, member 1 | -2.83 | -1.71 | -2.52 |
| Ptpn3 | CD | protein tyrosine phosphatase, non-receptor type 3 | -5.40 | -4.14 | -2.29 |
| Qsox1 | CD | quiescin Q6 sulfhydryl oxidase 1 | -2.26 | -1.66 | -1.34 |
| S100b | CD | S100 calcium binding protein B | -4.89 | -3.85 | -1.86 |
| Scrg1 | CD | stimulator of chondrogenesis 1 | -61.65 | -53.89 | -12.20 |
| Tspan2 | CD | tetraspanin 2 | -2.45 | 1.42 | 2.59 |
| Acan | ECM | aggrecan | -4.12 | -3.30 | -2.05 |
| Bgn | ECM | biglycan | -2.12 | -1.69 | 1.07 |
| Chrdl2 | ECM | chordin-like 2 | -6.75 | -6.69 | -7.28 |
| Cilp | ECM | cartilage intermediate layer protein, nucleotide pyrophosphohydrolase | -92.28 | -66.21 | -23.19 |
| Cilp2 | ECM | cartilage intermediate layer protein 2 | -21.89 | -19.15 | -3.31 |
| Col14a1 | ECM | collagen, type XIV, alpha 1 | -2.07 | -1.19 | 3.92 |
| Col15a1 | ECM | collagen, type XV, alpha 1 | -1.91 | 1.27 | 2.01 |
| Col16a1 | ECM | collagen, type XVI, alpha 1 | -2.69 | -1.53 | -1.29 |
| Col27a1 | ECM | collagen, type XXVII, alpha 1 | -4.57 | -3.24 | -3.30 |
| Col6a1 | ECM | collagen, type VI, alpha 1 | -1.33 | 1.60 | 2.01 |
| Col6a2 | ECM | collagen, type VI, alpha 2 | -1.31 | 1.71 | 2.15 |
| Eln | ECM | elastin | -2.59 | 1.05 | 1.51 |
| Emilin3 | ECM | elastin microfibril interfacer 3 | -2.67 | -1.96 | -1.27 |
| Fbln7 | ECM | fibulin 7 | -13.34 | -9.93 | -3.72 |
| Fmod | ECM | fibromodulin | -13.10 | -11.93 | -1.75 |
| Hapln3 | ECM | hyaluronan and proteoglycan link protein 3 | -6.50 | -6.13 | -6.03 |
| Kera | ECM | keratocan | -1.93 | 1.15 | 2.67 |
| Leprel2 | ECM | leprecan-like 2 | -2.06 | -1.77 | -1.35 |
| Ntn1 | ECM | netrin 1 | -7.90 | -3.12 | -1.82 |
| Ntng1 | ECM | netrin G1 | -2.36 | -2.19 | -1.77 |
| Prelp | ECM | proline/arginine-rich end leucine-rich repeat protein | -6.04 | -5.62 | -1.42 |
| Sdc4 | ECM | syndecan 4 | -2.27 | -1.01 | -1.36 |
| Spock1 | ECM | sparc/osteonectin, cwcv and kazal-like domains proteoglycan (testican) 1 | -3.01 | -2.76 | -3.02 |
| Spon2 | ECM | spondin 2, extracellular matrix protein | -2.89 | 2.23 | 1.73 |
| Tnxb | ECM | tenascin XB | -2.37 | -2.23 | 1.84 |
| Chst11 | ECM2 | carbohydrate (chondroitin 4) sulfotransferase 11 | -4.71 | -4.25 | -4.88 |
| Chst3 | ECM2 | carbohydrate (chondroitin 6) sulfotransferase 3 | -10.82 | -7.17 | -4.65 |
| Cspg4 | ECM2 | chondroitin sulfate proteoglycan 4 | -3.07 | -2.72 | -1.71 |
| Hs6st2 | ECM2 | heparan sulfate 6-O-sulfotransferase 2 | -2.07 | -1.91 | -2.02 |
| Loxl2 | ECM2 | lysyl oxidase-like 2 | -2.43 | -1.25 | -1.18 |
| Mmp15 | ECM2 | matrix metallopeptidase 15 (membrane-inserted) | -1.16 | 1.27 | 2.22 |
| Pcdh9 | ECM2 | protocadherin 9 | -3.08 | -2.64 | -2.72 |
| Pi15 | ECM2 | peptidase inhibitor 15 | -18.00 | -17.90 | -4.75 |
| Reck | ECM2 | reversion-inducing-cysteine-rich protein with kazal motifs | -3.13 | -2.83 | -1.74 |
| Sepp1 | ECM2 | selenoprotein P, plasma, 1 | -2.72 | -2.47 | -2.20 |
| Serpina3n | ECM2 | serpin peptidase inhibitor, clade A (alpha-1 antiproteinase, antitrypsin), member 3 | -10.80 | -1.85 | -1.62 |
| Sulf2 | ECM2 | sulfatase 2 | -2.44 | 1.06 | 1.45 |
| Timp3 | ECM2 | TIMP metallopeptidase inhibitor 3 | -2.87 | 1.14 | 1.31 |
| Bmp6 | GF | bone morphogenetic protein 6 | -5.96 | -5.40 | -3.56 |
| Gdf10 | GF | growth differentiation factor 10 | -22.56 | -16.64 | -4.89 |
| Igf2 | GF | insulin-like growth factor 2 (somatomedin A) | -7.05 | -6.48 | -4.87 |
| Ltbp4 | GF | latent transforming growth factor beta binding protein 4 | -2.86 | -1.68 | 1.22 |
| Vegfa | GF | vascular endothelial growth factor A | -2.57 | -1.13 | -1.09 |
| Crim1 | GF2 | cysteine rich transmembrane BMP regulator 1 (chordin-like) | -12.66 | -10.76 | -8.59 |
| Dkk3 | GF2 | dickkopf homolog 3 (Xenopus laevis) | -1.25 | 1.29 | 4.04 |
| Fgfrl1 | GF2 | fibroblast growth factor receptor-like 1 | -5.06 | -4.63 | -3.44 |
| Flrt1 | GF2 | fibronectin leucine rich transmembrane protein 1 | -2.70 | -2.46 | -2.75 |
| Fzd8 | GF2 | frizzled homolog 8 (Drosophila) | -3.15 | -2.15 | 1.09 |
| Htra3 | GF2 | HtrA serine peptidase 3 | -2.54 | -1.59 | -1.55 |
| Insig1 | GF2 | insulin induced gene 1 | -1.09 | 2.65 | 1.85 |
| Samd4a | GF2 | sterile alpha motif domain containing 4A | -3.65 | -2.37 | -2.06 |
| Smoc2 | GF2 | SPARC related modular calcium binding 2 | -3.71 | -2.27 | -1.58 |
| Sox5 | GF2 | SRY-box containing gene 5 | -5.11 | -4.14 | -2.24 |
| Sox9 | GF2 | SRY (sex determining region Y)-box 9 | -10.15 | -9.71 | -5.59 |
| Cytl1 | Inf | cytokine-like 1 | -53.63 | -47.51 | -49.75 |
| Il23r | Inf | interleukin 23 receptor | -2.56 | -2.12 | -1.47 |
| Il34 | Inf | interleukin 34 | -1.24 | -1.12 | 2.04 |
| Tnfrsf11b | Inf | tumor necrosis factor receptor superfamily, member 11b | -9.03 | -8.75 | -4.40 |
| Agt | Inf2 | angiotensinogen (serpin peptidase inhibitor, clade A, member 8) | -8.49 | -7.93 | -6.58 |
| Alox12 | Inf2 | arachidonate 12-lipoxygenase | -2.53 | -2.26 | -2.77 |
| Arhgap8 | Inf2 | Rho GTPase activating protein 8 | -4.67 | -4.34 | -4.61 |
| Atf5 | Inf2 | activating transcription factor 5 | -2.00 | -1.64 | -1.04 |
| C1qtnf1 | Inf2 | C1q and tumor necrosis factor related protein 1 | -2.41 | -2.14 | -1.39 |
| C1qtnf2 | Inf2 | C1q and tumor necrosis factor related protein 2 | -4.09 | -2.56 | -1.32 |
| Etv4 | Inf2 | ets variant 4 | -2.19 | -1.66 | -1.83 |
| Etv5 | Inf2 | ets variant 5 | -2.39 | -2.02 | -1.45 |
| Lrba | Inf2 | LPS-responsive vesicle trafficking, beach and anchor containing | -2.19 | -1.83 | -1.43 |
| Mall | Inf2 | mal, T-cell differentiation protein-like | -4.17 | -2.05 | -1.46 |
| Nfatc2 | Inf2 | nuclear factor of activated T-cells, cytoplasmic, calcineurin-dependent 2 | -2.39 | -1.03 | 1.08 |
| Nos2 | Inf2 | nitric oxide synthase 2, inducible | -1.13 | 2.11 | 1.66 |
| Otud7b | Inf2 | OTU domain containing 7B | -2.01 | -1.73 | -1.27 |
| Pla1a | Inf2 | phospholipase A1 member A | -2.36 | -1.31 | 1.34 |
| Plat | Inf2 | plasminogen activator, tissue | -1.47 | 2.01 | 1.73 |
| Ptgds | Inf2 | prostaglandin D2 synthase 21kDa (brain) | -2.09 | -2.08 | -2.00 |
| Rab33b | Inf2 | RAB33B, member RAS oncogene family | -2.04 | -1.79 | -1.43 |
| Sod3 | Inf2 | superoxide dismutase 3, extracellular | -7.41 | -3.89 | -2.43 |
| Tob1 | Inf2 | transducer of ERBB2, 1 | -2.03 | -1.51 | 1.02 |
| Vwce | Inf2 | von Willebrand factor C and EGF domains | -3.33 | -3.31 | -3.35 |
| Abtb2 | Meta | ankyrin repeat and BTB (POZ) domain containing 2 | -4.20 | -2.89 | -2.07 |
| Ace | Meta | angiotensin I converting enzyme (peptidyl-dipeptidase A) 1 | -6.03 | -5.42 | -4.09 |
| Acsl6 | Meta | acyl-CoA synthetase long-chain family member 6 | -3.42 | -3.30 | -3.43 |
| Actn2 | Meta | actinin, alpha 2 | -3.62 | -2.86 | -2.80 |
| Adm2 | Meta | adrenomedullin 2 | -2.28 | -2.26 | -1.92 |
| Ak3l1 | Meta | adenylate kinase 3-like 1 | -5.09 | -3.57 | -2.47 |
| Aldoc | Meta | aldolase C, fructose-bisphosphate | -6.40 | -5.29 | -3.26 |
| Angptl1 | Meta | angiopoietin-like 1 | -4.06 | -2.55 | 1.66 |
| Aox1 | Meta | aldehyde oxidase 1 | -3.82 | -3.13 | -1.04 |
| Apobec2 | Meta | apolipoprotein B mRNA editing enzyme, catalytic polypeptide-like 2 | -5.26 | -4.85 | -4.08 |
| Apol9a | Meta | apolipoprotein L 9a | -1.33 | 1.55 | 2.20 |
| Arc | Meta | activity-regulated cytoskeleton-associated protein | -3.06 | -2.80 | -2.17 |
| Bace2 | Meta | beta-site APP-cleaving enzyme 2 | -2.19 | -1.77 | -1.10 |
| Begain | Meta | brain-enriched guanylate kinase-associated homolog (rat) | -3.44 | -3.13 | -3.44 |
| Camk4 | Meta | calcium/calmodulin-dependent protein kinase IV | -5.19 | -4.81 | -5.42 |
| Car5b | Meta | carbonic anhydrase VB, mitochondrial | -3.58 | -1.34 | 1.12 |
| Car9 | Meta | carbonic anhydrase IX | -5.13 | -2.47 | -1.55 |
| Casr | Meta | calcium-sensing receptor | -14.96 | -5.77 | -3.38 |
| Cercam | Meta | cerebral endothelial cell adhesion molecule | -4.06 | -3.48 | -2.14 |
| Clstn3 | Meta | calsyntenin 3 | -3.23 | -2.01 | -2.56 |
| Cox4i2 | Meta | cytochrome c oxidase subunit IV isoform 2 (lung) | -10.98 | -4.02 | -2.65 |
| Cox6a2 | Meta | cytochrome c oxidase subunit VIa polypeptide 2 | -2.35 | -2.18 | -2.05 |
| Cybrd1 | Meta | cytochrome b reductase 1 | -2.63 | -2.33 | -1.38 |
| Cyp26b1 | Meta | cytochrome P450, family 26, subfamily B, polypeptide 1 | -3.35 | -1.29 | 1.03 |
| Dcc | Meta | deleted in colorectal carcinoma | -2.17 | -1.95 | -1.97 |
| Dmd | Meta | dystrophin | -2.12 | -1.82 | -1.42 |
| Dusp1 | Meta | dual specificity phosphatase 1 | -3.56 | -2.09 | -1.18 |
| Dusp14 | Meta | dual specificity phosphatase 14 | -2.81 | -2.23 | -1.24 |
| Egln1 | Meta | egl nine homolog 1 (C. elegans) | -2.36 | -1.53 | -1.38 |
| Egln3 | Meta | egl nine homolog 3 (C. elegans) | -3.32 | -1.62 | 1.45 |
| Eno2 | Meta | enolase 2 (gamma, neuronal) | -2.31 | -1.27 | -1.03 |
| Enpep | Meta | glutamyl aminopeptidase (aminopeptidase A) | -1.23 | 1.22 | 2.19 |
| Enpp2 | Meta | ectonucleotide pyrophosphatase/phosphodiesterase 2 | -5.53 | -3.49 | -1.59 |
| Fbp2 | Meta | fructose-1,6-bisphosphatase 2 | -12.78 | -10.71 | -10.33 |
| Gale | Meta | UDP-galactose-4-epimerase | -2.37 | -2.12 | -2.60 |
| Galntl2 | Meta | UDP-N-acetyl-alpha-D-galactosamine:polypeptide N-acetylgalactosaminyltransferase-like 2 | -2.95 | -2.53 | -1.67 |
| Galntl4 | Meta | UDP-N-acetyl-alpha-D-galactosamine:polypeptide N-acetylgalactosaminyltransferase-like 4 | -5.06 | -4.27 | -2.07 |
| Gpld1 | Meta | glycosylphosphatidylinositol specific phospholipase D1 | -3.51 | -3.02 | -2.68 |
| Gsn | Meta | gelsolin (amyloidosis, Finnish type) | -3.02 | -2.29 | -1.43 |
| Htra4 | Meta | HtrA serine peptidase 4 | -10.02 | -4.49 | 2.18 |
| Kras | Meta | v-Ki-ras2 Kirsten rat sarcoma viral oncogene homolog | -2.46 | -2.10 | -1.87 |
| Ldhb | Meta | lactate dehydrogenase B | -2.21 | -2.05 | -1.76 |
| Ldlrad3 | Meta | low density lipoprotein receptor class A domain containing 3 | -3.12 | -2.98 | -2.61 |
| Lipg | Meta | lipase, endothelial | -9.55 | -6.25 | -1.95 |
| Lrp1 | Meta | low density lipoprotein-related protein 1 (alpha-2-macroglobulin receptor) | -2.31 | -1.14 | 1.09 |
| Map2 | Meta | microtubule-associated protein 2 | -2.65 | -2.59 | -2.44 |
| Mapre3 | Meta | microtubule-associated protein, RP/EB family, member 3 | -2.06 | -1.73 | -1.33 |
| Me1 | Meta | malic enzyme 1, NADP(+)-dependent, cytosolic | -2.93 | -1.56 | -1.56 |
| Mfi2 | Meta | antigen p97 (melanoma associated) identified by monoclonal antibodies 133.2 and 96.5 | -31.16 | -30.92 | -15.73 |
| Mid2 | Meta | midline 2 | -2.00 | -1.73 | -1.12 |
| Mtmr7 | Meta | myotubularin related protein 7 | -2.57 | -2.30 | -1.83 |
| Myh7 | Meta | myosin, heavy chain 7, cardiac muscle, beta | -4.32 | -4.10 | -3.74 |
| Myoc | Meta | myocilin, trabecular meshwork inducible glucocorticoid response | -58.34 | -38.13 | -18.75 |
| Ndrg1 | Meta | N-myc downstream regulated 1 | -1.03 | 1.57 | 2.27 |
| Ngef | Meta | neuronal guanine nucleotide exchange factor | -4.65 | -3.58 | -2.32 |
| Nid2 | Meta | nidogen 2 (osteonidogen) | -2.07 | -1.19 | -1.46 |
| Nt5e | Meta | 5'-nucleotidase, ecto (CD73) | -4.68 | -3.94 | -1.62 |
| Osbpl5 | Meta | oxysterol binding protein-like 5 | -2.74 | -2.54 | -2.25 |
| P4ha1 | Meta | prolyl 4-hydroxylase, alpha polypeptide I | -2.24 | -1.74 | -1.21 |
| P4ha2 | Meta | prolyl 4-hydroxylase, alpha polypeptide II | -3.27 | -1.87 | -1.16 |
| Pacsin3 | Meta | protein kinase C and casein kinase substrate in neurons 3 | -2.06 | -1.89 | -1.65 |
| Pam | Meta | peptidylglycine alpha-amidating monooxygenase | -4.93 | -4.54 | -2.75 |
| Pank1 | Meta | pantothenate kinase 1 | -2.90 | -2.22 | -2.50 |
| Papss2 | Meta | 3'-phosphoadenosine 5'-phosphosulfate synthase 2 | -3.76 | -2.98 | -2.40 |
| Pcdh11x | Meta | protocadherin 11 Y-linked | -2.14 | -1.98 | -1.70 |
| Pde11a | Meta | phosphodiesterase 11A | -3.22 | -2.71 | -2.32 |
| Pdia5 | Meta | protein disulfide isomerase family A, member 5 | -2.01 | 1.01 | -2.15 |
| Pfkfb3 | Meta | 6-phosphofructo-2-kinase/fructose-2,6-biphosphatase 3 | -3.15 | -2.05 | -1.17 |
| Prkar1b | Meta | protein kinase, cAMP-dependent, regulatory, type I, beta | -2.04 | -1.92 | -1.83 |
| Rarg | Meta | retinoic acid receptor, gamma | -2.64 | -2.45 | -1.95 |
| Rerg | Meta | RAS-like, estrogen-regulated, growth inhibitor | -3.77 | -2.02 | 1.46 |
| Shroom4 | Meta | shroom family member 4 | -2.62 | -2.40 | -1.79 |
| Smox | Meta | spermine oxidase | -1.39 | -1.06 | -2.01 |
| Synm | Meta | synemin, intermediate filament protein | -2.04 | -1.37 | 1.19 |
| Tmprss2 | Meta | transmembrane protease, serine 2 | -2.03 | -1.86 | -2.00 |
| Tnni1 | Meta | troponin I type 1 (skeletal, slow) | -2.33 | -2.17 | -2.16 |
| Tnni2 | Meta | troponin I type 2 (skeletal, fast) | -13.63 | -9.09 | -8.35 |
| Xylt1 | Meta | xylosyltransferase I | -8.96 | -8.13 | -4.30 |
| Ankrd15 | Other | KN motif and ankyrin repeat domains 1 | -2.48 | -2.08 | -1.94 |
| Ankrd37 | Other | ankyrin repeat domain 37 | -2.04 | -1.13 | -1.04 |
| Anxa11 | Other | annexin A11 | -2.12 | -1.51 | -1.43 |
| Anxa8 | Other | annexin A8 | -4.10 | -2.25 | -1.35 |
| Arhgef1 | Other | Rho guanine nucleotide exchange factor (GEF) 1 | -1.81 | -1.70 | -2.13 |
| Asb15 | Other | ankyrin repeat and SOCS box-containing 15 | -6.13 | -2.31 | -3.36 |
| Axin2 | Other | axin 2 | -2.02 | -1.93 | -1.61 |
| Baiap2l1 | Other | BAI1-associated protein 2-like 1 | -7.17 | -4.90 | -3.64 |
| Barx1 | Other | BARX homeobox 1 | -3.46 | -2.92 | -2.33 |
| Boc | Other | Boc homolog (mouse) | -5.98 | -4.92 | -2.06 |
| Ccdc3 | Other | coiled-coil domain containing 3 | -1.00 | 1.12 | 2.44 |
| Cdon | Other | Cdon homolog (mouse) | -4.01 | -3.35 | -1.45 |
| Clu | Other | clusterin | -4.01 | -3.14 | -1.76 |
| Cobl | Other | cordon-bleu homolog (mouse) | -2.12 | -1.96 | -1.65 |
| Cobll1 | Other | COBL-like 1 | -4.74 | -3.19 | -3.33 |
| Cpne8 | Other | copine VIII | -2.17 | -1.84 | -1.60 |
| Crispld2 | Other | cysteine-rich secretory protein LCCL domain containing 2 | -1.59 | -1.03 | 2.10 |
| Ctxn3 | Other | cortexin 3 | -2.85 | -2.33 | -2.80 |
| Cubn | Other | cubilin (intrinsic factor-cobalamin receptor) | -2.45 | -2.13 | -2.04 |
| Dchs1 | Other | dachsous 1 (Drosophila) | -1.01 | 1.45 | 2.02 |
| Dcx | Other | doublecortin | -2.13 | -2.10 | -2.20 |
| Ddit4l | Other | DNA-damage-inducible transcript 4-like | -3.94 | -3.48 | -2.88 |
| Dennd3 | Other | DENN/MADD domain containing 3 | -2.05 | -1.69 | -2.08 |
| Dixdc1 | Other | DIX domain containing 1 | -3.05 | -2.23 | -1.27 |
| Eml5 | other | echinoderm microtubule associated protein like 5 | -2.32 | -2.13 | -1.95 |
| Emp2 | Other | epithelial membrane protein 2 | -2.14 | -1.11 | 1.08 |
| Eps8l2 | Other | EPS8-like 2 | -11.41 | -9.92 | -6.16 |
| Fam110c | Other | family with sequence similarity 110, member C | -2.36 | -1.89 | -1.99 |
| Fam111a | Other | family with sequence similarity 111, member A | -2.73 | -2.30 | -5.30 |
| FAM120C | Other | family with sequence similarity 120C | -2.17 | -2.10 | -1.69 |
| Fam162a | other | family with sequence similarity 162, member A | -2.30 | -1.56 | -1.44 |
| Fam180a | Other | family with sequence similarity 180, member A | -5.25 | -4.63 | -1.87 |
| Fam70b | Other | family with sequence similarity 70, member B | -2.24 | -1.44 | -1.10 |
| Fibin | Other | fin bud initiation factor homolog (zebrafish) | -7.75 | -4.87 | -3.59 |
| Flnb | Other | filamin B, beta | -2.25 | -1.82 | -1.79 |
| Fuz | Other | fuzzy homolog (Drosophila) | -2.02 | -1.74 | -1.52 |
| Gfra1 | Other | GDNF family receptor alpha 1 | -2.79 | -1.81 | -1.43 |
| Gnas | Other | GNAS complex locus | -4.26 | -3.47 | -2.02 |
| Gpr128 | Other | G protein-coupled receptor 128 | -3.49 | -3.20 | -2.10 |
| Gpr64 | Other | G protein-coupled receptor 64 | -14.50 | -10.33 | -6.97 |
| Higd1a | Other | HIG1 hypoxia inducible domain family, member 1A | -3.15 | -1.80 | -1.21 |
| Hr | Other | hairless homolog (mouse) | -7.57 | -5.16 | -3.17 |
| Hrasls | Other | HRAS-like suppressor | -2.97 | -1.99 | -1.07 |
| Hspb6 | Other | heat shock protein, alpha-crystallin-related, B6 | -2.82 | -2.11 | -2.39 |
| Id4 | Other | inhibitor of DNA binding 4, dominant negative helix-loop-helix protein | -6.88 | -5.42 | -4.16 |
| Igsf10 | Other | immunoglobulin superfamily, member 10 | -1.15 | 1.77 | 2.81 |
| Lgi4 | Other | leucine-rich repeat LGI family, member 4 | -2.65 | -1.50 | -1.40 |
| Lgr6 | Other | leucine-rich repeat-containing G protein-coupled receptor 6 | -5.02 | -3.06 | -4.13 |
| Lmcd1 | Other | LIM and cysteine-rich domains 1 | -6.77 | -4.09 | -5.19 |
| Lrrn2 | Other | leucine rich repeat neuronal 2 | -3.48 | -3.21 | -3.38 |
| Lsamp | Other | limbic system-associated membrane protein | -6.22 | -5.89 | -5.64 |
| Lypd6 | Other | LY6/PLAUR domain containing 6 | -2.09 | -2.07 | -2.00 |
| Mageh1 | Other | melanoma antigen family H, 1 | -2.63 | -2.54 | -1.96 |
| Mamdc2 | Other | MAM domain containing 2 | -1.66 | 2.15 | 3.88 |
| Marveld1 | Other | MARVEL domain containing 1 | -2.73 | -1.21 | -1.22 |
| Mfge8 | Other | milk fat globule-EGF factor 8 protein | -10.43 | -5.36 | -4.65 |
| Mn1 | Other | meningioma (disrupted in balanced translocation) 1 | -3.84 | -3.44 | -2.08 |
| Mtmr10 | Other | myotubularin related protein 10 | -3.89 | -3.78 | -2.85 |
| Mtss1l | Other | metastasis suppressor 1-like | -4.13 | -2.91 | -2.09 |
| Mup4 | Other | major urinary protein 4 | -3.10 | -2.27 | -2.96 |
| Mup5 | Other | major urinary protein 5 | -2.89 | -2.51 | -2.58 |
| Mybpc1 | Other | myosin binding protein C, slow type | -3.87 | -3.68 | -4.42 |
| Myom1 | Other | myomesin 1, 185kDa | -2.76 | -2.38 | -2.20 |
| Myoz2 | Other | myozenin 2 | -2.52 | -2.08 | -2.19 |
| Nfasc | Other | neurofascin homolog (chicken) | -4.47 | -3.81 | -4.03 |
| Nrep | Other | Nrep neuronal regeneration related protein | -5.06 | -3.14 | -2.01 |
| Nupr1 | Other | nuclear protein, transcriptional regulator, 1 | -2.74 | -1.49 | 1.08 |
| Obp3 | Other | alpha-2u globulin PGCL4 | -7.84 | -5.66 | -6.72 |
| Olfml2a | Other | olfactomedin-like 2A | -1.19 | 1.63 | 2.26 |
| Pdk4 | Other | pyruvate dehydrogenase kinase, isozyme 4 | -2.45 | -2.27 | -1.15 |
| Pdzrn4 | Other | PDZ domain containing ring finger 4 | -2.87 | -1.94 | -2.22 |
| Phtf2 | Other | putative homeodomain transcription factor 2 | -2.48 | -1.55 | -1.16 |
| Plac9 | Other | placenta-specific 9 | -2.39 | -2.16 | -1.89 |
| Plbd2 | Other | phospholipase B domain containing 2 | -2.08 | -1.35 | -1.20 |
| Plekhb1 | Other | pleckstrin homology domain containing, family B (evectins) member 1 | -3.85 | -2.37 | -2.37 |
| Plxdc1 | Other | plexin domain containing 1 | -1.12 | 1.62 | 2.92 |
| Plxna4a | Other | plexin A4 | -3.83 | -2.92 | -2.48 |
| Ppap2b | Other | phosphatidic acid phosphatase type 2B | -2.17 | -1.35 | 1.43 |
| Ppp1r1b | Other | protein phosphatase 1, regulatory (inhibitor) subunit 1B | -4.47 | -4.16 | -3.93 |
| Ppp1r3c | Other | protein phosphatase 1, regulatory (inhibitor) subunit 3C | -3.81 | -2.88 | -2.21 |
| Ppp1r9a | Other | protein phosphatase 1, regulatory (inhibitor) subunit 9A | -4.58 | -4.06 | -4.49 |
| Prkg1 | Other | protein kinase, cGMP-dependent, type I | -15.11 | -10.17 | -4.79 |
| Prr5 | Other | proline rich 5 (renal) | -3.89 | -3.57 | -2.86 |
| Ramp2 | Other | receptor (G protein-coupled) activity modifying protein 2 | -2.56 | -2.38 | -2.10 |
| Rasl12 | Other | RAS-like, family 12 | -3.46 | -2.83 | -2.42 |
| Rftn2 | other | raftlin family member 2 | -4.88 | -3.67 | -2.04 |
| Rgp1 | Other | RGP1 retrograde golgi transport homolog (S. cerevisiae) | -27.47 | -7.30 | -13.09 |
| Rgs11 | Other | regulator of G-protein signaling 11 | -4.33 | -3.56 | -3.48 |
| Rimklb | Other | ribosomal modification protein rimK-like family member B | -3.11 | -2.99 | -3.38 |
| Rnf39 | Other | ring finger protein 39 | -3.97 | -3.48 | -3.28 |
| Rora | Other | RAR-related orphan receptor A | -2.41 | -1.56 | 1.05 |
| Rtkn | Other | rhotekin | -2.21 | -2.05 | -1.64 |
| Rundc1 | Other | RUN domain containing 1 | -2.71 | -1.87 | -1.89 |
| S100a1 | Other | S100 calcium binding protein A1 | -2.12 | -2.09 | -1.98 |
| Sdk2 | Other | sidekick homolog 2 (chicken) | -3.95 | -3.46 | -2.38 |
| Sema3a | Other | sema domain, immunoglobulin domain (Ig), short basic domain, secreted, (semaphorin) 3A | -12.47 | -5.61 | -3.46 |
| Sema3d | Other | sema domain, immunoglobulin domain (Ig), short basic domain, secreted, (semaphorin) 3D | -13.54 | -9.15 | -2.03 |
| Sema3e | Other | sema domain, immunoglobulin domain (Ig), short basic domain, secreted, (semaphorin) 3E | -7.63 | -6.84 | -3.47 |
| Sft2d2 | Other | SFT2 domain containing 2 | -2.71 | -2.13 | -1.67 |
| Shc3 | Other | SHC (Src homology 2 domain containing) transforming protein 3 | -1.17 | 1.94 | 3.14 |
| Sipa1l1 | Other | signal-induced proliferation-associated 1 like 1 | -2.42 | -2.41 | -2.18 |
| Smoc1 | Other | SPARC related modular calcium binding 1 | -15.06 | -7.71 | -2.44 |
| Sparcl1 | Other | SPARC-like 1 (hevin) | -1.44 | 1.37 | 2.76 |
| Srpx2 | Other | sushi-repeat-containing protein, X-linked 2 | -2.16 | -1.58 | -1.56 |
| Stk32b | Other | serine/threonine kinase 32B | -4.97 | -4.62 | -4.57 |
| Stk39 | Other | serine threonine kinase 39 (STE20/SPS1 homolog, yeast) | -2.56 | -2.31 | -2.05 |
| Tbx4 | Other | T-box 4 | -2.99 | -2.73 | -2.20 |
| Tbx5 | Other | T-box 5 | -3.10 | -2.63 | -2.54 |
| Tmem106c | Other | transmembrane protein 106C | -2.19 | -1.81 | -1.80 |
| Trim47 | Other | tripartite motif-containing 47 | -2.86 | -1.72 | -1.11 |
| Trps1 | Other | trichorhinophalangeal syndrome I | -2.03 | -1.62 | -1.21 |
| Tspyl4 | Other | TSPY-like 4 | -2.15 | -1.94 | -1.43 |
| Ush1c | Other | Usher syndrome 1C (autosomal recessive, severe) | -2.19 | -1.86 | -2.03 |
| Wwtr1 | Other | WW domain containing transcription regulator 1 | -2.27 | -1.63 | -1.28 |
| Zfp385b | Other | zinc finger protein 385B | -30.40 | -27.78 | -22.10 |
| Zhx2 | Other | zinc fingers and homeoboxes 2 | -2.22 | -1.85 | -1.29 |
| Ano1 | Transporter | anoctamin 1, calcium activated chloride channel | -1.28 | 1.75 | 2.40 |
| Atp6v0a4 | Transporter | ATPase, H+ transporting, lysosomal V0 subunit a4 | -3.22 | -2.88 | -2.51 |
| Clic5 | Transporter | chloride intracellular channel 5 | -4.92 | -3.33 | -1.47 |
| Clstn2 | Transporter | calsyntenin 2 | -7.84 | -5.38 | -6.15 |
| Fam101a | Transporter | family with sequence similarity 101, member A | -4.81 | -3.56 | -3.99 |
| Fxyd1 | Transporter | FXYD domain containing ion transport regulator 1 | -2.61 | -2.27 | -1.34 |
| Gabre | Transporter | gamma-aminobutyric acid (GABA) A receptor, epsilon | -2.28 | -1.75 | -1.52 |
| Igfbp7 | Transporter | insulin-like growth factor binding protein 7 | -3.19 | -2.15 | -1.10 |
| Kcna6 | Transporter | potassium voltage-gated channel, shaker-related subfamily, member 6 | -10.69 | -9.06 | -5.88 |
| Kcnq4 | Transporter | potassium voltage-gated channel, KQT-like subfamily, member 4 | -2.65 | -2.43 | -2.18 |
| Mcoln2 | Transporter | mucolipin 2 | -3.31 | -3.26 | -2.86 |
| Pkd1 | Transporter | polycystic kidney disease 1 (autosomal dominant) | -2.17 | -1.42 | 1.13 |
| Scn2b | Transporter | sodium channel, voltage-gated, type II, beta | -3.37 | -2.38 | -1.59 |
| Scn8a | Transporter | sodium channel, voltage gated, type VIII, alpha subunit | -2.71 | -2.37 | -2.38 |
| Slc10a2 | Transporter | solute carrier family 10 (sodium/bile acid cotransporter family), member 2 | -2.82 | -2.55 | -2.54 |
| Slc16a3 | Transporter | solute carrier family 16, member 3 (monocarboxylic acid transporter 4) | -4.08 | -1.31 | -1.73 |
| Slc16a4 | Transporter | solute carrier family 16, member 4 (monocarboxylic acid transporter 5) | -4.04 | -3.84 | -2.86 |
| Slc1a1 | Transporter | solute carrier family 1 (neuronal/epithelial high affinity glutamate transporter, system Xag), member 1 | -2.58 | -2.12 | -1.99 |
| Slc27a1 | Transporter | solute carrier family 27 (fatty acid transporter), member 1 | -2.73 | -1.75 | -1.51 |
| Slc2a1 | Transporter | solute carrier family 2 (facilitated glucose transporter), member 1 | -3.00 | -1.25 | -1.20 |
| Slc35d1 | Transporter | solute carrier family 35 (UDP-glucuronic acid/UDP-N-acetylgalactosamine dual transporter), member D1 | -2.45 | -2.03 | -1.81 |
| Slc38a1 | Transporter | solute carrier family 38, member 1 | -2.10 | -2.00 | -1.68 |
| Slc4a8 | Transporter | solute carrier family 4, sodium bicarbonate cotransporter, member 8 | -3.28 | -2.98 | -2.96 |
| Slc5a3 | Transporter | solute carrier family 5 (sodium/myo-inositol cotransporter), member 3 | -4.27 | -2.93 | -5.01 |
| Slc6a12 | Transporter | solute carrier family 6 (neurotransmitter transporter, betaine/GABA), member 12 | -2.01 | -1.02 | -1.21 |
| Tom1l2 | Transporter | target of myb1-like 2 (chicken) | -2.13 | -2.00 | -1.62 |
| Trpv4 | Transporter | transient receptor potential cation channel, subfamily V, member 4 | -9.37 | -7.99 | -5.85 |
