# Supplementary material for: Sequential Alterations in Catabolic and Anabolic Gene Expression Parallel Pathological Changes during Progression of Monoiodoacetate-Induced Arthritis
Source: PLoS One. 2011 Sep 13;6(9):e24320. doi: 10.1371/journal.pone.0024320 (PMC3172226; doi:10.1371/journal.pone.0024320)
Supplement: Table S3 — Changes in the expression of genes in Cluster II. Please see Table S1 for group description. (DOC) [file pone.0024320.s004.doc]

**Table S3**. Changes in the expression of genes in *Cluster II*. Please see Table S1 for group description.

| Gene | Group | Description | MIA5 | MIA9 | MIA21 |
| --- | --- | --- | --- | --- | --- |
| Banf1 | CD | barrier to autointegration factor 1 | 1.49 | 2.05 | 1.13 |
| Bcl2a1d | CD | BCL2-related protein A1 | 4.21 | 9.77 | 4.16 |
| Bid | CD | BH3 interacting domain death agonist | 2.36 | 2.98 | 2.31 |
| Birc3 | CD | baculoviral IAP repeat-containing 3 | 1.36 | 2.88 | 1.77 |
| Ccng1 | CD | cyclin G1 | 1.84 | 2.22 | 1.67 |
| Ciapin1 | CD | cytokine induced apoptosis inhibitor 1 | 1.98 | 2.58 | 1.78 |
| Dapk1 | CD | death-associated protein kinase 1 | 2.95 | 4.06 | 3.13 |
| Diaph1 | CD | diaphanous homolog 1 (Drosophila) | 2.09 | 2.60 | 2.13 |
| Dock5 | CD | dedicator of cytokinesis 5 | 2.87 | 4.24 | 3.11 |
| Gprc5a | CD | G protein-coupled receptor, family C, group 5, member A | 2.73 | 3.38 | 2.97 |
| Grn | CD | granulin | 1.61 | 2.37 | 1.88 |
| Mapre1 | CD | microtubule-associated protein, RP/EB family, member 1 | 1.75 | 2.06 | 1.76 |
| Ndufb6 | CD | NADH dehydrogenase (ubiquinone) 1 beta subcomplex, 6, 17kDa | 2.36 | 2.85 | 1.60 |
| Pctk3 | CD | PCTAIRE protein kinase 3 | 2.15 | 3.61 | 2.63 |
| Plk3 | CD | polo-like kinase 3 (Drosophila) | 1.25 | 2.28 | 1.73 |
| Syk | CD | spleen tyrosine kinase | 1.97 | 2.27 | 1.37 |
| Tmem85 | CD | transmembrane protein 85 | 1.89 | 4.41 | 2.78 |
| Txn1 | CD | thioredoxin | 2.45 | 3.04 | 1.73 |
| Col5a3 | ECM | collagen, type V, alpha 3 | 4.41 | 8.40 | 5.25 |
| Lgals3 | ECM | lectin, galactoside-binding, soluble, 3 | 3.41 | 6.11 | 3.93 |
| Sdc1 | ECM | syndecan 1 | 3.54 | 5.79 | 3.19 |
| Adam8 | ECM2 | ADAM metallopeptidase domain 8 | 1.95 | 2.45 | 1.35 |
| Adamts12 | ECM2 | ADAM metallopeptidase with thrombospondin type 1 motif, 12 | 3.14 | 4.24 | 3.64 |
| Adamts4 | ECM2 | ADAM metallopeptidase with thrombospondin type 1 motif, 4 | 2.37 | 6.21 | 4.31 |
| Adamts7 | ECM2 | ADAM metallopeptidase with thrombospondin type 1 motif, 7 | 2.46 | 3.22 | 2.86 |
| Amz1 | ECM2 | archaelysin family metallopeptidase 1 | 2.34 | 3.18 | 2.40 |
| Cd44 | ECM2 | CD44 molecule (Indian blood group) | 4.13 | 6.67 | 5.60 |
| Cndp2 | ECM2 | CNDP dipeptidase 2 (metallopeptidase M20 family) | 2.11 | 2.74 | 1.89 |
| Ermp1 | ECM2 | endoplasmic reticulum metallopeptidase 1 | 2.47 | 2.88 | 1.82 |
| Hyal1 | ECM2 | hyaluronoglucosaminidase 1 | 2.24 | 3.66 | 2.37 |
| Hyal3 | ECM2 | hyaluronoglucosaminidase 3 | 1.38 | 2.23 | 1.55 |
| Mmp12 | ECM2 | matrix metallopeptidase 12 (macrophage elastase) | 13.59 | 33.42 | 18.16 |
| Mmp19 | ECM2 | matrix metallopeptidase 19 | 5.03 | 12.86 | 8.00 |
| Mmp9 | ECM2 | matrix metallopeptidase 9 (gelatinase B, 92kDa gelatinase, 92kDa type IV collagenase) | 1.42 | 3.16 | 2.50 |
| Pcsk1 | ECM2 | proprotein convertase subtilisin/kexin type 1 | 4.57 | 8.05 | 5.33 |
| Serpinb8 | ECM2 | serpin peptidase inhibitor, clade B (ovalbumin), member 8 | 2.34 | 3.38 | 2.33 |
| Serpine2 | ECM2 | serpin peptidase inhibitor, clade E (nexin, plasminogen activator inhibitor type 1), member 2 | 1.52 | 2.01 | 1.55 |
| Timp1 | ECM2 | TIMP metallopeptidase inhibitor 1 | 3.57 | 4.53 | 3.04 |
| Csf1r | GF | colony stimulating factor 1 receptor | 2.94 | 4.09 | 2.94 |
| Inhba | GF | inhibin, beta A | 4.43 | 7.00 | 4.56 |
| Jag1 | GF | jagged 1 (Alagille syndrome) | 4.51 | 6.82 | 4.89 |
| Pdgfb | GF | platelet-derived growth factor beta polypeptide (simian sarcoma viral (v-sis) oncogene homolog) | 2.15 | 4.11 | 2.92 |
| Tgfbr1 | GF | transforming growth factor, beta receptor 1 | 2.44 | 3.48 | 2.56 |
| Igfbp4 | GF2 | insulin-like growth factor binding protein 4 | 4.15 | 5.34 | 4.23 |
| Notch1 | GF2 | Notch homolog 1, translocation-associated (Drosophila) | 1.87 | 2.17 | 1.76 |
| Sfrp4 | GF2 | secreted frizzled-related protein 4 | 5.50 | 8.96 | 6.26 |
| Tgfb1i1 | GF2 | transforming growth factor beta 1 induced transcript 1 | 1.77 | 2.33 | 2.03 |
| Wnt5a | GF2 | wingless-type MMTV integration site family, member 5A | 3.65 | 8.15 | 6.47 |
| Wnt7b | GF2 | wingless-type MMTV integration site family, member 7B | 1.76 | 2.77 | 1.38 |
| Ccl2 | Inf | chemokine (C-C motif) ligand 2 | 3.09 | 4.89 | 3.21 |
| Ccl7 | Inf | chemokine (C-C motif) ligand 7 | 4.08 | 6.88 | 2.30 |
| Csf1 | Inf | colony stimulating factor 1 (macrophage) | 2.51 | 2.79 | 2.27 |
| Ifngr2 | Inf | interferon gamma receptor 2 (interferon gamma transducer 1) | 1.82 | 2.25 | 1.72 |
| Il11 | Inf | interleukin 11 | 1.52 | 2.06 | 1.21 |
| Il18 | Inf | interleukin 18 (interferon-gamma-inducing factor) | 3.63 | 4.23 | 3.69 |
| Il1rn | Inf | interleukin 1 receptor antagonist | 2.43 | 3.46 | 1.98 |
| Il7 | Inf | interleukin 7 | 1.90 | 2.35 | 1.99 |
| Lif | Inf | leukemia inhibitory factor (cholinergic differentiation factor) | 3.06 | 5.87 | 2.84 |
| Pf4 | Inf | platelet factor 4 | 2.32 | 2.80 | 1.64 |
| Tnfrsf11a | Inf | tumor necrosis factor receptor superfamily, member 11a, NFKB activator | 3.01 | 3.73 | 2.71 |
| Tnfrsf12a | Inf | tumor necrosis factor receptor superfamily, member 12A | 1.89 | 2.21 | 1.90 |
| Tnfrsf1b | Inf | tumor necrosis factor receptor superfamily, member 1B | 2.80 | 3.30 | 2.37 |
| Tnfsf11 | Inf | tumor necrosis factor (ligand) superfamily, member 11 | 1.98 | 9.51 | 6.04 |
| Tnfsf13 | Inf | tumor necrosis factor (ligand) superfamily, member 13 | 2.25 | 3.52 | 2.63 |
| Ccl9 | Inf | chemokine (C-C motif) ligand 9 | 4.65 | 14.74 | 5.41 |
| Ccr1 | Inf | chemokine (C-C motif) receptor 1 | 4.53 | 10.73 | 5.43 |
| Ccr5 | Inf | chemokine (C-C motif) receptor 5 | 3.27 | 3.58 | 2.60 |
| Cd97 | Inf | CD97 molecule | 2.31 | 4.40 | 3.43 |
| Clec2d | Inf | C-type lectin domain family 2, member D | 3.63 | 4.87 | 4.11 |
| Cx3cr1 | Inf | chemokine (C-X3-C motif) receptor 1 | 2.85 | 3.84 | 2.92 |
| Il2rg | Inf | interleukin 2 receptor, gamma (severe combined immunodeficiency) | 1.83 | 2.39 | 2.01 |
| Lrp10 | Inf | low density lipoprotein receptor-related protein 10 | 1.27 | 2.16 | 1.59 |
| Alcam | Inf2 | activated leukocyte cell adhesion molecule | 4.22 | 5.29 | 4.73 |
| Arhgap22 | Inf2 | Rho GTPase activating protein 22 | 3.23 | 4.69 | 3.22 |
| Arhgap27 | Inf2 | Rho GTPase activating protein 27 | 2.04 | 2.31 | 1.78 |
| Cd244 | Inf2 | CD244 molecule, natural killer cell receptor 2B4 | 2.54 | 2.81 | 1.88 |
| Cd63 | Inf2 | CD63 molecule | 1.54 | 2.36 | 1.64 |
| Cd68 | Inf2 | CD68 molecule | 3.21 | 4.95 | 3.35 |
| Cd82 | Inf2 | CD82 molecule | 1.68 | 2.33 | 1.69 |
| Cd84 | Inf2 | CD84 molecule | 4.01 | 6.82 | 5.57 |
| Commd6 | Inf2 | COMM domain containing 6 | 1.43 | 2.07 | 1.50 |
| Cxcl16 | Inf2 | chemokine (C-X-C motif) ligand 16 | 3.15 | 3.70 | 3.01 |
| Dgki | Inf2 | diacylglycerol kinase, iota | 4.07 | 6.23 | 3.74 |
| Dusp2 | Inf2 | dual specificity phosphatase 2 | 1.38 | 2.07 | 1.20 |
| Dusp4 | Inf2 | dual specificity phosphatase 4 | 3.43 | 6.99 | 3.27 |
| F2r | Inf2 | coagulation factor II (thrombin) receptor | 3.23 | 5.10 | 3.69 |
| F2rl1 | Inf2 | coagulation factor II (thrombin) receptor-like 1 | 1.98 | 2.22 | 1.38 |
| Fcgr2a | Inf2 | Fc fragment of IgG, low affinity IIb, receptor (CD32) | 2.99 | 3.27 | 2.81 |
| Fkbp1a | Inf2 | FK506 binding protein 1A, 12kDa | 1.88 | 2.40 | 2.12 |
| Fosl1 | Inf2 | FOS-like antigen 1 | 1.51 | 2.11 | 1.58 |
| Gab2 | Inf2 | GRB2-associated binding protein 2 | 1.84 | 2.35 | 2.00 |
| Glipr1 | Inf2 | GLI pathogenesis-related 1 | 4.57 | 4.98 | 2.80 |
| Gpr183 | Inf2 | G protein-coupled receptor 183 | 3.70 | 4.27 | 2.89 |
| Gpr68 | Inf2 | G protein-coupled receptor 68 | 2.85 | 5.23 | 3.56 |
| H2-T24 | Inf2 | histocompatibility 2, T region locus 24 | 1.54 | 2.06 | 1.38 |
| Havcr2 | Inf2 | hepatitis A virus cellular receptor 2 | 1.64 | 2.09 | 1.67 |
| Hcls1 | Inf2 | hematopoietic cell-specific Lyn substrate 1 | 2.25 | 2.76 | 1.73 |
| Hsp90aa1 | Inf2 | heat shock protein 90kDa alpha (cytosolic), class A member 1 | 1.93 | 2.07 | 1.77 |
| Ifi27l | Inf2 | interferon, alpha-inducible protein 27 | 2.15 | 3.02 | 1.38 |
| Ifi30 | Inf2 | interferon, gamma-inducible protein 30 | 2.57 | 2.81 | 2.02 |
| Itga2 | Inf2 | integrin, alpha 2 (CD49B, alpha 2 subunit of VLA-2 receptor) | 1.74 | 3.19 | 2.41 |
| Itgam | Inf2 | integrin, alpha M (complement component 3 receptor 3 subunit) | 5.47 | 5.92 | 3.82 |
| Itgax | Inf2 | integrin, alpha X (complement component 3 receptor 4 subunit) | 3.45 | 5.68 | 3.22 |
| Jdp2 | Inf2 | Jun dimerization protein 2 | 3.78 | 6.96 | 4.59 |
| Laptm5 | Inf2 | lysosomal protein transmembrane 5 | 1.94 | 2.73 | 1.84 |
| Lat | Inf2 | linker for activation of T cells | 1.88 | 3.60 | 1.98 |
| Lat2 | Inf2 | linker for activation of T cells family, member 2 | 2.79 | 3.29 | 2.27 |
| Lcp2 | Inf2 | lymphocyte cytosolic protein 2 (SH2 domain containing leukocyte protein of 76kDa) | 2.64 | 3.64 | 2.85 |
| Lgals3bp | Inf2 | lectin, galactoside-binding, soluble, 3 binding protein | 2.55 | 4.04 | 2.40 |
| Lpcat1 | Inf2 | lysophosphatidylcholine acyltransferase 1 | 1.90 | 3.32 | 1.88 |
| Lrp12 | Inf2 | low density lipoprotein-related protein 12 | 1.59 | 2.55 | 1.63 |
| Ly6e | Inf2 | lymphocyte antigen 6 complex, locus E | 1.26 | 2.11 | 1.32 |
| Ly96 | Inf2 | lymphocyte antigen 96 | 2.77 | 3.28 | 2.63 |
| Map2k3 | Inf2 | mitogen-activated protein kinase kinase 3 | 1.97 | 2.64 | 1.94 |
| Matk | Inf2 | megakaryocyte-associated tyrosine kinase | 2.32 | 2.68 | 1.77 |
| Ndfip2 | Inf2 | Nedd4 family interacting protein 2 | 1.47 | 2.17 | 1.63 |
| Nfkb2 | Inf2 | nuclear factor of kappa light polypeptide gene enhancer in B-cells 2 (p49/p100) | 1.32 | 2.40 | 1.60 |
| Nfkbie | Inf2 | nuclear factor of kappa light polypeptide gene enhancer in B-cells inhibitor, epsilon | 1.83 | 2.64 | 1.87 |
| Nuak2 | Inf2 | NUAK family, SNF1-like kinase, 2 | 2.60 | 3.55 | 2.32 |
| Oscar | Inf2 | osteoclast associated, immunoglobulin-like receptor | 2.79 | 3.67 | 2.03 |
| Pak6 | Inf2 | p21 protein (Cdc42/Rac)-activated kinase 6 | 1.72 | 2.47 | 1.52 |
| Pik3cb | Inf2 | phosphoinositide-3-kinase, catalytic, beta polypeptide | 4.36 | 7.04 | 5.28 |
| Pkn3 | Inf2 | protein kinase N3 | 1.48 | 2.52 | 1.65 |
| Pla2g2d | Inf2 | phospholipase A2, group IID | 1.40 | 2.49 | 2.05 |
| Pla2g7 | Inf2 | phospholipase A2, group VII (platelet-activating factor acetylhydrolase, plasma) | 3.72 | 6.87 | 3.87 |
| Plaur | Inf2 | plasminogen activator, urokinase receptor | 2.20 | 2.71 | 2.30 |
| Ppargc1b | Inf2 | peroxisome proliferator-activated receptor gamma, coactivator 1 beta | 2.34 | 3.51 | 2.43 |
| Ppp1r14b | Inf2 | protein phosphatase 1, regulatory (inhibitor) subunit 14B | 2.25 | 2.82 | 1.83 |
| Ppp2r5b | Inf2 | protein phosphatase 2, regulatory subunit B', beta isoform | 1.58 | 2.12 | 1.73 |
| Prdm1 | Inf2 | PR domain containing 1, with ZNF domain | 2.13 | 4.56 | 2.80 |
| Prkch | Inf2 | protein kinase C, eta | 2.55 | 3.25 | 2.48 |
| Psma3 | Inf2 | proteasome (prosome, macropain) subunit, alpha type, 3 | 1.62 | 2.02 | 1.56 |
| Ptgir | Inf2 | prostaglandin I2 (prostacyclin) receptor (IP) | 1.94 | 2.35 | 1.93 |
| Ptpn22 | Inf2 | protein tyrosine phosphatase, non-receptor type 22 (lymphoid) | 2.51 | 4.07 | 2.92 |
| Ptpre | Inf2 | protein tyrosine phosphatase, receptor type, E | 2.39 | 5.58 | 2.80 |
| Ptpro | Inf2 | protein tyrosine phosphatase, receptor type, O | 2.40 | 2.74 | 2.47 |
| Rab11fip1 | Inf2 | RAB11 family interacting protein 1 (class I) | 1.55 | 2.55 | 1.60 |
| Rabgef1 | Inf2 | RAB guanine nucleotide exchange factor (GEF) 1 | 1.52 | 2.31 | 1.69 |
| Rap1gds1 | Inf2 | RAP1, GTP-GDP dissociation stimulator 1 | 1.96 | 2.51 | 2.14 |
| Rasgrp1 | Inf2 | RAS guanyl releasing protein 1 (calcium and DAG-regulated) | 2.12 | 3.41 | 2.36 |
| Rin1 | Inf2 | Ras and Rab interactor 1 | 1.63 | 2.60 | 2.01 |
| Rin3 | Inf2 | Ras and Rab interactor 3 | 1.93 | 2.40 | 1.50 |
| Ripk2 | Inf2 | receptor-interacting serine-threonine kinase 2 | 2.28 | 2.77 | 2.05 |
| Ripk3 | Inf2 | receptor-interacting serine-threonine kinase 3 | 3.32 | 3.57 | 2.51 |
| Sbno2 | Inf2 | strawberry notch homolog 2 (Drosophila) | 1.61 | 2.20 | 1.93 |
| Scarf1 | Inf2 | scavenger receptor class F, member 1 | 1.71 | 2.77 | 2.15 |
| Selplg | Inf2 | selectin P ligand | 2.14 | 2.43 | 1.50 |
| Sema4a | Inf2 | sema domain, immunoglobulin domain (Ig), transmembrane domain (TM) and short cytoplasmic domain, (semaphorin) 4A | 2.11 | 2.36 | 1.24 |
| Sema4d | Inf2 | sema domain, immunoglobulin domain (Ig), transmembrane domain (TM) and short cytoplasmic domain, (semaphorin) 4D | 2.63 | 4.87 | 3.15 |
| Sfpi1 | Inf2 | spleen focus forming virus (SFFV) proviral integration oncogene spi1 | 3.34 | 5.20 | 3.32 |
| Sirpa | Inf2 | signal-regulatory protein alpha | 1.92 | 2.51 | 2.07 |
| Slamf6 | Inf2 | SLAM family member 6 | 1.71 | 9.14 | 3.62 |
| Src | Inf2 | v-src sarcoma (Schmidt-Ruppin A-2) viral oncogene homolog (avian) | 3.01 | 4.99 | 2.96 |
| Tank | Inf2 | TRAF family member-associated NFKB activator | 2.50 | 3.79 | 3.02 |
| Tbxas1 | Inf2 | thromboxane A synthase 1 (platelet) | 2.88 | 4.33 | 3.41 |
| Tcirg1 | Inf2 | T-cell, immune regulator 1, ATPase, H+ transporting, lysosomal V0 subunit A3 | 3.03 | 4.83 | 2.79 |
| Tfpi2 | Inf2 | tissue factor pathway inhibitor 2 | 5.68 | 8.02 | 3.27 |
| Tm7sf4 | Inf2 | transmembrane 7 superfamily member 4 | 3.16 | 9.31 | 5.44 |
| Trem1 | Inf2 | triggering receptor expressed on myeloid cells 1 | 2.33 | 2.52 | 2.16 |
| Tyrobp | Inf2 | TYRO protein tyrosine kinase binding protein | 2.85 | 3.48 | 2.38 |
| Vash2 | Inf2 | vasohibin 2 | 2.51 | 3.80 | 2.55 |
| Vasp | Inf2 | vasodilator-stimulated phosphoprotein | 1.57 | 2.00 | 1.39 |
| Vwf | Inf2 | von Willebrand factor | 2.09 | 2.30 | 1.96 |
| Ywhag | Inf2 | tyrosine 3-monooxygenase/tryptophan 5-monooxygenase activation protein, gamma polypeptide | 1.68 | 2.43 | 1.97 |
| Ywhah | Inf2 | tyrosine 3-monooxygenase/tryptophan 5-monooxygenase activation protein, eta polypeptide | 1.95 | 2.13 | 1.77 |
| Abl2 | Meta | v-abl Abelson murine leukemia viral oncogene homolog 2 (arg, Abelson-related gene) | 1.60 | 2.41 | 1.98 |
| Aco2 | Meta | aconitase 2, mitochondrial | 1.79 | 2.15 | 1.76 |
| Acsl5 | Meta | acyl-CoA synthetase long-chain family member 5 | 2.00 | 2.19 | 1.72 |
| Actg1 | Meta | actin, gamma 1 | 1.96 | 2.17 | 1.76 |
| Ada | Meta | adenosine deaminase | 2.78 | 3.81 | 2.71 |
| Adrbk2 | Meta | adrenergic, beta, receptor kinase 2 | 3.14 | 5.39 | 4.03 |
| Ak2 | Meta | adenylate kinase 2 | 1.85 | 2.31 | 1.42 |
| Angptl4 | Meta | angiopoietin-like 4 | 1.88 | 2.02 | 1.67 |
| Ankdd1a | Meta | ankyrin repeat and death domain containing 1A | 1.76 | 2.17 | 1.96 |
| Anpep | Meta | alanyl (membrane) aminopeptidase | 2.02 | 3.28 | 2.63 |
| Arhgdia | Meta | Rho GDP dissociation inhibitor (GDI) alpha | 1.53 | 2.07 | 1.67 |
| Arpc4 | Meta | actin related protein 2/3 complex, subunit 4, 20kDa | 1.83 | 2.54 | 2.05 |
| Atic | Meta | 5-aminoimidazole-4-carboxamide ribonucleotide formyltransferase/IMP cyclohydrolase | 1.68 | 2.01 | 1.51 |
| Baiap2 | Meta | BAI1-associated protein 2 | 1.60 | 2.44 | 2.00 |
| Capg | Meta | capping protein (actin filament), gelsolin-like | 2.83 | 5.20 | 3.92 |
| Car2 | Meta | carbonic anhydrase II | 3.69 | 3.95 | 2.31 |
| Cass4 | Meta | Cas scaffolding protein family member 4 | 2.06 | 3.77 | 2.23 |
| Chchd4 | Meta | coiled-coil-helix-coiled-coil-helix domain containing 4 | 1.91 | 2.01 | 1.39 |
| Ckb | Meta | creatine kinase, brain | 3.15 | 5.71 | 3.24 |
| Cnih2 | Meta | cornichon homolog 2 (Drosophila) | 1.96 | 3.83 | 2.46 |
| Cox4i1 | Meta | cytochrome c oxidase subunit IV isoform 1 | 1.72 | 2.12 | 1.41 |
| Cox5a | Meta | cytochrome c oxidase subunit Va | 2.05 | 2.62 | 1.54 |
| Cox7b | Meta | cytochrome c oxidase subunit VIIb | 1.88 | 2.25 | 1.44 |
| Crat | Meta | carnitine acetyltransferase | 1.69 | 2.14 | 1.75 |
| Csrp1 | Meta | cysteine and glycine-rich protein 1 | 1.60 | 2.14 | 1.67 |
| Cstb | Meta | cystatin B (stefin B) | 2.55 | 3.25 | 2.36 |
| Cycs | Meta | cytochrome c, somatic | 2.29 | 2.95 | 2.13 |
| Cyp2s1 | Meta | cytochrome P450, family 2, subfamily S, polypeptide 1 | 1.74 | 2.59 | 1.51 |
| Dhrs9 | Meta | dehydrogenase/reductase (SDR family) member 9 | 4.12 | 11.17 | 6.36 |
| Dlat | Meta | dihydrolipoamide S-acetyltransferase | 1.83 | 2.05 | 1.65 |
| Dnase1l1 | Meta | deoxyribonuclease I-like 1 | 1.55 | 2.11 | 1.81 |
| Dnase2b | Meta | deoxyribonuclease II beta | 1.70 | 4.80 | 3.32 |
| Doc2a | Meta | double C2-like domains, alpha | 1.40 | 5.42 | 1.74 |
| Eef1e1 | Meta | eukaryotic translation elongation factor 1 epsilon 1 | 2.17 | 2.76 | 1.77 |
| Eif2b2 | Meta | eukaryotic translation initiation factor 2B, subunit 2 beta, 39kDa | 2.22 | 2.48 | 1.93 |
| Ext1 | Meta | exostoses (multiple) 1 | 2.57 | 3.23 | 2.66 |
| Fads1 | Meta | fatty acid desaturase 1 | 2.20 | 2.61 | 2.14 |
| Fads3 | Meta | fatty acid desaturase 3 | 2.72 | 3.38 | 2.90 |
| Fbxl7 | Meta | F-box and leucine-rich repeat protein 7 | 1.88 | 2.69 | 2.17 |
| Fermt3 | Meta | fermitin family homolog 3 (Drosophila) | 3.09 | 3.44 | 2.34 |
| Flna | Meta | filamin A, alpha | 2.58 | 3.08 | 2.80 |
| Frrs1 | Meta | ferric-chelate reductase 1 | 3.37 | 4.69 | 4.07 |
| G6pd | Meta | glucose-6-phosphate dehydrogenase | 1.84 | 2.20 | 1.62 |
| Galns | Meta | galactosamine (N-acetyl)-6-sulfate sulfatase | 2.78 | 3.21 | 2.84 |
| Gfod1 | Meta | glucose-fructose oxidoreductase domain containing 1 | 1.99 | 4.11 | 2.51 |
| Gla | Meta | galactosidase, alpha | 2.67 | 4.37 | 3.52 |
| Glrx1 | Meta | glutaredoxin (thioltransferase) | 2.02 | 3.04 | 2.24 |
| Gng11 | Meta | guanine nucleotide binding protein (G protein), gamma 11 | 2.16 | 2.90 | 2.19 |
| Gngt2 | Meta | guanine nucleotide binding protein (G protein), gamma transducing activity polypeptide 2 | 2.21 | 3.32 | 2.47 |
| Gnptab | Meta | N-acetylglucosamine-1-phosphate transferase, alpha and beta subunits | 2.51 | 4.25 | 2.68 |
| Got1 | Meta | glutamic-oxaloacetic transaminase 1, soluble (aspartate aminotransferase 1) | 1.80 | 3.02 | 1.99 |
| Gpnmb | Meta | glycoprotein (transmembrane) nmb | 1.95 | 2.63 | 2.13 |
| Gpr39 | Meta | G protein-coupled receptor 39 | 1.95 | 2.86 | 1.40 |
| Gusb | Meta | glucuronidase, beta | 1.99 | 2.77 | 1.80 |
| Hagh | Meta | hydroxyacylglutathione hydrolase | 1.64 | 2.36 | 1.58 |
| Hk3 | Meta | hexokinase 3 (white cell) | 1.79 | 2.16 | 1.40 |
| Hmox1 | Meta | heme oxygenase (decycling) 1 | 8.02 | 12.68 | 6.48 |
| Jtv1 | Meta | aminoacyl tRNA synthetase complex-interacting multifunctional protein 2 | 1.71 | 2.14 | 1.49 |
| Ldha | Meta | lactate dehydrogenase A | 1.30 | 2.06 | 1.45 |
| Limk1 | Meta | LIM domain kinase 1 | 2.13 | 2.56 | 1.95 |
| Llgl1 | Meta | lethal giant larvae homolog 1 (Drosophila) | 1.30 | 2.02 | 1.55 |
| Manf | Meta | mesencephalic astrocyte-derived neurotrophic factor | 1.89 | 2.28 | 1.68 |
| Mdh2 | Meta | malate dehydrogenase 2, NAD (mitochondrial) | 1.84 | 2.04 | 1.60 |
| Mfng | Meta | MFNG O-fucosylpeptide 3-beta-N-acetylglucosaminyltransferase | 2.35 | 4.45 | 2.50 |
| Mical1 | Meta | microtubule associated monoxygenase, calponin and LIM domain containing 1 | 1.64 | 2.32 | 1.99 |
| Mobkl2a | Meta | MOB1, Mps One Binder kinase activator-like 2A (yeast) | 1.55 | 2.03 | 1.61 |
| Mt3 | Meta | metallothionein 3 | 2.79 | 8.95 | 5.22 |
| Mtfr1 | Meta | mitochondrial fission regulator 1 | 1.68 | 2.12 | 1.38 |
| Mvp | Meta | major vault protein | 2.11 | 3.32 | 2.57 |
| Ncald | Meta | neurocalcin delta | 2.07 | 2.47 | 2.18 |
| Ndufv1 | Meta | NADH dehydrogenase (ubiquinone) flavoprotein 1, 51kDa | 1.63 | 2.07 | 1.47 |
| Neu1 | Meta | sialidase 1 (lysosomal sialidase) | 1.50 | 2.19 | 1.53 |
| Npc2 | Meta | Niemann-Pick disease, type C2 | 2.37 | 3.26 | 2.63 |
| Nsg1 | Meta | neuron specific gene family member | 3.87 | 5.44 | 3.82 |
| Nxn | Meta | nucleoredoxin | 1.90 | 2.67 | 2.27 |
| Oxr1 | Meta | oxidation resistance 1 | 2.38 | 3.13 | 2.82 |
| P2ry2 | Meta | purinergic receptor P2Y, G-protein coupled, 2 | 1.51 | 2.26 | 1.78 |
| Parvb | Meta | parvin, beta | 2.40 | 3.34 | 2.40 |
| Pdlim7 | Meta | PDZ and LIM domain 7 (enigma) | 1.58 | 2.37 | 1.65 |
| Pfn1 | Meta | profilin 1 | 2.35 | 3.82 | 2.08 |
| Pgs1 | Meta | phosphatidylglycerophosphate synthase 1 | 1.80 | 2.35 | 1.78 |
| Phyhd1 | Meta | phytanoyl-CoA dioxygenase domain containing 1 | 1.66 | 2.26 | 1.70 |
| Pik3ap1 | Meta | phosphoinositide-3-kinase adaptor protein 1 | 1.88 | 2.36 | 1.54 |
| Pip5k1b | Meta | phosphatidylinositol-4-phosphate 5-kinase, type I, beta | 2.56 | 3.41 | 2.21 |
| Pla2g15 | Meta | phospholipase A2, group XV | 1.80 | 2.08 | 1.60 |
| Plek | Meta | pleckstrin | 3.16 | 3.61 | 2.35 |
| Plscr2 | Meta | phospholipid scramblase 2 | 1.71 | 4.02 | 3.15 |
| Pop4 | Meta | processing of precursor 4, ribonuclease P/MRP subunit (S. cerevisiae) | 2.47 | 2.64 | 1.90 |
| Prdx5 | Meta | peroxiredoxin 5 | 2.99 | 4.30 | 2.29 |
| Psd4 | Meta | pleckstrin and Sec7 domain containing 4 | 2.26 | 3.21 | 2.10 |
| Psen2 | Meta | presenilin 2 (Alzheimer disease 4) | 1.45 | 2.53 | 1.78 |
| Psmd14 | Meta | proteasome (prosome, macropain) 26S subunit, non-ATPase, 14 | 1.88 | 2.08 | 1.24 |
| Pstpip1 | Meta | proline-serine-threonine phosphatase interacting protein 1 | 2.96 | 3.95 | 2.78 |
| Pter | Meta | phosphotriesterase related | 2.00 | 2.57 | 1.85 |
| Ptk2b | Meta | PTK2B protein tyrosine kinase 2 beta | 3.14 | 3.58 | 2.20 |
| Rab1b | Meta | RAB1B, member RAS oncogene family | 1.50 | 2.17 | 1.22 |
| Rab38 | Meta | RAB38, member RAS oncogene family | 3.12 | 4.12 | 3.00 |
| Rab4a | Meta | RAB4A, member RAS oncogene family | 1.42 | 2.00 | 1.72 |
| Rac2 | Meta | ras-related C3 botulinum toxin substrate 2 (rho family, small GTP binding protein Rac2) | 2.93 | 3.74 | 1.85 |
| Ralgds | Meta | ral guanine nucleotide dissociation stimulator | 1.71 | 3.78 | 2.97 |
| Rassf5 | Meta | Ras association (RalGDS/AF-6) domain family member 5 | 1.85 | 2.20 | 1.53 |
| Rcan1 | Meta | regulator of calcineurin 1 | 1.71 | 2.38 | 1.67 |
| Rnf149 | Meta | ring finger protein 149 | 2.15 | 2.83 | 1.94 |
| Rnh1 | Meta | ribonuclease/angiogenin inhibitor 1 | 1.41 | 2.03 | 1.77 |
| S100a4 | Meta | S100 calcium binding protein A4 | 2.62 | 4.56 | 3.81 |
| Sat1 | Meta | spermidine/spermine N1-acetyltransferase 1 | 1.63 | 2.43 | 1.92 |
| Sdhc | Meta | succinate dehydrogenase complex, subunit C, integral membrane protein, 15kDa | 1.86 | 2.16 | 1.44 |
| Sdhd | Meta | succinate dehydrogenase complex, subunit D, integral membrane protein | 1.57 | 2.02 | 1.58 |
| Sgpl1 | Meta | sphingosine-1-phosphate lyase 1 | 1.77 | 2.28 | 2.03 |
| Sh3bp2 | Meta | SH3-domain binding protein 2 | 2.56 | 3.42 | 2.04 |
| Siglec15 | Meta | sialic acid binding Ig-like lectin 15 | 2.07 | 2.70 | 1.64 |
| Snrpd2 | Meta | small nuclear ribonucleoprotein D2 polypeptide 16.5kDa | 2.05 | 2.23 | 1.21 |
| Sphk1 | Meta | sphingosine kinase 1 | 1.80 | 2.50 | 2.21 |
| Sptlc2 | Meta | serine palmitoyltransferase, long chain base subunit 2 | 1.57 | 2.27 | 1.97 |
| St8sia6 | Meta | ST8 alpha-N-acetyl-neuraminide alpha-2,8-sialyltransferase 6 | 2.69 | 5.10 | 3.05 |
| Stard3nl | Meta | STARD3 N-terminal like | 1.92 | 2.33 | 1.38 |
| Tgm2 | Meta | transglutaminase 2 (C polypeptide, protein-glutamine-gamma-glutamyltransferase) | 2.21 | 4.16 | 2.97 |
| Tiam1 | Meta | T-cell lymphoma invasion and metastasis 1 | 2.42 | 3.23 | 2.06 |
| Tuba1b | Meta | tubulin, alpha 1b | 2.10 | 2.20 | 1.22 |
| Tuba1c | Meta | tubulin, alpha 1c | 2.13 | 2.52 | 1.35 |
| Tubb6 | Meta | tubulin, beta 6 | 3.14 | 3.96 | 3.33 |
| Txnrd1 | Meta | thioredoxin reductase 1 | 1.82 | 2.36 | 1.88 |
| Ubash3b | Meta | ubiquitin associated and SH3 domain containing, B | 2.23 | 2.52 | 1.87 |
| Ube2g2 | Meta | ubiquitin-conjugating enzyme E2G 2 (UBC7 homolog, yeast) | 1.60 | 2.14 | 1.73 |
| Upp1 | Meta | uridine phosphorylase 1 | 3.52 | 4.61 | 3.06 |
| Uqcrh | Meta | ubiquinol-cytochrome c reductase hinge protein-like | 1.97 | 2.08 | 1.02 |
| Vcl | Meta | vinculin | 1.89 | 2.95 | 2.48 |
| Abi3 | Other | ABI family, member 3 | 2.21 | 2.69 | 1.78 |
| Abr | Other | active BCR-related gene | 1.33 | 2.21 | 1.79 |
| Acot7 | Other | acyl-CoA thioesterase 7 | 1.51 | 2.00 | 1.71 |
| Akap6 | Other | A kinase (PRKA) anchor protein 6 | 2.01 | 3.64 | 2.63 |
| Apbb1ip | Other | amyloid beta (A4) precursor protein-binding, family B, member 1 interacting protein | 2.18 | 3.05 | 2.32 |
| Arap3 | Other | ArfGAP with RhoGAP domain, ankyrin repeat and PH domain 3 | 2.37 | 3.19 | 2.11 |
| Arrb1 | Other | arrestin, beta 1 | 1.87 | 2.02 | 1.74 |
| Avpi1 | Other | arginine vasopressin-induced 1 | 2.31 | 5.03 | 2.84 |
| Bcar3 | Other | breast cancer anti-estrogen resistance 3 | 2.10 | 2.20 | 1.53 |
| Bst2 | Other | bone marrow stromal cell antigen 2 | 1.86 | 2.46 | 1.50 |
| Cblb | Other | Cas-Br-M (murine) ecotropic retroviral transforming sequence b | 2.09 | 3.47 | 2.61 |
| Crip | Other | cysteine-rich protein 1 (intestinal) | 4.00 | 8.71 | 6.63 |
| Cttnbp2nl | Other | CTTNBP2 N-terminal like | 2.03 | 2.37 | 2.12 |
| Dok3 | Other | docking protein 3 | 2.14 | 2.58 | 1.91 |
| Dot1l | Other | DOT1-like, histone H3 methyltransferase (S. cerevisiae) | 1.39 | 2.20 | 1.27 |
| Dsg2 | Other | desmoglein 2 | 1.84 | 4.29 | 2.42 |
| Dsg3 | Other | desmoglein 3 (pemphigus vulgaris antigen) | 1.18 | 3.40 | 1.34 |
| Efhd2 | Other | EF-hand domain family, member D2 | 1.61 | 2.22 | 1.50 |
| Emp3 | Other | epithelial membrane protein 3 | 2.12 | 2.99 | 2.46 |
| Errfi1 | Other | ERBB receptor feedback inhibitor 1 | 1.36 | 3.01 | 2.49 |
| Fam43a | Other | family with sequence similarity 43, member A | 1.32 | 2.29 | 1.60 |
| Fgd3 | Other | FYVE, RhoGEF and PH domain containing 3 | 2.19 | 2.61 | 2.15 |
| Fhl3 | Other | four and a half LIM domains 3 | 1.52 | 2.02 | 1.52 |
| Fmnl1 | Other | formin-like 1 | 2.43 | 2.62 | 2.12 |
| Frmd4a | Other | FERM domain containing 4A | 1.95 | 2.45 | 2.18 |
| Git1 | Other | G protein-coupled receptor kinase interacting ArfGAP 1 | 1.64 | 2.17 | 1.67 |
| Gpr137b | Other | G protein-coupled receptor 137B | 3.42 | 5.39 | 3.95 |
| Lmna | Other | lamin A/C | 2.57 | 4.65 | 3.93 |
| Lrrc15 | Other | leucine rich repeat containing 15 | 5.52 | 10.07 | 8.86 |
| Lrrc8b | Other | leucine rich repeat containing 8 family, member B | 1.88 | 2.58 | 1.98 |
| Mbp | Other | myelin basic protein | 2.03 | 2.19 | 1.81 |
| Mesdc1 | Other | mesoderm development candidate 1 | 1.54 | 2.09 | 1.35 |
| Mkl1 | Other | megakaryoblastic leukemia (translocation) 1 | 2.28 | 2.80 | 2.14 |
| Ms4a7 | Other | membrane-spanning 4-domains, subfamily A, member 7 | 2.82 | 2.99 | 1.85 |
| Myo5a | Other | myosin VA (heavy chain 12, myoxin) | 2.95 | 3.78 | 3.42 |
| Nrp2 | Other | neuropilin 2 | 1.63 | 2.12 | 1.83 |
| Odz4 | Other | odz, odd Oz/ten-m homolog 4 (Drosophila) | 2.14 | 2.99 | 2.31 |
| Pion | Other | pigeon homolog (Drosophila) | 2.89 | 4.19 | 3.07 |
| Plekhb2 | Other | pleckstrin homology domain containing, family B (evectins) member 2 | 2.72 | 3.94 | 3.61 |
| Plekhm1 | Other | pleckstrin homology domain containing, family M (with RUN domain) member 1 | 1.94 | 2.92 | 1.79 |
| Plekhm3 | Other | pleckstrin homology domain containing, family M, member 3 | 1.38 | 2.61 | 1.56 |
| Plekho1 | Other | pleckstrin homology domain containing, family O member 1 | 2.56 | 3.45 | 2.29 |
| Plxnd1 | Other | plexin D1 | 1.42 | 2.12 | 1.71 |
| Pmepa1 | Other | prostate transmembrane protein, androgen induced 1 | 1.49 | 2.76 | 1.96 |
| Pno1 | Other | partner of NOB1 homolog (S. cerevisiae) | 1.96 | 2.05 | 1.43 |
| Podnl1 | Other | podocan-like 1 | 1.45 | 2.55 | 1.90 |
| Pomp | Other | proteasome maturation protein | 1.84 | 2.04 | 1.25 |
| Pragmin | Other | homolog of rat pragma of Rnd2 | 2.87 | 3.25 | 2.25 |
| Prr13 | Other | proline rich 13 | 1.88 | 2.29 | 1.63 |
| PVR | Other | poliovirus receptor | 2.48 | 3.34 | 3.06 |
| Rassf4 | Other | Ras association (RalGDS/AF-6) domain family member 4 | 3.13 | 6.40 | 3.42 |
| Rftn1 | Other | raftlin, lipid raft linker 1 | 2.82 | 3.95 | 2.99 |
| Rnf145 | Other | ring finger protein 145 | 1.80 | 2.06 | 1.71 |
| Rnf19b | Other | ring finger protein 19B | 2.72 | 4.70 | 3.66 |
| S100a11 | Other | S100 calcium binding protein A11 | 1.91 | 2.49 | 2.00 |
| Sema6b | Other | sema domain, transmembrane domain (TM), and cytoplasmic domain, (semaphorin) 6B | 2.33 | 2.91 | 2.07 |
| Slfn2 | Other | schlafen 2 | 2.66 | 3.68 | 2.48 |
| Slfn3 | Other | schlafen 3 | 3.39 | 5.60 | 1.80 |
| Snf1lk | Other | salt-inducible kinase 1 | 1.75 | 2.42 | 2.15 |
| Sntb1 | Other | syntrophin, beta 1 (dystrophin-associated protein A1, 59kDa, basic component 1) | 1.81 | 2.20 | 1.36 |
| Ssfa2 | Other | sperm specific antigen 2 | 2.24 | 2.66 | 2.37 |
| Tagln2 | Other | transgelin 2 | 2.43 | 3.85 | 3.36 |
| Tax1bp3 | Other | Tax1 (human T-cell leukemia virus type I) binding protein 3 | 1.87 | 2.68 | 2.40 |
| Tbc1d19 | Other | TBC1 domain family, member 19 | 1.57 | 2.10 | 1.88 |
| Tbc1d2 | Other | TBC1 domain family, member 2 | 1.82 | 2.47 | 1.84 |
| Tbc1d24 | Other | TBC1 domain family, member 24 | 2.09 | 2.83 | 2.04 |
| Tes | Other | testis derived transcript (3 LIM domains) | 2.37 | 2.83 | 2.36 |
| Tm4sf19 | Other | transmembrane 4 L six family member 19 | 3.04 | 7.26 | 3.87 |
| Tm6sf1 | Other | transmembrane 6 superfamily member 1 | 2.80 | 3.13 | 2.35 |
| Tmem26 | Other | transmembrane protein 26 | 1.60 | 3.10 | 2.24 |
| Tmem8 | Other | transmembrane protein 8A | 2.11 | 2.25 | 1.73 |
| Tmem86a | Other | transmembrane protein 86A | 1.72 | 2.22 | 1.97 |
| Tspan5 | Other | tetraspanin 5 | 1.89 | 2.18 | 1.84 |
| Ttc9 | Other | tetratricopeptide repeat domain 9 | 2.49 | 5.07 | 3.71 |
| Tusc2 | Other | tumor suppressor candidate 2 | 1.73 | 2.42 | 1.50 |
| Vac14 | Other | Vac14 homolog (S. cerevisiae) | 1.97 | 2.12 | 1.76 |
| Xpr1 | Other | xenotropic and polytropic retrovirus receptor | 3.01 | 3.63 | 2.75 |
| Zdhhc18 | Other | zinc finger, DHHC-type containing 18 | 1.47 | 2.18 | 1.31 |
| Zfand2a | Other | zinc finger, AN1-type domain 2A | 1.61 | 3.57 | 2.72 |
| Zfp334 | Other | zinc finger protein 334 | 2.00 | 4.76 | 2.65 |
| Zswim4 | Other | zinc finger, SWIM-type containing 4 | 1.54 | 2.63 | 1.95 |
| Abcb4 | Transporter | ATP-binding cassette, sub-family B (MDR/TAP), member 4 | 3.41 | 4.22 | 2.79 |
| Abcc4 | Transporter | ATP-binding cassette, sub-family C (CFTR/MRP), member 4 | 2.30 | 3.15 | 2.60 |
| Ap1s1 | Transporter | adaptor-related protein complex 1, sigma 1 subunit | 1.66 | 2.14 | 1.66 |
| Ap2a2 | Transporter | adaptor-related protein complex 2, alpha 2 subunit | 1.80 | 2.05 | 1.74 |
| Arf6 | Transporter | ADP-ribosylation factor 6 | 2.14 | 3.03 | 2.29 |
| Arpp-19 | Transporter | cAMP-regulated phosphoprotein, 19kDa | 2.08 | 2.56 | 1.88 |
| Atp11a | Transporter | ATPase, class VI, type 11A | 1.76 | 2.31 | 1.89 |
| Atp5b | Transporter | ATP synthase, H+ transporting, mitochondrial F1 complex, beta polypeptide | 1.85 | 2.04 | 1.61 |
| Atp5g3 | Transporter | ATP synthase, H+ transporting, mitochondrial F0 complex, subunit C3 (subunit 9) | 2.02 | 2.74 | 1.26 |
| Atp6ap2 | Transporter | ATPase, H+ transporting, lysosomal accessory protein 2 | 2.27 | 3.55 | 2.93 |
| Atp6v0b | Transporter | ATPase, H+ transporting, lysosomal 21kDa, V0 subunit b | 2.10 | 4.37 | 2.39 |
| Atp6v0c | Transporter | ATPase, H+ transporting, lysosomal 16kDa, V0 subunit c | 1.56 | 2.42 | 1.60 |
| Atp6v0d2 | Transporter | ATPase, H+ transporting, lysosomal 38kDa, V0 subunit d2 | 4.45 | 7.25 | 4.52 |
| Atp6v0e1 | Transporter | ATPase, H+ transporting, lysosomal 9kDa, V0 subunit e1 | 1.36 | 2.05 | 1.38 |
| Atp6v1a | Transporter | ATPase, H+ transporting, lysosomal 70kDa, V1 subunit A | 2.18 | 3.77 | 2.92 |
| Atp6v1b2 | Transporter | ATPase, H+ transporting, lysosomal 56/58kDa, V1 subunit B2 | 3.01 | 4.48 | 3.61 |
| Atp6v1c1 | Transporter | ATPase, H+ transporting, lysosomal 42kDa, V1 subunit C1 | 2.31 | 3.12 | 2.13 |
| Atp6v1d | Transporter | ATPase, H+ transporting, lysosomal 34kDa, V1 subunit D | 2.14 | 2.80 | 2.47 |
| Atp6v1f | Transporter | ATPase, H+ transporting, lysosomal 14kDa, V1 subunit F | 1.49 | 2.29 | 1.55 |
| Atp6v1h | Transporter | ATPase, H+ transporting, lysosomal 50/57kDa, V1 subunit H | 2.01 | 2.65 | 2.03 |
| Cacna1a | Transporter | calcium channel, voltage-dependent, P/Q type, alpha 1A subunit | 1.62 | 2.17 | 1.52 |
| Clcn7 | Transporter | chloride channel 7 | 2.47 | 3.81 | 2.74 |
| Clic1 | Transporter | chloride intracellular channel 1 | 2.54 | 2.76 | 2.28 |
| Fabp5 | Transporter | fatty acid binding protein 5 (psoriasis-associated) | 1.89 | 2.58 | 1.61 |
| Fxyd2 | Transporter | FXYD domain containing ion transport regulator 2 | 1.83 | 3.50 | 3.06 |
| Kcnj2 | Transporter | potassium inwardly-rectifying channel, subfamily J, member 2 | 2.61 | 3.28 | 2.84 |
| Kcnn4 | Transporter | potassium intermediate/small conductance calcium-activated channel, subfamily N, member 4 | 3.19 | 4.28 | 2.87 |
| Kctd12 | Transporter | potassium channel tetramerisation domain containing 12 | 1.56 | 2.59 | 1.97 |
| Ldlr | Transporter | low density lipoprotein receptor | 1.44 | 2.26 | 1.46 |
| M6pr | Transporter | mannose-6-phosphate receptor (cation dependent) | 1.66 | 2.40 | 1.80 |
| Mcoln3 | Transporter | mucolipin 3 | 2.03 | 3.74 | 2.80 |
| Mfsd1 | Transporter | major facilitator superfamily domain containing 1 | 3.74 | 4.23 | 2.83 |
| RT1-CE1 | Transporter | RT1-CE5 RT1 class I, locus CE1 | 1.63 | 2.30 | 1.70 |
| RT1-CE5 | Transporter | RT1-CE5 RT1 class I, locus CE5 | 1.63 | 2.15 | 1.64 |
| Slc12a7 | Transporter | solute carrier family 12 (potassium/chloride transporters), member 7 | 1.61 | 2.32 | 1.67 |
| Slc16a7 | Transporter | solute carrier family 16, member 7 (monocarboxylic acid transporter 2) | 1.84 | 2.08 | 1.91 |
| Slc1a5 | Transporter | solute carrier family 1 (neutral amino acid transporter), member 5 | 2.11 | 2.40 | 1.63 |
| Slc28a2 | Transporter | solute carrier family 28 (sodium-coupled nucleoside transporter), member 2 | 2.68 | 3.35 | 2.88 |
| Slc35e4 | Transporter | solute carrier family 35, member E4 | 1.49 | 3.91 | 3.06 |
| Slc37a2 | Transporter | solute carrier family 37 (glycerol-3-phosphate transporter), member 2 | 3.31 | 6.05 | 3.36 |
| Slc38a6 | Transporter | solute carrier family 38, member 6 | 2.09 | 2.62 | 2.17 |
| Slc38a7 | Transporter | solute carrier family 38, member 7 | 1.63 | 2.14 | 1.88 |
| Slc4a2 | Transporter | solute carrier family 4, anion exchanger, member 2 (erythrocyte membrane protein band 3-like 1) | 2.17 | 3.65 | 2.23 |
| Slc6a6 | Transporter | solute carrier family 6 (neurotransmitter transporter, taurine), member 6 | 1.54 | 2.12 | 1.73 |
| Slc6a8 | Transporter | solute carrier family 6 (neurotransmitter transporter, creatine), member 8 | 1.96 | 3.90 | 2.45 |
| Slc7a5 | Transporter | solute carrier family 7 (cationic amino acid transporter, y+ system), member 5 | 2.33 | 3.01 | 1.69 |
| Slc7a8 | Transporter | solute carrier family 7 (cationic amino acid transporter, y+ system), member 8 | 2.43 | 2.78 | 1.65 |
| Snx10 | Transporter | sorting nexin 10 | 3.22 | 4.68 | 3.74 |
| Snx8 | Transporter | sorting nexin 8 | 2.08 | 2.87 | 2.50 |
| Stard3 | Transporter | StAR-related lipid transfer (START) domain containing 3 | 1.70 | 2.09 | 1.46 |
| Stx11 | Transporter | syntaxin 11 | 1.66 | 2.48 | 1.78 |
| Syngr1 | Transporter | synaptogyrin 1 | 1.84 | 2.13 | 1.67 |
| Tap1 | Transporter | transporter 1, ATP-binding cassette, sub-family B (MDR/TAP) | 1.70 | 2.09 | 1.61 |
| Trpv2 | Transporter | transient receptor potential cation channel, subfamily V, member 2 | 3.66 | 5.67 | 3.47 |
| Ttyh2 | Transporter | tweety homolog 2 (Drosophila) | 2.69 | 5.11 | 3.73 |
| Ttyh3 | Transporter | tweety homolog 3 (Drosophila) | 2.21 | 2.76 | 1.82 |
