# Supplementary material for: Sequential Alterations in Catabolic and Anabolic Gene Expression Parallel Pathological Changes during Progression of Monoiodoacetate-Induced Arthritis
Source: PLoS One. 2011 Sep 13;6(9):e24320. doi: 10.1371/journal.pone.0024320 (PMC3172226; doi:10.1371/journal.pone.0024320)
Supplement: Table S4 — Changes in the expression of genes in Cluster V. Please see Table S1 for group description. (DOC) [file pone.0024320.s005.doc]

**Table S4**. Changes in the expression of genes in *Cluster V*. Please see Table S1 for group description.

| Gene | Group | Description | MIA5 | MIA9 | MIA21 |
| --- | --- | --- | --- | --- | --- |
| Ccnd2 | CD | cyclin D2 | -2.95 | -2.71 | -1.83 |
| Cdkn1c | CD | cyclin-dependent kinase inhibitor 1C (p57, Kip2) | -4.93 | -5.79 | -3.44 |
| Dock9 | CD | dedicator of cytokinesis 9 | -1.18 | -2.63 | -1.66 |
| Enox1 | CD | ecto-NOX disulfide-thiol exchanger 1 | -2.14 | -2.48 | -1.61 |
| Gli1 | CD | GLI family zinc finger 1 | -9.48 | -9.36 | -6.03 |
| Gli2 | CD | GLI family zinc finger 2 | -2.70 | -3.29 | -2.86 |
| Hoxa5 | CD | homeobox A5 | -2.80 | -3.30 | -2.39 |
| Hoxc5 | CD | homeobox C5 | -2.29 | -2.42 | -2.47 |
| kif13B | CD | kinesin family member 13B | -2.64 | -3.14 | -1.99 |
| Klf2 | CD | Kruppel-like factor 2 (lung) | -2.46 | -2.25 | -1.44 |
| Klf9 | CD | Kruppel-like factor 9 | -2.01 | -2.07 | 1.27 |
| Neo1 | CD | neogenin homolog 1 (chicken) | -1.79 | -2.76 | -1.97 |
| Pdcd4 | CD | programmed cell death 4 (neoplastic transformation inhibitor) | -2.19 | -2.90 | -1.74 |
| Prdm5 | CD | PR domain containing 5 | -2.83 | -4.00 | -2.50 |
| Tpd52l1 | CD | tumor protein D52-like 1 | -3.22 | -3.94 | -3.55 |
| Tspan6 | CD | tetraspanin 6 | -2.11 | -2.48 | -2.25 |
| Twsg1 | CD | twisted gastrulation homolog 1 (Drosophila) | -2.43 | -2.74 | -1.44 |
| Zbtb16 | CD | zinc finger and BTB domain containing 16 | -3.48 | -5.01 | -1.79 |
| Chad | ECM | chondroadherin | -18.26 | -32.62 | -7.11 |
| Col10a1 | ECM | collagen, type X, alpha 1 | -28.99 | -83.31 | -45.46 |
| Col11a1 | ECM | collagen, type XI, alpha 1 | -3.78 | -5.72 | -2.98 |
| Col11a2 | ECM | collagen, type XI, alpha 2 | -6.29 | -16.93 | -9.95 |
| Col24a1 | ECM | collagen, type XXIV, alpha 1 | -1.97 | -2.51 | -1.52 |
| Col2a1 | ECM | collagen, type II, alpha 1 | -14.95 | -50.35 | -9.06 |
| Col9a1 | ECM | collagen, type IX, alpha 1 | -15.31 | -76.98 | -46.07 |
| Col9a2 | ECM | collagen, type IX, alpha 2 | -13.48 | -27.61 | -22.39 |
| Col9a3 | ECM | collagen, type IX, alpha 3 | -9.38 | -18.95 | -12.57 |
| Fbln5 | ECM | fibulin 5 | -2.54 | -3.08 | -1.66 |
| Hapln1 | ECM | hyaluronan and proteoglycan link protein 1 | -11.01 | -19.03 | -7.55 |
| Hs3st1 | ECM | heparan sulfate (glucosamine) 3-O-sulfotransferase 1 | -6.09 | -6.25 | -7.69 |
| Hs3st3b1 | ECM | heparan sulfate (glucosamine) 3-O-sulfotransferase 3B1 | -7.06 | -7.37 | -7.83 |
| Krt8 | ECM | keratin 8 | -3.55 | -4.43 | -4.05 |
| Matn1 | ECM | matrilin 1, cartilage matrix protein | -3.86 | -4.00 | -3.98 |
| Matn3 | ECM | matrilin 3 | -26.27 | -98.06 | -74.60 |
| Mgp | ECM | matrix Gla protein | -4.72 | -5.64 | -3.28 |
| Myl3 | ECM | myosin, light chain 3, alkali; ventricular, skeletal, slow | -6.32 | -6.34 | -5.87 |
| Omd | ECM | osteomodulin | -11.50 | -12.99 | -3.35 |
| Prg4 | ECM | proteoglycan 4 | -7.62 | -7.66 | -3.53 |
| Tnnc1 | ECM | troponin C type 1 (slow) | -2.66 | -3.39 | -3.33 |
| Vit | ECM | vitrin | -13.37 | -15.06 | -10.92 |
| Adamts3 | ECM2 | ADAM metallopeptidase with thrombospondin type 1 motif, 3 | -3.32 | -3.91 | -4.69 |
| Adamts6 | ECM2 | ADAM metallopeptidase with thrombospondin type 1 motif, 6 | -2.15 | -3.05 | -2.36 |
| Adamtsl3 | ECM2 | ADAMTS-like 3 | -3.14 | -4.11 | -2.36 |
| Alpl | ECM2 | alkaline phosphatase, liver/bone/kidney | -2.80 | -2.83 | -1.26 |
| Arsg | ECM2 | arylsulfatase G | -2.19 | -2.14 | -1.56 |
| Chsy3 | ECM2 | chondroitin sulfate synthase 3 | -2.58 | -2.60 | -2.06 |
| Has2 | ECM2 | hyaluronan synthase 2 | -1.66 | -2.29 | -3.28 |
| Leprel1 | ECM2 | leprecan-like 1 | -17.59 | -23.95 | -6.98 |
| Mmp16 | ECM2 | matrix metallopeptidase 16 (membrane-inserted) | -1.37 | -2.20 | -1.13 |
| Bglap | GF | bone gamma-carboxyglutamate (gla) protein | -1.38 | -3.52 | -1.61 |
| Bmp3 | GF | bone morphogenetic protein 3 | -1.12 | -3.34 | -1.36 |
| Bmp5 | GF | bone morphogenetic protein 5 | -2.50 | -4.75 | -2.59 |
| Bmpr1a | GF | bone morphogenetic protein receptor, type IA | -2.01 | -2.46 | -1.53 |
| Ctgf | GF | connective tissue growth factor | -2.79 | -5.24 | -1.68 |
| Egf | GF | epidermal growth factor (beta-urogastrone) | -2.11 | -2.37 | -2.04 |
| Fgf2 | GF | fibroblast growth factor 2 | -4.40 | -5.85 | -4.53 |
| Fgfr2 | GF | fibroblast growth factor receptor 2 | -2.77 | -5.61 | -3.06 |
| Fgfr3 | GF | fibroblast growth factor receptor 3 | -5.67 | -7.94 | -5.77 |
| Ghr | GF | growth hormone receptor | -3.19 | -4.24 | -2.37 |
| Grem1 | GF | gremlin 1, cysteine knot superfamily, homolog (Xenopus laevis) | -7.15 | -8.86 | -12.40 |
| Pdgfrl | GF | platelet-derived growth factor receptor-like | -4.73 | -4.58 | -2.63 |
| Tgfb3 | GF | transforming growth factor, beta 3 | -1.75 | -2.65 | -1.25 |
| Tgfbr3 | GF | transforming growth factor, beta receptor III | -2.32 | -3.55 | -1.74 |
| Bambi | GF2 | BMP and activin membrane-bound inhibitor homolog (Xenopus laevis) | -1.76 | -2.83 | -1.98 |
| Ctnnal1 | GF2 | catenin (cadherin-associated protein), alpha-like 1 | -1.33 | -2.21 | -1.50 |
| Daam2 | GF2 | dishevelled associated activator of morphogenesis 2 | -3.06 | -3.46 | -1.39 |
| Dlx5 | GF2 | distal-less homeobox 5 | -2.52 | -3.73 | -2.46 |
| Dmp1 | GF2 | dentin matrix acidic phosphoprotein 1 | -1.17 | -3.10 | -1.99 |
| Frzb | GF2 | frizzled-related protein | -47.41 | -52.72 | -49.61 |
| Fzd9 | GF2 | frizzled homolog 9 (Drosophila) | -8.60 | -8.90 | -6.65 |
| Grb14 | GF2 | growth factor receptor-bound protein 14 | -1.40 | -2.27 | -2.11 |
| Igfbp5 | GF2 | insulin-like growth factor binding protein 5 | -2.29 | -3.04 | -1.33 |
| Ihh | GF2 | Indian hedgehog homolog (Drosophila) | -4.63 | -5.24 | -4.05 |
| Irs1 | GF2 | insulin receptor substrate 1 | -1.97 | -2.99 | -1.84 |
| Pthr1 | GF2 | parathyroid hormone 1 receptor | -2.47 | -2.47 | -1.71 |
| Sfrp5 | GF2 | secreted frizzled-related protein 5 | -9.76 | -11.77 | -11.38 |
| Smad3 | GF2 | SMAD family member 3 | -2.11 | -2.41 | -1.60 |
| Smad9 | GF2 | SMAD family member 9 | -1.32 | -2.34 | -1.32 |
| Sox6 | GF2 | SRY (sex determining region Y)-box 6 | -3.91 | -5.87 | -3.02 |
| Sparc | GF2 | secreted protein, acidic, cysteine-rich (osteonectin) | -2.06 | -1.92 | -1.37 |
| Stc2 | GF2 | stanniocalcin 2 | -6.02 | -6.43 | -4.68 |
| Vdr | GF2 | vitamin D (1,25- dihydroxyvitamin D3) receptor | -1.21 | -2.72 | -2.78 |
| Wif1 | GF2 | WNT inhibitory factor 1 | -13.04 | -15.20 | -6.43 |
| Wisp3 | GF2 | WNT1 inducible signaling pathway protein 3 | -3.69 | -3.91 | -3.73 |
| Clec3a | Inf | C-type lectin domain family 3, member A | -54.66 | -143.97 | -20.74 |
| Cmtm5 | Inf | CKLF-like MARVEL transmembrane domain containing 5 | -4.23 | -5.28 | -5.48 |
| Cxcl14 | Inf | chemokine (C-X-C motif) ligand 14 | -1.61 | -1.98 | -2.12 |
| Il11ra1 | Inf | interleukin 11 receptor, alpha | -1.92 | -2.07 | -1.39 |
| Il16 | Inf | interleukin 16 (lymphocyte chemoattractant factor) | -3.78 | -4.52 | -1.68 |
| Il17b | Inf | interleukin 17B | -2.84 | -3.32 | -2.93 |
| Il17rb | Inf | interleukin 17 receptor B | -1.85 | -1.98 | -2.14 |
| Lect1 | Inf | leukocyte cell derived chemotaxin 1 | -8.32 | -34.44 | -29.87 |
| Lifr | Inf | leukemia inhibitory factor receptor alpha | -1.22 | -2.10 | -1.51 |
| Mia | Inf | melanoma inhibitory activity | -21.70 | -43.76 | -38.49 |
| Pcdh9 | Inf | protocadherin 9 | -3.77 | -3.97 | -3.05 |
| Vav3 | Inf | vav 3 guanine nucleotide exchange factor | -1.98 | -2.16 | -1.87 |
| C1qtnf7 | Inf2 | C1q and tumor necrosis factor related protein 7 | -3.90 | -4.72 | -2.43 |
| Cd109 | Inf2 | CD109 molecule | -2.53 | -2.64 | -1.35 |
| Cd24 | Inf2 | CD24 molecule | -2.85 | -4.44 | -4.60 |
| Cyr61 | Inf2 | cysteine-rich, angiogenic inducer, 61 | -3.16 | -3.27 | -2.34 |
| Epha4 | Inf2 | EPH receptor A4 | -3.33 | -3.44 | -2.33 |
| Erg | Inf2 | v-ets erythroblastosis virus E26 oncogene homolog (avian) | -2.21 | -2.29 | -1.77 |
| F13a1 | Inf2 | coagulation factor XIII, A1 polypeptide | -4.97 | -6.11 | -2.59 |
| F5 | Inf2 | coagulation factor V (proaccelerin, labile factor) | -1.77 | -2.35 | -2.64 |
| Fkbp9 | Inf2 | FK506 binding protein 9, 63 kDa | -2.40 | -2.50 | -1.81 |
| Hrsp12 | Inf2 | heat-responsive protein 12 | -1.72 | -2.29 | -1.59 |
| Ifitm5 | Inf2 | interferon induced transmembrane protein 5 | -1.40 | -2.41 | -1.27 |
| Itfg2 | Inf2 | integrin alpha FG-GAP repeat containing 2 | -1.85 | -2.19 | -1.91 |
| Itga10 | Inf2 | integrin, alpha 10 | -6.49 | -7.25 | -3.14 |
| Itga6 | Inf2 | integrin, alpha 6 | -2.08 | -2.25 | -1.83 |
| Itgb8 | Inf2 | integrin, beta 8 | -1.11 | -1.18 | 2.22 |
| Itgbl1 | Inf2 | integrin, beta-like 1 (with EGF-like repeat domains) | -6.39 | -10.73 | -2.57 |
| Lpar4 | Inf2 | lysophosphatidic acid receptor 4 | -6.56 | -8.22 | -6.30 |
| Ly75 | Inf2 | lymphocyte antigen 75 | -1.29 | -1.43 | -2.53 |
| Mllt3 | Inf2 | myeloid/lymphoid or mixed-lineage leukemia (trithorax homolog, Drosophila); translocated to, 3 | -2.36 | -2.80 | -1.98 |
| Nfia | Inf2 | nuclear factor I/A | -2.17 | -2.78 | -1.82 |
| Nfib | Inf2 | nuclear factor I/B | -1.95 | -2.45 | -1.81 |
| Nrk | Inf2 | Nik related kinase | -16.44 | -18.71 | -16.18 |
| Pcdh18 | Inf2 | protocadherin 18 | -1.53 | -3.10 | -1.60 |
| Plcg2 | Inf2 | phospholipase C, gamma 2 (phosphatidylinositol-specific) | -2.02 | -2.10 | -2.18 |
| Prkd1 | Inf2 | protein kinase D1 | -2.01 | -2.65 | -1.53 |
| Prkg2 | Inf2 | protein kinase, cGMP-dependent, type II | -5.69 | -10.57 | -8.07 |
| Ptgis | Inf2 | prostaglandin I2 (prostacyclin) synthase | -2.78 | -6.20 | -2.46 |
| Ralbp1 | Inf2 | ralA binding protein 1 | -4.23 | -5.57 | -5.32 |
| Rasd1 | Inf2 | RAS, dexamethasone-induced 1 | -2.17 | -3.34 | -2.17 |
| RragB | Inf2 | Ras-related GTP binding B | -2.37 | -2.18 | -1.33 |
| Scara3 | Inf2 | scavenger receptor class A, member 3 | -2.16 | -2.44 | -1.30 |
| Stk32b | Inf2 | serine/threonine kinase 32B | -9.83 | -9.94 | -8.83 |
| Tll1 | Inf2 | tolloid-like 1 | -4.73 | -6.51 | -5.36 |
| Trib2 | Inf2 | tribbles homolog 2 (Drosophila) | -2.81 | -2.87 | -2.56 |
| Vof16 | Inf2 | ischemia related factor vof-16 | -1.90 | -2.46 | -1.67 |
| A2m | Meta | alpha-2-macroglobulin | -1.56 | -2.36 | -3.59 |
| Adarb1 | Meta | adenosine deaminase, RNA-specific, B1 (RED1 homolog rat) | -3.45 | -3.45 | -2.27 |
| Adcy2 | Meta | adenylate cyclase 2 (brain) | -3.06 | -4.37 | -3.57 |
| Adhfe1 | Meta | alcohol dehydrogenase, iron containing, 1 | -3.97 | -4.46 | -3.83 |
| Aldh6a1 | Meta | aldehyde dehydrogenase 6 family, member A1 | -2.50 | -3.29 | -1.80 |
| Ampd3 | Meta | adenosine monophosphate deaminase (isoform E) | -1.49 | -1.90 | -2.38 |
| Ankrd2 | Meta | ankyrin repeat domain 2 (stretch responsive muscle) | -3.26 | -3.49 | -2.96 |
| Apeg3 | Meta | antisense paternally expressed gene 3 | -4.11 | -4.31 | -3.76 |
| Ar | Meta | androgen receptor | -1.47 | -2.59 | -1.59 |
| Arap2 | Meta | ArfGAP with RhoGAP domain, ankyrin repeat and PH domain 2 | -1.16 | -2.06 | -2.72 |
| Argbp2 | Meta | sorbin and SH3 domain containing 2 | -2.61 | -3.22 | -2.49 |
| Asrgl1 | Meta | asparaginase like 1 | -1.95 | -3.84 | -3.54 |
| B3galt1 | Meta | UDP-Gal:betaGlcNAc beta 1,3-galactosyltransferase, polypeptide 1 | -2.83 | -3.01 | -1.78 |
| B4galnt3 | Meta | beta-1,4-N-acetyl-galactosaminyl transferase 3 | -5.33 | -7.41 | -3.64 |
| Calml3 | Meta | calmodulin-like 3 | -2.86 | -3.39 | -2.86 |
| Capn6 | Meta | calpain 6 | -11.87 | -19.16 | -8.77 |
| Car11 | Meta | carbonic anhydrase XI | -2.34 | -2.65 | -1.80 |
| Car12 | Meta | carbonic anhydrase XII | -3.14 | -4.15 | -1.92 |
| Cdo1 | Meta | cysteine dioxygenase, type I | -1.77 | -2.68 | 1.51 |
| Chdh | Meta | choline dehydrogenase | -2.58 | -3.26 | -3.12 |
| Clk1 | Meta | CDC-like kinase 1 | -1.41 | -2.16 | -1.60 |
| Cpvl | Meta | carboxypeptidase, vitellogenic-like | -2.16 | -2.36 | -2.22 |
| Cpz | Meta | carboxypeptidase Z | -4.31 | -4.92 | -3.60 |
| Cryab | Meta | crystallin, alpha B | -3.69 | -4.60 | -4.26 |
| Cth | Meta | cystathionase (cystathionine gamma-lyase) | -2.25 | -2.32 | -2.81 |
| Dhtkd1 | Meta | dehydrogenase E1 and transketolase domain containing 1 | -2.61 | -2.96 | -2.19 |
| Dlk1 | Meta | delta-like 1 homolog (Drosophila) | -10.80 | -12.69 | -12.16 |
| Dmpk | Meta | dystrophia myotonica-protein kinase | -2.44 | -2.98 | -1.63 |
| Dync1i1 | Meta | dynein, cytoplasmic 1, intermediate chain 1 | -2.21 | -2.76 | -2.74 |
| Ehhadh | Meta | enoyl-Coenzyme A, hydratase/3-hydroxyacyl Coenzyme A dehydrogenase | -1.91 | -2.09 | -1.76 |
| Elovl6 | Meta | ELOVL family member 6, elongation of long chain fatty acids (FEN1/Elo2, SUR4/Elo3-like, yeast) | -1.41 | -1.66 | -2.02 |
| Enpp6 | Meta | ectonucleotide pyrophosphatase/phosphodiesterase 6 | -1.31 | -2.43 | -1.95 |
| Epas1 | Meta | endothelial PAS domain protein 1 | -2.15 | -2.36 | -1.95 |
| Epm2a | Meta | epilepsy, progressive myoclonic epilepsy, type 2 gene alpha | -1.87 | -2.44 | -1.64 |
| Ercc6 | Meta | excision repair cross-complementing rodent repair deficiency, complementation group 6 | -1.71 | -2.07 | -1.75 |
| Etnk2 | Meta | ethanolamine kinase 2 | -3.01 | -3.15 | -3.15 |
| Extl1 | Meta | exostoses (multiple)-like 1 | -8.50 | -9.42 | -9.95 |
| Fam38b | Meta | family with sequence similarity 38, member B | -5.23 | -5.99 | -3.75 |
| Farp1 | Meta | FERM, RhoGEF (ARHGEF) and pleckstrin domain protein 1 (chondrocyte-derived) | -2.56 | -3.42 | -2.36 |
| Foxa3 | Meta | forkhead box A3 | -2.12 | -2.19 | -2.18 |
| Foxp2 | Meta | forkhead box P2 | -1.25 | -2.98 | -2.53 |
| Galnt5 | Meta | UDP-N-acetyl-alpha-D-galactosamine:polypeptide N-acetylgalactosaminyltransferase 5 (GalNAc-T5) | -2.86 | -3.22 | -2.19 |
| Gfpt1 | Meta | glutamine-fructose-6-phosphate transaminase 1 | -2.12 | -2.32 | -1.76 |
| Glt25d2 | Meta | glycosyltransferase 25 domain containing 2 | -27.76 | -29.69 | -10.03 |
| Gmpr | Meta | guanosine monophosphate reductase | -3.07 | -3.57 | -3.88 |
| Gnai1 | Meta | guanine nucleotide binding protein (G protein), alpha inhibiting activity polypeptide 1 | -1.26 | -2.00 | -1.28 |
| Gpsm2 | Meta | G-protein signaling modulator 2 (AGS3-like, C. elegans) | -3.07 | -3.12 | -2.24 |
| Gpt2 | Meta | glutamic pyruvate transaminase (alanine aminotransferase) 2 | -2.03 | -2.29 | -2.07 |
| Grhpr | Meta | glyoxylate reductase/hydroxypyruvate reductase | -1.93 | -2.08 | -1.72 |
| Grk5 | Meta | G protein-coupled receptor kinase 5 | -4.23 | -4.09 | -2.99 |
| Gstm7 | Meta | glutathione S-transferase, mu 7 | -2.51 | -2.94 | -2.78 |
| Hdac9 | Meta | histone deacetylase 9 | -1.86 | -2.05 | -2.01 |
| Hhip | Meta | hedgehog interacting protein | -8.58 | -8.44 | -4.47 |
| Hmgcll1 | Meta | 3-hydroxymethyl-3-methylglutaryl-Coenzyme A lyase-like 1 | -3.55 | -4.59 | -2.77 |
| Hmgn3 | Meta | high mobility group nucleosomal binding domain 3 | -2.53 | -2.65 | -1.79 |
| Inpp1 | Meta | inositol polyphosphate-1-phosphatase | -2.41 | -2.42 | -1.88 |
| Itm2a | Meta | integral membrane protein 2A | -8.03 | -9.27 | -5.06 |
| Lims2 | Meta | LIM and senescent cell antigen-like domains 2 | -17.66 | -17.81 | -8.32 |
| Loxl3 | Meta | lysyl oxidase-like 3 | -4.71 | -4.67 | -2.78 |
| Loxl4 | Meta | lysyl oxidase-like 4 | -3.84 | -5.43 | -3.21 |
| Lpin1 | Meta | lipin 1 | -2.19 | -3.51 | -2.20 |
| Lrrc16a | Meta | leucine rich repeat containing 16A | -3.05 | -3.58 | -2.64 |
| Magi2 | Meta | membrane associated guanylate kinase, WW and PDZ domain containing 2 | -1.57 | -2.25 | -1.81 |
| Man1a1 | Meta | mannosidase, alpha, class 1A, member 1 | -2.29 | -2.57 | -1.99 |
| Mboat2 | Meta | membrane bound O-acyltransferase domain containing 2 | -2.46 | -2.67 | -2.44 |
| Msi1 | Meta | musashi homolog 1 (Drosophila) | -1.66 | -1.86 | -2.02 |
| Mst4 | Meta | serine/threonine protein kinase MST4 | -5.63 | -7.09 | -5.62 |
| Mtr | Meta | 5-methyltetrahydrofolate-homocysteine methyltransferase | -1.58 | -2.38 | -1.72 |
| Mtus1 | Meta | mitochondrial tumor suppressor 1 | -2.73 | -2.62 | -1.90 |
| Nebl | Meta | nebulette | -2.64 | -4.53 | -3.31 |
| Ninj2 | Meta | ninjurin 2 | -1.93 | -2.05 | -2.01 |
| Npr2 | Meta | natriuretic peptide receptor B/guanylate cyclase B (atrionatriuretic peptide receptor B) | -2.18 | -2.09 | -1.32 |
| Nrbp2 | Meta | nuclear receptor binding protein 2 | -2.56 | -3.50 | -1.94 |
| Obsl1 | Meta | obscurin-like 1 | -2.84 | -3.03 | -2.40 |
| Papss1 | Meta | 3'-phosphoadenosine 5'-phosphosulfate synthase 1 | -2.00 | -2.13 | -1.85 |
| Parp8 | Meta | poly (ADP-ribose) polymerase family, member 8 | -3.81 | -4.25 | -3.19 |
| Pc | Meta | pyruvate carboxylase | -2.50 | -2.82 | -2.29 |
| Pcsk6 | Meta | proprotein convertase subtilisin/kexin type 6 | -4.89 | -7.02 | -3.27 |
| Pde7b | Meta | phosphodiesterase 7B | -1.59 | -3.10 | -1.92 |
| Pdzd2 | Meta | PDZ domain containing 2 | -3.64 | -4.45 | -3.24 |
| Per1 | Meta | period homolog 1 (Drosophila) | -2.04 | -2.86 | -1.37 |
| Per3 | Meta | period homolog 3 (Drosophila) | -1.72 | -10.78 | -3.81 |
| Pfkm | Meta | phosphofructokinase, muscle | -2.32 | -2.25 | -1.49 |
| Plcb1 | Meta | phospholipase C, beta 1 (phosphoinositide-specific) | -2.15 | -2.20 | -1.26 |
| Pon3 | Meta | paraoxonase 3 | -2.17 | -2.33 | -1.23 |
| Prkcz | Meta | protein kinase C, zeta | -2.68 | -2.83 | -1.77 |
| Prrg1 | Meta | proline rich Gla (G-carboxyglutamic acid) 1 | -1.57 | -2.05 | -1.37 |
| Rab4b | Meta | RAB4B, member RAS oncogene family | -2.09 | -2.20 | -2.08 |
| Rbm8 | Meta | RNA binding motif protein 8A | -2.97 | -3.31 | -3.38 |
| Rhoh | Meta | ras homolog gene family, member H | -1.12 | -1.97 | -2.83 |
| Robo1 | Meta | roundabout, axon guidance receptor, homolog 1 (Drosophila) | -1.49 | -2.07 | -1.45 |
| Robo2 | Meta | roundabout, axon guidance receptor, homolog 2 (Drosophila) | -1.17 | -2.38 | -1.80 |
| Rps6ka5 | Meta | ribosomal protein S6 kinase, 90kDa, polypeptide 5 | -1.61 | -3.15 | -2.24 |
| Rtn1 | Meta | reticulon 1 | -3.93 | -4.05 | -4.17 |
| Scin | Meta | scinderin | -22.16 | -24.43 | -11.75 |
| Sgms2 | Meta | sphingomyelin synthase 2 | -1.90 | -2.65 | -1.70 |
| Smarca1 | Meta | SWI/SNF related, matrix associated, actin dependent regulator of chromatin, subfamily a, member 1 | -2.90 | -3.66 | -2.57 |
| Smpd3 | Meta | sphingomyelin phosphodiesterase 3, neutral membrane (neutral sphingomyelinase II) | -6.38 | -12.61 | -5.69 |
| Sptlc3 | Meta | serine palmitoyltransferase, long chain base subunit 3 | -15.10 | -15.63 | -15.27 |
| Ssbp2 | Meta | single-stranded DNA binding protein 2 | -1.56 | -2.09 | -1.31 |
| Sstr1 | Meta | somatostatin receptor 1 | -1.91 | -2.06 | -2.00 |
| St3gal6 | Meta | ST3 beta-galactoside alpha-2,3-sialyltransferase 6 | -1.57 | -2.98 | -2.86 |
| Stard10 | Meta | StAR-related lipid transfer (START) domain containing 10 | -2.59 | -2.76 | -2.11 |
| Strbp | Meta | spermatid perinuclear RNA binding protein | -1.73 | -2.68 | -2.77 |
| Tef | Meta | thyrotrophic embryonic factor | -1.34 | -3.96 | -1.75 |
| Tenc1 | Meta | tensin like C1 domain containing phosphatase (tensin 2) | -2.51 | -2.59 | -1.74 |
| tGap1 | Meta | GTPase activating protein testicular GAP1 | -3.20 | -3.24 | -3.77 |
| Tsc22d4 | Meta | TSC22 domain family, member 4 | -1.94 | -2.08 | -2.23 |
| Tspan13 | Meta | tetraspanin 13 | -1.86 | -2.21 | -1.98 |
| Tst | Meta | thiosulfate sulfurtransferase (rhodanese) | -1.81 | -2.04 | -2.37 |
| Ttc8 | Meta | tetratricopeptide repeat domain 8 | -1.61 | -2.00 | -1.42 |
| Unc5c | Meta | unc-5 homolog C (C. elegans) | -1.64 | -3.23 | -2.15 |
| Usp53 | Meta | ubiquitin specific peptidase 53 | -6.49 | -10.23 | -6.54 |
| Vkorc1 | Meta | vitamin K epoxide reductase complex, subunit 1 | -2.82 | -2.65 | -1.56 |
| Wbscr17 | Meta | Williams-Beuren syndrome chromosome region 17 | -10.56 | -12.70 | -12.01 |
| Wnk4 | Meta | WNK lysine deficient protein kinase 4 | -2.77 | -3.08 | -2.61 |
| Wwox | Meta | WW domain containing oxidoreductase | -1.97 | -2.18 | -1.81 |
| Wwp2 | Meta | WW domain containing E3 ubiquitin protein ligase 2 | -3.59 | -3.72 | -2.78 |
| Yap1 | Meta | Yes-associated protein 1, 65kDa | -2.49 | -2.81 | -1.95 |
| Zpbp | Meta | zona pellucida binding protein | -1.86 | -2.51 | -2.48 |
| Zrsr2 | Meta | zinc finger (CCCH type), RNA-binding motif and serine/arginine rich 2 | -2.10 | -3.13 | -2.54 |
| Aff2 | Other | AF4/FMR2 family, member 2 | -5.30 | -5.71 | -4.40 |
| Aff3 | Other | AF4/FMR2 family, member 3 | -2.79 | -2.77 | -2.04 |
| Agbl3 | Other | ATP/GTP binding protein-like 3 | -2.05 | -2.10 | -1.88 |
| Apbb2 | Other | amyloid beta (A4) precursor protein-binding, family B, member 2 | -1.99 | -2.44 | -1.87 |
| Auts2 | Other | autism susceptibility candidate 2 | -2.12 | -3.22 | -3.07 |
| Auts2l | Other | autism susceptibility candidate 2-like | -2.31 | -3.51 | -4.08 |
| Bhlhe41 | Other | basic helix-loop-helix family, member e41 | -3.18 | -4.88 | -2.34 |
| Btbd3 | Other | BTB (POZ) domain containing 3 | -2.04 | -2.81 | -2.05 |
| Casc4 | Other | cancer susceptibility candidate 4 | -1.85 | -2.37 | -1.30 |
| Ccdc136 | Other | coiled-coil domain containing 136 | -2.61 | -2.81 | -2.46 |
| Ccdc62 | Other | coiled-coil domain containing 62 | -1.69 | -2.21 | -1.56 |
| Cep70 | Other | centrosomal protein 70kDa | -1.16 | -1.82 | -2.17 |
| Clmn | Other | calmin (calponin-like, transmembrane) | -4.65 | -4.68 | -4.65 |
| Cntfr | Other | ciliary neurotrophic factor receptor | -3.25 | -3.31 | -2.85 |
| Coro6 | Other | coronin 6 | -1.86 | -2.91 | -2.30 |
| Crebl2 | Other | cAMP responsive element binding protein-like 2 | -1.55 | -2.00 | -1.29 |
| Crispld1 | Other | cysteine-rich secretory protein LCCL domain containing 1 | -52.24 | -79.92 | -15.42 |
| Ctdspl | Other | CTD (carboxy-terminal domain, RNA polymerase II, polypeptide A) small phosphatase-like | -1.83 | -2.46 | -2.15 |
| Dact1 | Other | dapper, antagonist of beta-catenin, homolog 1 (Xenopus laevis) | -2.84 | -4.15 | -3.03 |
| Dbp | Other | D site of albumin promoter (albumin D-box) binding protein | -3.98 | -14.31 | -5.61 |
| Depdc7 | Other | DEP domain containing 7 | -1.73 | -2.19 | -1.69 |
| Fam13c1 | Other | family with sequence similarity 13, member C | -1.57 | -1.63 | -2.01 |
| Fam46a | Other | family with sequence similarity 46, member A | -1.99 | -2.25 | -1.42 |
| Fam89a | Other | family with sequence similarity 89, member A | -4.34 | -4.50 | -4.33 |
| Fancc | Other | Fanconi anemia, complementation group C | -1.58 | -2.14 | -1.75 |
| Frmd4b | Other | FERM domain containing 4B | -1.64 | -2.08 | -1.05 |
| Gfi1 | Other | growth factor independent 1 transcription repressor | -1.07 | -1.93 | -2.13 |
| Gpr126 | Other | G protein-coupled receptor 126 | -3.46 | -5.59 | -3.86 |
| Gpr19 | Other | G protein-coupled receptor 19 | -1.34 | -1.86 | -2.04 |
| Gpr37l1 | Other | G protein-coupled receptor 37 like 1 | -2.26 | -2.34 | -2.46 |
| Gpr75 | Other | G protein-coupled receptor 75 | -2.52 | -2.43 | -1.81 |
| Gprasp2 | Other | G protein-coupled receptor associated sorting protein 2 | -2.30 | -3.43 | -2.77 |
| Gramd2 | Other | GRAM domain containing 2 | -2.74 | -4.07 | -2.85 |
| H19 | Other | H19, imprinted maternally expressed transcript (non-protein coding) | -3.87 | -4.12 | -2.52 |
| Haus7 | Other | HAUS augmin-like complex, subunit 7 | -1.99 | -2.15 | -1.87 |
| Hoxa9l | Other | homeobox A9-like | -2.24 | -2.52 | -1.35 |
| Hoxc6 | Other | homeobox C6 | -3.21 | -3.66 | -2.73 |
| Ica1 | Other | islet cell autoantigen 1, 69kDa | -3.32 | -4.00 | -2.84 |
| Ick | other | intestinal cell (MAK-like) kinase | -1.89 | -2.09 | -1.48 |
| Inadl | Other | InaD-like (Drosophila) | -4.00 | -5.31 | -4.87 |
| Jph1 | Other | junctophilin 1 | -4.55 | -4.55 | -2.94 |
| Klhl29 | Other | kelch-like 29 (Drosophila) | -4.55 | -5.38 | -4.37 |
| L3mbtl3 | Other | l(3)mbt-like 3 (Drosophila) | -1.41 | -2.08 | -1.63 |
| Lgr4 | Other | leucine-rich repeat-containing G protein-coupled receptor 4 | -1.80 | -4.01 | -2.31 |
| Lonrf1 | Other | LON peptidase N-terminal domain and ring finger 1 | -2.77 | -3.10 | -3.26 |
| Lrig3 | Other | leucine-rich repeats and immunoglobulin-like domains 3 | -2.98 | -3.24 | -2.03 |
| Lrrc1 | Other | leucine rich repeat containing 1 | -2.86 | -2.98 | -2.56 |
| Lypd6b | Other | LY6/PLAUR domain containing 6B | -5.35 | -5.60 | -5.49 |
| Mab21l2 | Other | mab-21-like 2 (C. elegans) | -2.88 | -3.38 | -3.15 |
| Maged2 | Other | melanoma antigen family D, 2 | -2.27 | -2.59 | -1.79 |
| Mef2c | Other | myocyte enhancer factor 2C | -1.65 | -2.55 | -1.86 |
| Mum1l1 | Other | melanoma associated antigen (mutated) 1-like 1 | -1.65 | -2.11 | -2.23 |
| Myh14 | Other | myosin, heavy chain 14 | -2.97 | -3.19 | -3.14 |
| Myo6 | Other | myosin VI | -2.47 | -3.02 | -1.92 |
| Ndn | Other | necdin homolog (mouse) | -1.81 | -2.43 | -2.69 |
| Ndrg2 | Other | NDRG family member 2 | -6.21 | -7.99 | -3.88 |
| Necab1 | Other | N-terminal EF-hand calcium binding protein 1 | -4.54 | -4.69 | -3.88 |
| Npal2 | Other | NIPA-like domain containing 2 | -2.80 | -2.81 | -1.64 |
| Npas3 | Other | neuronal PAS domain protein 3 | -2.21 | -2.80 | -2.66 |
| Nr1d1 | Other | plexin domain containing 1 | -6.32 | -15.25 | -6.66 |
| Nuak1 | other | NUAK family, SNF1-like kinase, 1 | -2.35 | -2.39 | -1.29 |
| Odz3 | Other | odz, odd Oz/ten-m homolog 3 (Drosophila) | -1.13 | -2.00 | -2.53 |
| Osbpl6 | Other | oxysterol binding protein-like 6 | -1.70 | -2.31 | -1.48 |
| Pabpc4l | Other | poly(A) binding protein, cytoplasmic 4-like | -1.96 | -2.16 | -1.61 |
| Phactr2 | Other | phosphatase and actin regulator 2 | -1.67 | -2.07 | -1.55 |
| Phospho1 | Other | phosphatase, orphan 1 | -6.53 | -6.63 | -5.20 |
| Plagl1 | Other | pleiomorphic adenoma gene-like 1 | -2.87 | -4.04 | -3.47 |
| Plekhh2 | Other | pleckstrin homology domain containing, family H (with MyTH4 domain) member 2 | -4.33 | -5.17 | -3.22 |
| Plxnb1 | Other | plexin B1 | -4.73 | -8.95 | -6.41 |
| Ppp1r3b | Other | protein phosphatase 1, regulatory (inhibitor) subunit 3B | -1.31 | -1.99 | -2.52 |
| Prdm6 | Other | PR domain containing 6 | -2.32 | -2.37 | -2.79 |
| Psd3 | Other | pleckstrin and Sec7 domain containing 3 | -2.19 | -2.52 | -1.62 |
| Pstpip2 | Other | proline-serine-threonine phosphatase interacting protein 2 | -3.72 | -5.48 | -5.63 |
| Ptcd3 | Other | Pentatricopeptide repeat domain 3 | -2.00 | -2.58 | -2.45 |
| Ptch1 | Other | patched homolog 1 (Drosophila) | -9.85 | -16.07 | -7.60 |
| R3hdml | Other | R3H domain containing-like | -2.84 | -3.93 | -4.02 |
| Rad9b | Other | RAD9 homolog B (S. pombe) | -1.64 | -2.01 | -1.70 |
| Ranbp3l | Other | RAN binding protein 3-like | -2.35 | -4.25 | -3.56 |
| Rcor2 | Other | REST corepressor 2 | -1.88 | -2.49 | -1.82 |
| Rcsd1 | Other | RCSD domain containing 1 | -1.73 | -2.39 | -2.45 |
| Rhbdl2 | Other | rhomboid, veinlet-like 2 (Drosophila) | -1.69 | -4.25 | -2.12 |
| Rimklb | Other | ribosomal modification protein rimK-like family member B | -3.21 | -3.42 | -4.12 |
| Scube1 | Other | signal peptide, CUB domain, EGF-like 1 | -13.67 | -17.84 | -12.15 |
| Scube3 | Other | signal peptide, CUB domain, EGF-like 3 | -5.05 | -5.79 | -4.64 |
| Sema6a | Other | sema domain, transmembrane domain (TM), and cytoplasmic domain, (semaphorin) 6A | -1.60 | -2.19 | -1.52 |
| Setbp1 | Other | SET binding protein 1 | -1.92 | -2.23 | -1.30 |
| Sgef | Other | Src homology 3 domain-containing guanine nucleotide exchange factor | -1.72 | -2.21 | -1.48 |
| Shisa2 | Other | shisa homolog 2 (Xenopus laevis) | -2.47 | -2.39 | -1.71 |
| Sim2 | Other | single-minded homolog 2 (Drosophila) | -3.64 | -4.25 | -2.86 |
| Smo | Other | smoothened homolog (Drosophila) | -3.70 | -3.93 | -2.96 |
| Sobpl | Other | sine oculis binding protein homolog (Drosophila) | -2.76 | -3.69 | -2.36 |
| Sorbs1 | Other | sorbin and SH3 domain containing 1 | -3.56 | -5.31 | -3.39 |
| Spata18 | Other | spermatogenesis associated 18 homolog (rat) | -1.56 | -2.45 | -1.93 |
| Srrp | Other | serine-arginine repressor protein (35 kDa) | -1.93 | -2.06 | -1.49 |
| Sspn | Other | sarcospan (Kras oncogene-associated gene) | -2.77 | -3.16 | -1.29 |
| Stxbp6 | Other | syntaxin binding protein 6 (amisyn) | -2.69 | -2.93 | -1.97 |
| Susd5 | Other | sushi domain containing 5 | -5.25 | -5.31 | -3.34 |
| Syde2 | Other | synapse defective 1, Rho GTPase, homolog 2 (C. elegans) | -1.64 | -2.20 | -1.72 |
| Tcea3 | Other | transcription elongation factor A (SII), 3 | -3.36 | -3.64 | -2.03 |
| Tet1 | Other | tet oncogene 1 | -1.94 | -2.11 | -1.54 |
| Thnsl2 | Other | threonine synthase-like 2 (S. cerevisiae) | -1.99 | -2.55 | -1.93 |
| Tlcd1 | Other | TLC domain containing 1 | -1.94 | -2.37 | -1.92 |
| Tmem56 | Other | transmembrane protein 56 | -2.08 | -2.38 | -2.38 |
| Tmem98 | Other | transmembrane protein 98 | -2.08 | -2.25 | -1.97 |
| Tmod2 | Other | tropomodulin 2 (neuronal) | -1.96 | -2.60 | -1.19 |
| Tmtc1 | Other | transmembrane and tetratricopeptide repeat containing 1 | -4.05 | -4.32 | -2.44 |
| Tnnt1 | Other | troponin T type 1 (skeletal, slow) | -3.05 | -3.11 | -2.77 |
| Tpst1 | Other | RAB guanine nucleotide exchange factor (GEF) 1 | -2.10 | -2.03 | -1.50 |
| Trim72 | Other | tripartite motif-containing 72 | -1.92 | -2.01 | -1.92 |
| Yipf2 | Other | Yip1 domain family, member 2 | -1.82 | -2.35 | -1.80 |
| Zbtb20 | Other | zinc finger and BTB domain containing 20 | -4.26 | -4.56 | -2.21 |
| Zcchc5 | Other | zinc finger, CCHC domain containing 5 | -7.38 | -7.41 | -7.12 |
| Zfp157 | Other | zinc finger protein 157 | -1.41 | -2.15 | -1.33 |
| Zfp385c | Other | zinc finger protein 385C | -7.64 | -8.04 | -8.02 |
| Zfp395 | Other | zinc finger protein 395 | -2.00 | -2.25 | -1.81 |
| Zfp40 | Other | zinc finger protein 40 | -1.69 | -2.12 | -1.20 |
| Zfp61 | Other | zinc finger protein 61 | -1.87 | -2.15 | -2.10 |
| Zfp748 | Other | zinc finger protein 748 | -1.27 | -2.18 | -1.41 |
| Zfp9 | Other | zinc finger protein 9 | -1.97 | -2.13 | -1.63 |
| Zfpm2 | Other | zinc finger protein, multitype 2 | -2.65 | -2.66 | -1.82 |
| Zhx3 | Other | zinc fingers and homeoboxes 3 | -2.24 | -2.30 | -1.40 |
| Zim1 | Other | zinc finger, imprinted 1 | -6.79 | -9.67 | -9.60 |
| Znf124 | Other | zinc finger protein 124 | -1.89 | -2.07 | -1.86 |
| Znf23 | Other | zinc finger protein 23 (KOX 16) | -2.36 | -2.79 | -1.92 |
| Abca5 | Transporter | ATP-binding cassette, sub-family A (ABC1), member 5 | -2.09 | -2.64 | -1.74 |
| Atp1b1 | Transporter | ATPase, Na+/K+ transporting, beta 1 polypeptide | -1.22 | -2.19 | -1.56 |
| Atp8b1 | Transporter | ATPase, class I, type 8B, member 1 | -6.37 | -6.88 | -4.57 |
| Cacna1d | Transporter | calcium channel, voltage-dependent, L type, alpha 1D subunit | -2.43 | -3.05 | -2.94 |
| Clcn4-2 | Transporter | chloride channel 4 | -3.15 | -3.74 | -2.57 |
| Disp1 | Transporter | dispatched homolog 1 (Drosophila) | -4.01 | -4.33 | -3.95 |
| Flvcr2 | Transporter | feline leukemia virus subgroup C cellular receptor family, member 2 | -2.33 | -4.15 | -2.94 |
| Fxyd3 | Transporter | FXYD domain containing ion transport regulator 3 | -3.71 | -4.48 | -4.23 |
| Gria2 | Transporter | glutamate receptor, ionotropic, AMPA 2 | -1.12 | -2.09 | -1.95 |
| Kcnk1 | Transporter | potassium channel, subfamily K, member 1 | -2.07 | -2.84 | -2.44 |
| Kcnma1 | Transporter | potassium large conductance calcium-activated channel, subfamily M, alpha member 1 | -3.45 | -4.34 | -2.03 |
| Kcnn2 | Transporter | potassium intermediate/small conductance calcium-activated channel, subfamily N, member 2 | -2.14 | -2.74 | -2.56 |
| Kcnq5l | Transporter | potassium voltage-gated channel, KQT-like subfamily, member 5 | -2.59 | -2.67 | -2.64 |
| Kcnt2 | Transporter | potassium channel, subfamily T, member 2 | -2.03 | -3.09 | -1.96 |
| Kctd4 | Transporter | potassium channel tetramerisation domain containing 4 | -3.71 | -5.53 | -4.93 |
| Mug1 | Transporter | murinoglobulin 1 | -30.10 | -35.85 | -39.17 |
| Mug2 | Transporter | C3 and PZP-like, alpha-2-macroglobulin domain containing 8 | -10.63 | -11.88 | -10.88 |
| Rab11fip4 | Transporter | RAB11 family interacting protein 4 (class II) | -8.36 | -8.82 | -6.05 |
| Rbp4 | Transporter | retinol binding protein 4, plasma | -4.70 | -6.05 | -4.34 |
| Scarb2 | Transporter | scavenger receptor class B, member 2 | -2.21 | -2.21 | -1.30 |
| Scn1a | Transporter | sodium channel, voltage-gated, type I, alpha subunit | -2.21 | -2.13 | -1.17 |
| Slc13a5 | Transporter | solute carrier family 13 (sodium-dependent citrate transporter), member 5 | -2.02 | -2.72 | -1.77 |
| Slc16a2 | Transporter | solute carrier family 16, member 2 (monocarboxylic acid transporter 8) | -2.03 | -2.23 | -1.37 |
| Slc22a23 | Transporter | solute carrier family 22, member 23 | -1.88 | -2.89 | -3.07 |
| Slc25a38 | Transporter | solute carrier family 25, member 38 | -1.29 | -1.63 | -2.02 |
| Slc26a2 | Transporter | solute carrier family 26 (sulfate transporter), member 2 | -1.98 | -2.76 | -2.55 |
| Slc27a6 | Transporter | solute carrier family 27 (fatty acid transporter), member 6 | -6.81 | -7.41 | -6.33 |
| Slc35f2 | Transporter | solute carrier family 35, member F2 | -5.39 | -8.08 | -5.82 |
| Slc36a2 | Transporter | solute carrier family 36 (proton/amino acid symporter), member 2 | -1.30 | -2.71 | -2.13 |
| Slc38a3 | Transporter | solute carrier family 38, member 3 | -13.33 | -18.26 | -12.55 |
| Slc39a8 | Transporter | solute carrier family 39 (zinc transporter), member 8 | -1.98 | -2.35 | -2.00 |
| Slc44a2 | Transporter | solute carrier family 44, member 2 | -1.93 | -2.11 | -1.64 |
| Slc8a3 | Transporter | solute carrier family 8 (sodium/calcium exchanger), member 3 | -3.69 | -7.32 | -3.26 |
| Slco2b1 | Transporter | solute carrier organic anion transporter family, member 2B1 | -1.15 | -2.11 | -1.31 |
| Snx24 | Transporter | sorting nexin 24 | -1.86 | -2.47 | -1.68 |
| Sorl1 | Transporter | sortilin-related receptor, L(DLR class) A repeats-containing | -1.54 | -1.65 | -2.39 |
| Steap1 | Transporter | six transmembrane epithelial antigen of the prostate 1 | -1.91 | -2.26 | -2.64 |
| Syt8 | Transporter | synaptotagmin VIII | -2.02 | -2.13 | -2.21 |
| Tc2n | Transporter | tandem C2 domains, nuclear | -3.03 | -4.26 | -3.28 |
| Tmco3 | Transporter | transmembrane and coiled-coil domains 3 | -2.80 | -2.89 | -3.01 |
| Vldlr | Transporter | very low density lipoprotein receptor | -1.98 | -2.50 | -1.40 |
