# Supplementary material for: Sequential Alterations in Catabolic and Anabolic Gene Expression Parallel Pathological Changes during Progression of Monoiodoacetate-Induced Arthritis
Source: PLoS One. 2011 Sep 13;6(9):e24320. doi: 10.1371/journal.pone.0024320 (PMC3172226; doi:10.1371/journal.pone.0024320)
Supplement: Table S5 — Changes in the expression of genes in Cluster III. Please see Table S1 for group description. (DOC) [file pone.0024320.s006.doc]

**Table S5**. Changes in the expression of genes in *Cluster III*. Please see Table S1 for group description.

| Gene | Group | Description | MIA5 | MIA9 | MIA21 |
| --- | --- | --- | --- | --- | --- |
| Ahr | CD | aryl hydrocarbon receptor | 2.16 | 2.26 | 2.66 |
| Bcl2l11 | CD | BCL2-like 11 (apoptosis facilitator) | 2.25 | 1.82 | 2.26 |
| Casp3 | CD | caspase 3, apoptosis-related cysteine peptidase | 1.90 | 1.69 | 2.03 |
| Ccnd1 | CD | cyclin D1 | 2.28 | 2.22 | 2.72 |
| Cdkn1a | CD | cyclin-dependent kinase inhibitor 1A (p21, Cip1) | 1.10 | 1.95 | 2.56 |
| Cux1 | CD | cut-like homeobox 1 | 1.87 | 2.02 | 1.95 |
| Frk | CD | fyn-related kinase | 1.94 | 1.88 | 2.61 |
| Klf10 | CD | Kruppel-like factor 10 | 1.36 | 2.56 | 2.42 |
| Klf4 | CD | Kruppel-like factor 4 (gut) | 1.02 | 1.84 | 2.26 |
| Klf7 | CD | Kruppel-like factor 7 (ubiquitous) | 1.50 | 2.00 | 2.26 |
| Mfhas1 | CD | malignant fibrous histiocytoma amplified sequence 1 | 2.12 | 2.07 | 2.22 |
| Notch3 | CD | Notch homolog 3 (Drosophila) | 1.53 | 2.10 | 2.76 |
| Pawr | CD | PRKC, apoptosis, WT1, regulator | 1.71 | 1.98 | 2.44 |
| Ptpn13 | CD | protein tyrosine phosphatase, non-receptor type 13 (APO-1/CD95 (Fas)-associated phosphatase) | 2.12 | 2.24 | 2.31 |
| Repin1 | CD | replication initiator 1 | 1.47 | 2.54 | 2.42 |
| Col12a1 | ECM | collagen, type XII, alpha 1 | 2.10 | 3.11 | 3.42 |
| Col18a1 | ECM | collagen, type XVIII, alpha 1 | 2.71 | 3.92 | 5.66 |
| Col3a1 | ECM | collagen, type III, alpha 1 | 2.02 | 3.19 | 3.88 |
| Col4a1 | ECM | collagen, type IV, alpha 1 | 1.80 | 3.03 | 4.33 |
| Col4a2 | ECM | collagen, type IV, alpha 2 | 1.39 | 2.12 | 3.13 |
| Col5a1 | ECM | collagen, type V, alpha 1 | 1.10 | 1.73 | 2.11 |
| Col6a3 | ECM | collagen, type VI, alpha 3 | 1.30 | 2.42 | 2.71 |
| Dpt | ECM | dermatopontin | 1.80 | 3.11 | 5.41 |
| Gal | ECM | galanin prepropeptide | 5.17 | 7.74 | 13.93 |
| Hmcn1 | ECM | hemicentin 1 | 4.70 | 5.73 | 8.77 |
| Lad1 | ECM | ladinin 1 | 1.52 | 2.76 | 2.51 |
| Lama2 | ECM | laminin, alpha 2 | 1.75 | 1.89 | 4.11 |
| Lama5 | ECM | laminin, alpha 5 | 1.20 | 2.00 | 2.62 |
| Lamb1 | ECM | laminin, beta 1 | 4.94 | 5.73 | 6.77 |
| Lamc1 | ECM | laminin, gamma 1 (formerly LAMB2) | 1.85 | 2.40 | 2.78 |
| Lgals1 | ECM | lectin, galactoside-binding, soluble, 1 | 3.62 | 4.10 | 4.81 |
| Lum | ECM | lumican | 4.09 | 5.03 | 5.90 |
| Postn | ECM | periostin, osteoblast specific factor | 5.88 | 5.05 | 7.23 |
| Tmod3 | ECM | tropomodulin 3 (ubiquitous) | 1.77 | 2.13 | 2.62 |
| Tnn | ECM | tenascin N | 15.51 | 18.80 | 20.85 |
| Adam12 | ECM2 | ADAM metallopeptidase domain 12 | 2.07 | 2.05 | 2.20 |
| Adam23 | ECM2 | ADAM metallopeptidase domain 23 | 3.97 | 3.56 | 5.50 |
| Adam9 | ECM2 | ADAM metallopeptidase domain 9 (meltrin gamma) | 2.09 | 2.18 | 2.59 |
| Adamts2 | ECM2 | ADAM metallopeptidase with thrombospondin type 1 motif, 2 | 1.28 | 1.62 | 2.37 |
| Adamtsl5 | ECM2 | ADAMTS-like 5 | 1.38 | 2.22 | 1.97 |
| Cdh11 | ECM2 | cadherin 11, type 2, OB-cadherin (osteoblast) | 1.91 | 1.64 | 2.33 |
| Cpa6 | ECM2 | carboxypeptidase A6 | 1.73 | 1.85 | 2.08 |
| Cthrc1 | ECM2 | collagen triple helix repeat containing 1 | 1.38 | 2.10 | 1.82 |
| Ctsb | ECM2 | cathepsin B | 1.34 | 1.91 | 2.05 |
| Ctsd | ECM2 | cathepsin D | 1.61 | 2.53 | 2.25 |
| Ctsk | ECM2 | cathepsin K | 2.13 | 2.70 | 2.98 |
| Dcbld2 | ECM2 | discoidin, CUB and LCCL domain containing 2 | 1.62 | 1.95 | 2.29 |
| Ecm1 | ECM2 | extracellular matrix protein 1 | 1.26 | 2.34 | 3.69 |
| Fndc1 | ECM2 | fibronectin type III domain containing 1 | 1.97 | 2.75 | 6.75 |
| Mme | ECM2 | membrane metallo-endopeptidase | 1.49 | 1.46 | 2.02 |
| Mmp11 | ECM2 | matrix metallopeptidase 11 (stromelysin 3) | 1.11 | 1.47 | 2.39 |
| Mmp14 | ECM2 | matrix metallopeptidase 14 (membrane-inserted) | 1.94 | 3.29 | 3.01 |
| Mmp2 | ECM2 | matrix metallopeptidase 2 (gelatinase A, 72kDa gelatinase, 72kDa type IV collagenase) | 1.01 | 1.99 | 2.84 |
| Pcdh1 | ECM2 | protocadherin 1 | 1.60 | 1.51 | 2.16 |
| Prss23 | ECM2 | protease, serine, 23 | 1.42 | 2.49 | 2.78 |
| Scpep1 | ECM2 | serine carboxypeptidase 1 | 1.50 | 2.22 | 3.16 |
| Serpine1 | ECM2 | serpin peptidase inhibitor, clade E (nexin, plasminogen activator inhibitor type 1), member 1 | 3.27 | 3.88 | 4.81 |
| Thbs2 | ECM2 | thrombospondin 2 | 1.81 | 2.22 | 3.42 |
| Thbs4 | ECM2 | thrombospondin 4 | 1.97 | 1.50 | 2.71 |
| Timp2 | ECM2 | TIMP metallopeptidase inhibitor 2 | 1.38 | 2.19 | 3.07 |
| Acvrl1 | GF | activin A receptor type II-like 1 | 1.59 | 1.39 | 2.03 |
| Egfr | GF | epidermal growth factor receptor (erythroblastic leukemia viral (v-erb-b) oncogene homolog, avian) | 1.29 | 1.17 | 2.05 |
| Ltbp2 | GF | latent transforming growth factor beta binding protein 2 | 2.70 | 2.30 | 3.60 |
| Ogn | GF | osteoglycin | 1.22 | 1.19 | 2.06 |
| Osmr | GF | oncostatin M receptor | 1.85 | 2.41 | 2.45 |
| Ostm1 | GF | osteopetrosis associated transmembrane protein 1 | 2.41 | 3.01 | 2.79 |
| Pdgfc | GF | platelet derived growth factor C | 1.04 | 2.22 | 2.15 |
| Pdgfrb | GF | platelet-derived growth factor receptor, beta polypeptide | 1.18 | 1.77 | 2.46 |
| Tgfb2 | GF | transforming growth factor, beta 2 | 1.26 | 2.67 | 2.63 |
| Tgfbr2 | GF | transforming growth factor, beta receptor II (70/80kDa) | 1.48 | 2.13 | 1.88 |
| Bmp2k | GF2 | BMP-2 inducible kinase | 2.03 | 2.04 | 2.07 |
| Cdh13 | GF2 | cadherin 13, H-cadherin (heart) | 1.46 | 4.63 | 8.59 |
| Efemp1 | GF2 | EGF-containing fibulin-like extracellular matrix protein 1 | 1.09 | 5.06 | 7.45 |
| Esm1 | GF2 | endothelial cell-specific molecule 1 | 2.18 | 2.49 | 2.40 |
| Megf6 | GF2 | multiple EGF-like-domains 6 | 2.41 | 3.11 | 4.84 |
| Wisp2 | GF2 | WNT1 inducible signaling pathway protein 2 | 2.65 | 5.71 | 6.08 |
| Clec2dl1 | Inf | C-type lectin domain family 2, member A | 4.18 | 5.45 | 9.55 |
| Clec4d | Inf | C-type lectin domain family 4, member D | 5.38 | 4.81 | 5.20 |
| Cxcl13 | Inf | chemokine (C-X-C motif) ligand 13 | 1.63 | 2.10 | 2.98 |
| Il10rb | Inf | interleukin 10 receptor, beta | 2.14 | 2.40 | 2.81 |
| Il15 | Inf | interleukin 15 | 1.26 | 1.48 | 2.01 |
| Lbp | Inf | lipopolysaccharide binding protein | 1.66 | 3.65 | 4.45 |
| Tlr4 | Inf | toll-like receptor 4 | 2.14 | 1.83 | 2.85 |
| Tnfaip2 | Inf | tumor necrosis factor, alpha-induced protein 2 | 1.92 | 2.06 | 1.96 |
| Alox5 | Inf2 | arachidonate 5-lipoxygenase | 1.82 | 1.61 | 2.86 |
| Arhgap28 | Inf2 | Rho GTPase activating protein 28 | 1.85 | 2.37 | 2.20 |
| C1qb | Inf2 | complement component 1, q subcomponent, B chain | 1.64 | 1.75 | 2.27 |
| C1qtnf3 | Inf2 | C1q and tumor necrosis factor related protein 3 | 5.05 | 5.27 | 12.10 |
| C1qtnf5 | Inf2 | C1q and tumor necrosis factor related protein 5 | 1.73 | 1.77 | 2.82 |
| C1r | Inf2 | complement component 1, r subcomponent | 1.41 | 1.99 | 3.58 |
| C1s | Inf2 | complement component 1, s subcomponent | 1.64 | 1.98 | 5.18 |
| Cav1 | Inf2 | caveolin 1, caveolae protein, 22kDa | 2.16 | 2.20 | 2.69 |
| Cd14 | Inf2 | CD14 molecule | 3.26 | 6.02 | 7.15 |
| Cd34 | Inf2 | CD34 molecule | 1.32 | 1.35 | 2.24 |
| Cd4 | Inf2 | CD4 molecule | 2.90 | 2.97 | 3.52 |
| Cd93 | Inf2 | CD93 molecule | 2.30 | 2.59 | 3.01 |
| Cdc42bpb | Inf2 | CDC42 binding protein kinase beta (DMPK-like) | 1.46 | 2.00 | 1.94 |
| Clec4e | Inf2 | C-type lectin domain family 4, member E | 3.10 | 3.21 | 3.86 |
| Crlf1 | Inf2 | cytokine receptor-like factor 1 | 1.81 | 4.19 | 4.49 |
| Dagla | Inf2 | diacylglycerol lipase, alpha | 2.05 | 2.89 | 2.86 |
| F3 | Inf2 | coagulation factor III (thromboplastin, tissue factor) | 2.05 | 2.56 | 3.14 |
| Fcer1g | Inf2 | Fc fragment of IgE, high affinity I, receptor for; gamma polypeptide | 2.56 | 2.47 | 2.51 |
| Fert2 | Inf2 | fer (fps/fes related) tyrosine kinase | 2.07 | 1.53 | 1.93 |
| Flt1 | Inf2 | fms-related tyrosine kinase 1 (vascular endothelial growth factor/vascular permeability factor receptor) | 2.04 | 2.52 | 2.67 |
| Gas7 | Inf2 | growth arrest-specific 7 | 1.79 | 1.68 | 2.10 |
| Hs1bp3 | Inf2 | HCLS1 binding protein 3 | 1.92 | 2.14 | 2.03 |
| Hspb1 | Inf2 | heat shock 27kDa protein 1 | 1.17 | 2.21 | 2.95 |
| Hsph1 | Inf2 | heat shock 105kDa/110kDa protein 1 | 2.01 | 2.21 | 2.19 |
| Ifitm3 | Inf2 | interferon induced transmembrane protein 3 (1-8U) | 1.89 | 2.26 | 2.22 |
| Igsf9b | Inf2 | immunoglobulin superfamily, member 9B | 1.25 | 1.52 | 2.60 |
| Itga11 | Inf2 | integrin, alpha 11 | 1.34 | 2.10 | 2.57 |
| Itga5 | Inf2 | integrin, alpha 5 (fibronectin receptor, alpha polypeptide) | 1.43 | 2.25 | 1.93 |
| Itgb1 | Inf2 | integrin, beta 1 (fibronectin receptor, beta polypeptide, antigen CD29 includes MDF2, MSK12) | 1.75 | 1.84 | 2.14 |
| Itgb4 | Inf2 | integrin, beta 4 | 1.10 | 1.41 | 2.15 |
| Lilrb4 | Inf2 | leukocyte immunoglobulin-like receptor, subfamily B, member 4 | 7.33 | 6.00 | 6.98 |
| Map3k6 | Inf2 | mitogen-activated protein kinase kinase kinase 6 | 1.88 | 2.02 | 2.09 |
| Mertk | Inf2 | c-mer proto-oncogene tyrosine kinase | 1.60 | 1.42 | 2.10 |
| Mill2 | Inf2 | MHC class I polypeptide-related sequence B | 1.43 | 1.59 | 2.28 |
| Nfkbia | Inf2 | nuclear factor of kappa light polypeptide gene enhancer in B-cells inhibitor, alpha | 1.42 | 2.45 | 2.17 |
| Pi4k2a | Inf2 | phosphatidylinositol 4-kinase type 2 alpha | 1.38 | 2.22 | 1.95 |
| Pik3r5 | Inf2 | phosphoinositide-3-kinase, regulatory subunit 5 | 2.43 | 3.62 | 3.51 |
| Pld2 | Inf2 | phospholipase D2 | 1.72 | 2.32 | 2.49 |
| Prkx | Inf2 | protein kinase, X-linked | 1.97 | 2.37 | 2.36 |
| Ptges | Inf2 | prostaglandin E synthase | 1.40 | 2.34 | 2.60 |
| Ptgfrn | Inf2 | prostaglandin F2 receptor negative regulator | 2.59 | 2.63 | 3.32 |
| Rgs4 | Inf2 | regulator of G-protein signaling 4 | 1.30 | 2.40 | 3.15 |
| Rin2 | Inf2 | Ras and Rab interactor 2 | 1.30 | 1.70 | 2.82 |
| Sema3c | Inf2 | sema domain, immunoglobulin domain (Ig), short basic domain, secreted, (semaphorin) 3C | 1.80 | 1.67 | 2.53 |
| Sema3f | Inf2 | sema domain, immunoglobulin domain (Ig), short basic domain, secreted, (semaphorin) 3F | 1.85 | 2.31 | 2.55 |
| Socs3 | Inf2 | suppressor of cytokine signaling 3 | 1.69 | 2.09 | 2.47 |
| Tec | Inf2 | tec protein tyrosine kinase | 2.44 | 2.65 | 2.59 |
| Tek | Inf2 | TEK tyrosine kinase, endothelial | 1.62 | 1.50 | 2.08 |
| Thy1 | Inf2 | Thy-1 cell surface antigen | 3.23 | 5.10 | 5.07 |
| Twist1 | Inf2 | twist homolog 1 (Drosophila) | 1.50 | 1.95 | 2.23 |
| Vcam1 | Inf2 | vascular cell adhesion molecule 1 | 2.69 | 1.82 | 2.61 |
| A3galt2 | Meta | alpha 1,3-galactosyltransferase 2 | 2.89 | 3.32 | 3.83 |
| Actn1 | Meta | actinin, alpha 1 | 2.03 | 2.21 | 2.06 |
| Actr2 | Meta | ARP2 actin-related protein 2 homolog (yeast) | 2.11 | 2.25 | 2.11 |
| Actr3 | Meta | ARP3 actin-related protein 3 homolog (yeast) | 2.06 | 2.42 | 2.28 |
| Adcy3 | Meta | adenylate cyclase 3 | 1.85 | 2.60 | 2.57 |
| Adcy4 | Meta | adenylate cyclase 4 | 1.74 | 1.93 | 2.10 |
| Agpat4 | Meta | 1-acylglycerol-3-phosphate O-acyltransferase 4 (lysophosphatidic acid acyltransferase, delta) | 1.42 | 2.20 | 2.08 |
| Ahnak | Meta | AHNAK nucleoprotein | 1.34 | 1.91 | 2.32 |
| Ak3 | Meta | adenylate kinase 3 | 1.37 | 1.62 | 2.00 |
| Akap13 | Meta | A kinase (PRKA) anchor protein 13 | 1.93 | 2.07 | 2.45 |
| Akr1b8 | Meta | aldo-keto reductase family 1, member B8 | 3.00 | 5.33 | 5.49 |
| Anxa1 | Meta | annexin A1 | 1.75 | 2.66 | 2.90 |
| Anxa5 | Meta | annexin A5 | 1.36 | 2.03 | 2.04 |
| Arl4c | Meta | ADP-ribosylation factor-like 4C | 5.02 | 5.83 | 5.65 |
| Arl8b | Meta | ADP-ribosylation factor-like 8B | 1.49 | 2.01 | 1.99 |
| Asah1 | Meta | N-acylsphingosine amidohydrolase (acid ceramidase) 1 | 1.47 | 2.02 | 2.42 |
| Atrnl1 | Meta | attractin-like 1 | 1.97 | 2.28 | 2.77 |
| B2m | Meta | beta-2-microglobulin | 2.00 | 2.29 | 2.43 |
| B3galnt1 | Meta | beta-1,3-N-acetylgalactosaminyltransferase 1 (globoside blood group) | 1.61 | 1.88 | 2.25 |
| B4galt1 | Meta | UDP-Gal:betaGlcNAc beta 1,4- galactosyltransferase, polypeptide 1 | 2.45 | 2.57 | 2.44 |
| B4galt5 | Meta | UDP-Gal:betaGlcNAc beta 1,4- galactosyltransferase, polypeptide 5 | 2.39 | 3.38 | 3.20 |
| Calcrl | Meta | calcitonin receptor-like | 1.91 | 1.56 | 2.60 |
| Calm1 | Meta | calmodulin 1 (phosphorylase kinase, delta) | 1.58 | 1.82 | 2.13 |
| Cap1 | Meta | CAP, adenylate cyclase-associated protein 1 (yeast) | 2.00 | 2.26 | 2.15 |
| Cbr3 | Meta | carbonyl reductase 3 | 1.57 | 1.49 | 2.00 |
| Cfl1 | Meta | cofilin 1 (non-muscle) | 1.79 | 2.25 | 2.19 |
| Ch25h | Meta | cholesterol 25-hydroxylase | 2.16 | 2.53 | 2.66 |
| Coro1c | Meta | coronin, actin binding protein, 1C | 2.49 | 2.92 | 3.05 |
| Cpeb2 | Meta | cytoplasmic polyadenylation element binding protein 2 | 1.85 | 1.98 | 2.12 |
| Crabp2 | Meta | cellular retinoic acid binding protein 2 | 5.89 | 7.15 | 8.53 |
| Cry1 | Meta | cryptochrome 1 (photolyase-like) | 1.54 | 2.64 | 2.82 |
| Cyyr1 | Meta | cysteine/tyrosine-rich 1 | 1.71 | 1.82 | 2.30 |
| Depdc2 | Meta | phosphatidylinositol-3,4,5-trisphosphate-dependent Rac exchange factor 2 | 2.12 | 1.82 | 2.29 |
| Dpysl2 | Meta | dihydropyrimidinase-like 2 | 1.41 | 1.91 | 2.44 |
| Dpysl3 | Meta | dihydropyrimidinase-like 3 | 1.82 | 1.70 | 2.90 |
| Ehd4 | Meta | EH-domain containing 4 | 3.48 | 3.47 | 3.52 |
| Eltd1 | Meta | EGF, latrophilin and seven transmembrane domain containing 1 | 1.87 | 2.07 | 2.96 |
| Emcn | Meta | endomucin | 1.72 | 1.62 | 2.44 |
| Enpp3 | Meta | ectonucleotide pyrophosphatase/phosphodiesterase 3 | 3.91 | 5.19 | 5.86 |
| Entpd1 | Meta | ectonucleoside triphosphate diphosphohydrolase 1 | 2.31 | 1.86 | 3.02 |
| Epb4.1l3 | Meta | erythrocyte membrane protein band 4.1-like 3 | 2.30 | 1.68 | 2.28 |
| Epdr1 | Meta | ependymin related protein 1 (zebrafish) | 1.74 | 1.98 | 2.01 |
| Fnbp1l | Meta | formin binding protein 1-like | 2.06 | 2.21 | 2.36 |
| Galm | Meta | galactose mutarotase (aldose 1-epimerase) | 1.86 | 3.37 | 3.07 |
| Galnt3 | Meta | UDP-N-acetyl-alpha-D-galactosamine:polypeptide N-acetylgalactosaminyltransferase 3 (GalNAc-T3) | 1.85 | 1.98 | 2.28 |
| Galntl1 | Meta | UDP-N-acetyl-alpha-D-galactosamine:polypeptide N-acetylgalactosaminyltransferase-like 1 | 2.09 | 1.46 | 2.24 |
| Gcgr | Meta | glucagon receptor | 1.35 | 2.07 | 2.46 |
| Gdap2 | Meta | ganglioside induced differentiation associated protein 2 | 1.98 | 1.82 | 2.18 |
| Gfra2 | Meta | GDNF family receptor alpha 2 | 1.05 | 1.47 | 2.04 |
| Glb1 | Meta | galactosidase, beta 1 | 1.21 | 2.29 | 2.61 |
| Glb1l | Meta | galactosidase, beta 1-like | 1.17 | 1.50 | 2.46 |
| Glt8d4 | Meta | glycosyltransferase 8 domain containing 4 | 1.63 | 1.59 | 2.08 |
| Glud1 | Meta | glutamate dehydrogenase 1 | 1.60 | 1.84 | 2.38 |
| Gna13 | Meta | guanine nucleotide binding protein (G protein), alpha 13 | 1.61 | 2.14 | 2.14 |
| Ids | Meta | iduronate 2-sulfatase | 1.25 | 1.39 | 2.03 |
| Ier5 | Meta | immediate early response 5 | 1.76 | 2.20 | 2.09 |
| Kctd11 | Meta | potassium channel tetramerisation domain containing 11 | 1.70 | 3.02 | 2.90 |
| Kdsr | Meta | 3-ketodihydrosphingosine reductase | 1.58 | 1.71 | 2.38 |
| Lama4 | Meta | laminin, alpha 4 | 2.00 | 2.08 | 2.26 |
| Large | Meta | like-glycosyltransferase | 2.26 | 2.40 | 2.89 |
| Lima1 | Meta | LIM domain and actin binding 1 | 1.84 | 1.51 | 2.13 |
| Lipa | Meta | lipase A, lysosomal acid, cholesterol esterase | 2.16 | 1.98 | 2.09 |
| Lnx1 | Meta | ligand of numb-protein X 1 | 1.60 | 2.05 | 2.43 |
| Lonrf3 | Meta | LON peptidase N-terminal domain and ring finger 3 | 1.53 | 1.63 | 2.02 |
| Lphn2 | Meta | latrophilin 2 | 2.28 | 2.11 | 3.13 |
| Lpl | Meta | lipoprotein lipase | 1.36 | 1.58 | 2.91 |
| Mfap3l | Meta | microfibrillar-associated protein 3-like | 1.96 | 2.91 | 2.74 |
| Mical2 | Meta | microtubule associated monoxygenase, calponin and LIM domain containing 2 | 2.44 | 2.70 | 3.05 |
| Msn | Meta | moesin | 2.21 | 2.94 | 2.98 |
| Myh9 | Meta | myosin, heavy chain 9, non-muscle | 2.32 | 2.55 | 2.45 |
| Myo7a | Meta | myosin VIIA | 1.88 | 1.73 | 2.02 |
| Naaladl2 | Meta | N-acetylated alpha-linked acidic dipeptidase-like 2 | 1.30 | 1.25 | 2.35 |
| Nav3 | Meta | neuron navigator 3 | 3.84 | 3.44 | 4.20 |
| Nbl1 | Meta | neuroblastoma, suppression of tumorigenicity 1 | 1.01 | 1.44 | 3.69 |
| Nnmt | Meta | nicotinamide N-methyltransferase | 1.40 | 1.47 | 2.15 |
| Nos3 | Meta | nitric oxide synthase 3 (endothelial cell) | 1.67 | 1.88 | 2.08 |
| Nox4 | Meta | NADPH oxidase 4 | 2.20 | 1.91 | 2.73 |
| Nr2f2 | Meta | nuclear receptor subfamily 2, group F, member 2 | 1.96 | 1.43 | 2.00 |
| Osbp2 | Meta | oxysterol binding protein 2 | 1.05 | 1.37 | 2.11 |
| P4ha3 | Meta | prolyl 4-hydroxylase, alpha polypeptide III | 7.06 | 6.15 | 7.14 |
| Paqr7 | Meta | progestin and adipoQ receptor family member VII | 1.93 | 1.92 | 2.00 |
| Pde8a | Meta | phosphodiesterase 8A | 2.57 | 3.33 | 3.78 |
| Pepd | Meta | peptidase D | 1.58 | 1.81 | 2.11 |
| Pgm2l1 | Meta | phosphoglucomutase 2-like 1 | 2.94 | 2.23 | 2.84 |
| Pltp | Meta | phospholipid transfer protein | 1.85 | 1.11 | 2.21 |
| Podxl | Meta | podocalyxin-like | 2.18 | 3.05 | 3.95 |
| Prkag2 | Meta | protein kinase, AMP-activated, gamma 2 non-catalytic subunit | 1.87 | 2.19 | 2.18 |
| Prrx1 | Meta | paired related homeobox 1 | 1.78 | 1.73 | 2.62 |
| Ptpn12 | Meta | protein tyrosine phosphatase, non-receptor type 12 | 1.76 | 2.15 | 2.15 |
| Ptprb | Meta | protein tyrosine phosphatase, receptor type, B | 2.20 | 2.50 | 2.92 |
| Pxk | Meta | PX domain containing serine/threonine kinase | 1.75 | 1.75 | 2.06 |
| Rab31 | Meta | RAB31, member RAS oncogene family | 1.63 | 2.12 | 2.91 |
| Rap1b | Meta | RAP1B, member of RAS oncogene family | 1.80 | 1.69 | 2.06 |
| Rap2b | Meta | RAP2B, member of RAS oncogene family | 2.02 | 1.92 | 2.05 |
| Rasal2 | Meta | RAS protein activator like 2 | 1.95 | 2.53 | 2.67 |
| Rasgrp3 | Meta | RAS guanyl releasing protein 3 (calcium and DAG-regulated) | 2.33 | 2.07 | 2.43 |
| Rbpj | Meta | recombination signal binding protein for immunoglobulin kappa J region | 1.30 | 1.35 | 2.07 |
| Reln | Meta | reelin | 3.36 | 3.54 | 3.55 |
| Renbp | Meta | renin binding protein | 2.00 | 2.12 | 2.43 |
| Rnase4 | Meta | ribonuclease, RNase A family, 4 | 1.12 | 1.58 | 2.61 |
| Rnd3 | Meta | Rho family GTPase 3 | 1.95 | 1.51 | 2.41 |
| Rspo2 | Meta | R-spondin 2 homolog (Xenopus laevis) | 2.46 | 1.52 | 3.89 |
| S1pr1 | Meta | sphingosine-1-phosphate receptor 1 | 2.03 | 2.59 | 2.41 |
| S1pr3 | Meta | sphingosine-1-phosphate receptor 3 | 2.78 | 2.93 | 3.46 |
| Sgcd | Meta | sarcoglycan, delta (35kDa dystrophin-associated glycoprotein) | 1.54 | 1.19 | 2.10 |
| Ssh1 | Meta | slingshot homolog 1 (Drosophila) | 1.71 | 2.17 | 2.06 |
| Stk17b | Meta | serine/threonine kinase 17b | 2.53 | 2.47 | 2.89 |
| Synj2 | Meta | synaptojanin 2 | 2.24 | 3.17 | 3.83 |
| Tie1 | Meta | tyrosine kinase with immunoglobulin-like and EGF-like domains 1 | 1.91 | 2.06 | 2.16 |
| Tmem196 | Meta | transmembrane protein 196 | 1.17 | 1.06 | 2.11 |
| Tpm4 | Meta | tropomyosin 4 | 2.18 | 2.51 | 2.57 |
| Tpp1 | Meta | tripeptidyl peptidase I | 1.61 | 2.23 | 2.06 |
| Tppp | Meta | tubulin polymerization promoting protein | 1.25 | 2.82 | 4.65 |
| Tppp3 | Meta | tubulin polymerization-promoting protein family member 3 | 2.31 | 5.01 | 6.60 |
| Tspan18 | Meta | tetraspanin 18 | 1.83 | 2.05 | 2.06 |
| Tspo | Meta | translocator protein (18kDa) | 2.82 | 3.10 | 2.95 |
| Vim | Meta | vimentin | 2.03 | 2.36 | 2.58 |
| Wdfy1 | Meta | WD repeat and FYVE domain containing 1 | 1.48 | 2.06 | 1.97 |
| Zeb2 | Meta | zinc finger E-box binding homeobox 2 | 2.04 | 1.78 | 2.18 |
| Adap2 | Other | ArfGAP with dual PH domains 2 | 2.55 | 1.83 | 2.60 |
| Aebp1 | Other | AE binding protein 1 | 1.02 | 1.44 | 2.20 |
| Antxr1 | Other | anthrax toxin receptor 1 | 1.91 | 2.76 | 4.30 |
| Anxa2 | Other | annexin A2 | 2.39 | 3.53 | 3.43 |
| Aplp1 | Other | amyloid beta (A4) precursor-like protein 1 | 1.58 | 2.48 | 2.86 |
| Arrdc3 | Other | arrestin domain containing 3 | 1.47 | 2.07 | 2.38 |
| Arrdc4 | Other | arrestin domain containing 4 | 1.58 | 1.85 | 2.10 |
| Bnc2 | Other | basonuclin 2 | 2.38 | 2.30 | 2.58 |
| Cachd1 | Other | cache domain containing 1 | 1.79 | 1.58 | 2.02 |
| Ccdc109b | Other | coiled-coil domain containing 109B | 1.22 | 1.70 | 2.37 |
| Ccnyl1 | Other | cyclin Y-like 1 | 2.22 | 2.44 | 2.42 |
| Cdig2 | Other | Cdig2 protein | 1.34 | 2.28 | 2.15 |
| Cntn1 | Other | contactin 1 | 1.26 | 1.52 | 2.90 |
| Coro2b | Other | coronin, actin binding protein, 2B | 1.46 | 2.09 | 2.58 |
| Crip2 | Other | cysteine-rich protein 2 | 1.30 | 2.11 | 2.41 |
| Dclk1 | Other | doublecortin-like kinase 1 | 3.70 | 3.44 | 3.62 |
| Ddc8 | Other | differential display clone 8 | 1.40 | 1.56 | 2.15 |
| Dfna5h | Other | deafness, autosomal dominant 5 | 1.25 | 2.07 | 2.04 |
| Dll4 | Other | delta-like 4 (Drosophila) | 1.24 | 2.00 | 2.09 |
| Dok2 | Other | docking protein 2, 56kDa | 1.76 | 1.80 | 2.26 |
| Dynlt3 | Other | dynein, light chain, Tctex-type 3 | 1.29 | 1.70 | 2.09 |
| Eda2r | Other | ectodysplasin A2 receptor | 1.51 | 1.36 | 2.35 |
| Ednra | Other | endothelin receptor type A | 2.68 | 2.68 | 3.71 |
| Ednrb | Other | endothelin receptor type B | 1.46 | 1.80 | 2.58 |
| Emp1 | Other | epithelial membrane protein 1 | 1.24 | 2.56 | 3.08 |
| Epha2 | Other | EPH receptor A2 | 1.70 | 1.72 | 2.53 |
| Esam | Other | endothelial cell adhesion molecule | 1.66 | 1.94 | 2.82 |
| Eya1 | Other | eyes absent homolog 1 (Drosophila) | 1.82 | 2.35 | 2.38 |
| Fam102b | Other | family with sequence similarity 102, member B | 1.95 | 2.86 | 2.87 |
| Fam126a | Other | family with sequence similarity 126, member A | 1.71 | 1.71 | 2.41 |
| Fam129a | Other | family with sequence similarity 129, member A | 2.04 | 2.04 | 5.87 |
| Fam171b | Other | family with sequence similarity 171, member B | 1.72 | 2.15 | 2.78 |
| Fam49a | Other | family with sequence similarity 49, member A | 1.40 | 1.99 | 3.52 |
| Fam62c | Other | family with sequence similarity 62 (C2 domain containing), member C | 1.60 | 3.52 | 3.41 |
| Fam69a | Other | family with sequence similarity 69, member A | 2.16 | 3.33 | 3.11 |
| Fgd6 | Other | FYVE, RhoGEF and PH domain containing 6 | 2.09 | 2.96 | 2.83 |
| Frmd6 | Other | FERM domain containing 6 | 1.45 | 1.73 | 2.20 |
| Galr2 | Other | galanin receptor 2 | 1.19 | 1.73 | 2.17 |
| Gm2a | Other | GM2 ganglioside activator | 1.61 | 2.13 | 2.11 |
| Gpr116 | Other | G protein-coupled receptor 116 | 1.61 | 1.33 | 2.17 |
| Gpr34 | Other | G protein-coupled receptor 34 | 2.50 | 1.24 | 2.24 |
| Gpr4 | Other | G protein-coupled receptor 4 | 1.52 | 2.64 | 2.31 |
| Hpcal1 | Other | hippocalcin-like 1 | 1.66 | 2.65 | 2.54 |
| Kremen1 | Other | kringle containing transmembrane protein 1 | 1.31 | 2.34 | 2.19 |
| Lancl3 | Other | LanC lantibiotic synthetase component C-like 3 (bacterial) | 1.52 | 2.33 | 2.23 |
| Laptm4b | Other | lysosomal protein transmembrane 4 beta | 1.95 | 2.46 | 2.64 |
| Lmo1 | Other | LIM domain only 1 (rhombotin 1) | 2.04 | 1.81 | 2.04 |
| Lnx2 | Other | ligand of numb-protein X 2 | 1.40 | 2.06 | 1.80 |
| Lrrc17 | Other | leucine rich repeat containing 17 | 7.83 | 9.17 | 10.37 |
| Lrrn4cl | Other | LRRN4 C-terminal like | 1.07 | 1.51 | 5.25 |
| Mcam | Other | melanoma cell adhesion molecule | 2.21 | 2.70 | 3.58 |
| Megf10 | Other | multiple EGF-like-domains 10 | 2.79 | 2.88 | 2.98 |
| Mitf | Other | microphthalmia-associated transcription factor | 2.33 | 2.92 | 2.99 |
| Morc4 | Other | MORC family CW-type zinc finger 4 | 2.07 | 1.71 | 2.25 |
| Mrgprf | Other | MAS-related GPR, member F | 1.03 | 1.76 | 2.28 |
| Msln | Other | mesothelin | 1.27 | 2.11 | 1.88 |
| Myo1d | Other | myosin ID | 1.84 | 3.07 | 2.82 |
| Myof | Other | myoferlin | 3.49 | 5.41 | 5.30 |
| Nav1 | Other | neuron navigator 1 | 2.18 | 1.92 | 2.20 |
| Ndrg4 | Other | NDRG family member 4 | 1.98 | 2.28 | 2.38 |
| Nid1 | Other | nidogen 1 | 1.79 | 2.16 | 2.39 |
| Nrarp | Other | NOTCH-regulated ankyrin repeat protein | 1.68 | 2.06 | 1.93 |
| Olfml3 | Other | olfactomedin-like 3 | 2.72 | 2.42 | 3.00 |
| Olr63 | Other | olfactory receptor, family 51, subfamily E, member 1 | 2.33 | 3.31 | 5.05 |
| Opn3 | Other | opsin 3 | 1.73 | 2.16 | 2.10 |
| P2ry12 | Other | purinergic receptor P2Y, G-protein coupled, 12 | 2.62 | 1.44 | 2.72 |
| Pdlim1 | Other | PDZ and LIM domain 1 | 2.60 | 2.67 | 3.32 |
| Pftk1 | Other | PFTAIRE protein kinase 1 | 2.12 | 1.80 | 2.22 |
| Phlda3 | Other | pleckstrin homology-like domain, family A, member 3 | 2.16 | 2.58 | 2.74 |
| Plec1 | Other | plectin 1, intermediate filament binding protein 500kDa | 1.67 | 2.48 | 2.75 |
| Plekhf1 | Other | pleckstrin homology domain containing, family F (with FYVE domain) member 1 | 1.36 | 1.94 | 2.05 |
| Plekhg4 | Other | pleckstrin homology domain containing, family G (with RhoGef domain) member 4 | 2.53 | 4.24 | 3.95 |
| Plxna2 | Other | plexin A2 | 1.72 | 2.44 | 2.40 |
| Prrx2 | Other | paired related homeobox 2 | 1.65 | 2.30 | 2.90 |
| Prss35 | Other | protease, serine, 35 | 1.70 | 3.16 | 3.21 |
| Rai14 | Other | retinoic acid induced 14 | 1.80 | 1.95 | 2.17 |
| Rgs5 | Other | regulator of G-protein signaling 5 | 1.50 | 2.28 | 5.78 |
| S100a10 | Other | S100 calcium binding protein A10 | 1.78 | 3.01 | 2.88 |
| Scara5 | Other | scavenger receptor class A, member 5 (putative) | 1.53 | 2.45 | 3.17 |
| Scrn1 | Other | secernin 1 | 1.17 | 1.22 | 2.54 |
| Sh3bgrl2 | Other | SH3 domain binding glutamic acid-rich protein like 2 | 2.08 | 2.02 | 2.13 |
| Slfn5 | Other | schlafen family member 5 | 2.07 | 1.36 | 2.31 |
| Slit3 | Other | slit homolog 3 (Drosophila) | 1.38 | 2.29 | 2.21 |
| Sync | Other | syncoilin, intermediate filament protein | 1.62 | 1.75 | 2.29 |
| Tagln | Other | transgelin | 3.44 | 3.73 | 4.80 |
| Tbc1d2b | Other | TBC1 domain family, member 2B | 1.96 | 2.21 | 2.58 |
| Tessp6 | Other | similar to 1700112C13Rik protein | 1.11 | 1.90 | 2.23 |
| Tmem100 | Other | transmembrane protein 100 | 2.03 | 2.07 | 3.92 |
| Tmem119 | Other | transmembrane protein 119 | 1.40 | 2.23 | 2.55 |
| Tmem184c | Other | transmembrane protein 184C | 2.46 | 2.36 | 3.15 |
| Tmem200a | Other | transmembrane protein 200A | 1.73 | 1.57 | 2.17 |
| Tmtc3 | Other | transmembrane and tetratricopeptide repeat containing 3 | 2.06 | 2.51 | 2.43 |
| Trim16 | Other | tripartite motif-containing 16 | 1.61 | 2.46 | 2.85 |
| Tspan11 | Other | tetraspanin 11 | 1.08 | 1.66 | 2.68 |
| Znf622 | Other | zinc finger protein 622 | 1.55 | 2.02 | 3.51 |
| Abcb1b | Transporter | ATP-binding cassette, sub-family B (MDR/TAP), member 1B | 1.88 | 1.49 | 2.59 |
| Abcc4 | Transporter | ATP-binding cassette, sub-family C (CFTR/MRP), member 4 | 1.40 | 2.27 | 1.94 |
| Abcc9 | Transporter | ATP-binding cassette, sub-family C (CFTR/MRP), member 9 | 2.18 | 2.42 | 4.15 |
| Aqp1 | Transporter | aquaporin 1 (Colton blood group) | 1.48 | 2.66 | 4.40 |
| Atp2c1 | Transporter | ATPase, Ca++ transporting, type 2C, member 1 | 1.99 | 2.17 | 2.03 |
| Atp6v1g1 | Transporter | ATPase, H+ transporting, lysosomal 13kDa, V1 subunit G1 | 1.51 | 2.07 | 1.86 |
| Cacna1c | Transporter | calcium channel, voltage-dependent, L type, alpha 1C subunit | 2.43 | 2.90 | 2.86 |
| Cacna1g | Transporter | calcium channel, voltage-dependent, T type, alpha 1G subunit | 1.23 | 1.84 | 2.00 |
| Clcn6 | Transporter | chloride channel 6 | 1.79 | 1.76 | 2.07 |
| Clic2 | Transporter | chloride intracellular channel 2 | 1.62 | 1.53 | 2.16 |
| Ctbs | Transporter | chitobiase, di-N-acetyl- | 1.58 | 1.86 | 2.04 |
| Dpp6 | Transporter | dipeptidyl-peptidase 6 | 1.82 | 1.45 | 2.42 |
| Folr2 | Transporter | folate receptor 2 (fetal) | 3.11 | 3.73 | 4.04 |
| Gja1 | Transporter | gap junction protein, alpha 1, 43kDa | 2.42 | 3.06 | 3.02 |
| Gria3 | Transporter | glutamate receptor, ionotrophic, AMPA 3 | 1.19 | 1.05 | 2.35 |
| Kcne3 | Transporter | potassium voltage-gated channel, Isk-related family, member 3 | 2.71 | 3.54 | 5.89 |
| Kcne4 | Transporter | potassium voltage-gated channel, Isk-related family, member 4 | 1.80 | 1.36 | 2.71 |
| Kcnj15 | Transporter | potassium inwardly-rectifying channel, subfamily J, member 15 | 1.91 | 2.56 | 4.10 |
| Orai2 | Transporter | ORAI calcium release-activated calcium modulator 2 | 1.97 | 2.03 | 1.98 |
| P2rx4 | Transporter | purinergic receptor P2X, ligand-gated ion channel, 4 | 1.54 | 1.70 | 2.28 |
| Pea15a | Transporter | phosphoprotein enriched in astrocytes 15 | 2.15 | 1.90 | 2.22 |
| Plp2 | Transporter | proteolipid protein 2 (colonic epithelium-enriched) | 1.69 | 2.10 | 2.25 |
| Rufy1 | Transporter | RUN and FYVE domain containing 1 | 1.60 | 1.96 | 2.05 |
| Sfxn3 | Transporter | sideroflexin 3 | 1.15 | 2.02 | 1.73 |
| Slc13a3 | Transporter | solute carrier family 13 (sodium-dependent dicarboxylate transporter), member 3 | 1.05 | 1.92 | 2.02 |
| Slc16a12 | Transporter | solute carrier family 16, member 12 (monocarboxylic acid transporter 12) | 1.35 | 1.77 | 2.27 |
| Slc2a12 | Transporter | solute carrier family 2 (facilitated glucose transporter), member 12 | 1.87 | 1.59 | 3.03 |
| Slc2a3 | Transporter | solute carrier family 2 (facilitated glucose transporter), member 3 | 3.67 | 4.15 | 3.78 |
| Slc2a9 | Transporter | solute carrier family 2 (facilitated glucose transporter), member 9 | 1.36 | 1.90 | 2.22 |
| Slc39a1 | Transporter | solute carrier family 39 (zinc transporter), member 1 | 1.37 | 2.06 | 1.96 |
| Slc39a10 | Transporter | solute carrier family 39 (zinc transporter), member 10 | 1.83 | 1.84 | 2.16 |
| Slc43a2 | Transporter | solute carrier family 43, member 2 | 1.56 | 2.42 | 2.32 |
| Slc6a15 | Transporter | solute carrier family 6 (neutral amino acid transporter), member 15 | 1.50 | 2.52 | 3.54 |
| Slc8a1 | Transporter | solute carrier family 8 (sodium/calcium exchanger), member 1 | 1.98 | 2.36 | 2.41 |
| Slco3a1 | Transporter | solute carrier organic anion transporter family, member 3A1 | 1.17 | 2.00 | 2.29 |
| Snx7 | Transporter | sorting nexin 7 | 1.39 | 1.77 | 2.03 |
| Sorcs2 | Transporter | sortilin-related VPS10 domain containing receptor 2 | 1.93 | 2.65 | 2.68 |
| Tcn2 | Transporter | transcobalamin II; macrocytic anemia | 2.00 | 2.61 | 3.15 |
| Tinagl1 | Transporter | tubulointerstitial nephritis antigen-like 1 | 1.20 | 1.90 | 2.53 |
| Trpc6 | Transporter | transient receptor potential cation channel, subfamily C, member 6 | 2.15 | 2.19 | 3.29 |
| Vat1 | Transporter | vesicle amine transport protein 1 homolog (T. californica) | 1.75 | 2.37 | 2.74 |
| Vps29 | Transporter | vacuolar protein sorting 29 homolog (S. cerevisiae) | 1.66 | 1.93 | 2.06 |
